# The deubiquitylase OTUB1 drives gemcitabine resistance in pancreatic cancer by enhancing pyrimidine metabolism through modulating DHODH mRNA stability

**DOI:** 10.1038/s41419-025-08001-4

**Published:** 2025-10-06

**Authors:** Wenming Zhang, Rui Liu, Junwen Hu, Shuangyan Wan, Yeqin Zou, Tong Che, Jin Zhang, Leifeng Chen, Xiaogang Peng

**Affiliations:** 1 Department of General Surgery, The Second Affiliated Hospital of Nanchang University, Nanchang, Jiangxi Province China; 2 Jiangxi Key Laboratory of Molecular Medicine, The Second Affiliated Hospital of Nanchang University, Nanchang, Jiangxi Province China; 3 Human Aging Research Institute, School of Life Sciences, Nanchang University, Nanchang, Jiangxi Province China; 4 School of Basic Medical Sciences, Jiangxi Medical College, Nanchang University, Nanchang, Jiangxi Province China; 5 Department of Genetic Medicine, The Second Affiliated Hospital of Nanchang University, Nanchang, Jiangxi Province China; 6 Department of Oncology, The Second Affiliated Hospital of Nanchang University, Nanchang, Jiangxi Province China

**Keywords:** Chemotherapy, Post-translational modifications

## Abstract

Gemcitabine resistance is a major clinical challenge in pancreatic cancer (PC); therefore, strategies to combat gemcitabine resistance are urgently required. Reprogramming pyrimidine metabolism by oncogenic signaling contributes to cancer progression and confers chemoresistance to many cancers. The current study identified the deubiquitinating enzyme OTUB1 as a promising therapeutic target for combating gemcitabine resistance in PC. OTUB1 was found to be aberrantly expressed in PC and remarkably correlated with poor patient survival. Both in vivo and in vitro, OTUB1 knockdown increased the gemcitabine efficacy of PC cells by inhibiting pyrimidine metabolism. Furthermore, OTUB1 enhanced de novo nucleotide pyrimidine synthesis in PC cells by upregulating dihydroorotate dehydrogenase (DHODH), a critical rate-limiting enzyme for pyrimidine de novo biosynthesis. Mechanistically, OTUB1 suppressed the degradation and polyubiquitination of the RNA-binding protein DEAD-box helicase 3 X-linked (DDX3X), which in turn stabilized DDX3X-mediated DHODH mRNA. OTUB1 interacts with DDX3X, and the binding stabilizes DDX3X through its deubiquitinase activity. In addition, a small-molecule OTUB1 inhibitor combined with gemcitabine treatment could synergistically inhibit tumor growth in high-OTUB1-expressing murine tumoroids. Collectively, OTUB1 could impart gemcitabine resistance by promoting de novo pyrimidine synthesis, and targeted suppression of OTUB1 could be an effective strategy to overcome gemcitabine resistance in PC.

## Introduction

Pancreatic cancer (PC) is among the worst cancers, with a favorable 5-year survival rate of only 10%, since almost 80% of the patients with PC present with an incurable illness and are diagnosed at an advanced stage [1]. Currently, the best treatment regimen for such patients is gemcitabine plus adjuvant chemotherapy with nab-paclitaxel [2]. Although gemcitabine is a widespread first-line treatment option for PC, intrinsic and acquired drug resistance are the main obstacles that restrict the clinical efficacy of gemcitabine-based therapies [3, 4]. Therefore, understanding the mechanisms underlying gemcitabine resistance can aid the development of novel combination therapeutic approaches for patients with PC.

In the last decade, several cancer therapies have focused on adaptive metabolic reprogramming, which supports nucleic acid synthesis [5]. Resistant cancer cells rely on metabolic pathways to overcome pharmacological stress and DNA replication arrest by nucleotide analogs such as gemcitabine [6]. Tumor cells maintain the intracellular nucleotide pool by facilitating the biosynthesis of de novo nucleotides and thus compete with nucleotide analogs [7, 8]. In particular, increasing evidence has revealed a crosstalk between pyrimidine metabolic reprogramming and chemoresistance. For instance, to stimulate carcinogenesis and chemoresistance of colorectal cancer, CSN6 mediates nucleotide metabolism [9]. Notably, activation of the pyrimidine metabolic pathway plays a key role in gemcitabine resistance in PC. Gemcitabine-resistant (GR) PC cells have remarkably enhanced pyrimidine de novo biosynthesis compared to gemcitabine-sensitive cells, and pharmacological inhibition of pyrimidine de novo biosynthesis sensitized the cancer cells to chemotherapy [10]. Upregulation of dihydroorotate dehydrogenase (DHODH), a critical rate-limiting enzyme in the pyrimidine biosynthesis pathway, leads to gemcitabine resistance in PC [11]. Thus, disrupting the elevation of pyrimidine nucleotides, such as by eliminating enzymes involved in de novo pyrimidine biosynthesis, could be a promising strategy to counter gemcitabine resistance in PC.

Deubiquitinases (DUBs) are proteolytic enzymes that recognize and remove ubiquitin molecules from protein substrates [12]. Among the DUBs, the subfamily of ovarian tumor (OTU) DUBs has been identified as a regulator of crucial signaling cascades involved in the progression of many cancers [13]. As a member of the OTU DUB family, OTUB1 has been implicated in immune response, neurodegenerative disorders, and DNA damage response [14, 15]. Emerging evidence has revealed that poor clinical outcomes in multiple cancers are directly linked to OTUB1, which is substantially upregulated in such cases [16, 17]. By suppressing the Hippo signaling pathway, OTUB1 enhances the stemness and proliferation of gastric cancer cells [18]. By preventing PD-L1 from being degraded by the endoplasmic reticulum, OTUB1 promotes immune suppression of cancer cells [19]. Several studies have reported that targeting OTUB1 may inhibit cancer progression and immune disorders [20]. Therefore, as a feasible and attractive therapeutic target, OTUB1 has great potential in alleviating tumor chemotherapy resistance and is worth further exploration.

In this study, we found that high-OTUB1-expressing PC cells exhibit higher resistance to gemcitabine owing to elevated pyrimidine metabolism. We showed that OTUB1 deubiquitinated and stabilized the DEAD-box helicase 3 X-linked (DDX3X), an important regulator of mRNA stabilization [21], which in turn stabilized DHODH mRNA and subsequently increased DHODH expression with a concomitant increase in de novo pyrimidine biosynthesis. Co-administration of OTUB1 inhibitor with gemcitabine abrogated the OTUB1-mediated resistance in high-OTUB1-expressing patient-derived xenograft (PDX) mouse models. Therefore, according to our findings, OTUB1 suppression may be a potential combination therapy plan for patients with PC who are resistant to gemcitabine.

## Materials and methods

### Patients and tumor specimens

All tissue samples that were formalin-fixed, snap-frozen, and embedded in paraffin were from 86 patients who had surgery at the Second Affiliated Hospital of Nanchang University. All patients gave written informed permission for the collection of specimens, and the study protocol was approved by the ethics committee of the Second Affiliated Hospital of Nanchang University ([2019] No. (053)). All specimens were pathologically confirmed as pancreatic ductal adenocarcinoma by expert pathologists and exclusively obtained from patients without prior gemcitabine chemotherapy exposure. And, a summary of each patient’s clinical features, including age, gender distribution, tumor size, TNM stage, distant metastasis and differentiation, is now summarized in Table 1.

**Table 1:** Relationship between OTUB1 expression and clinicopathological features.

Parameters	Group	*n*	OTUB1 expression		*P* value
			Low (*N* = 28)	High (*N* = 58)	
Age (years)					*P* = 0.1186
	Male	56	15	41	
	Female	30	13	17	
Gender					*P* = 0.1916
	≤65	23	10	13	
	å 65	63	18	45	
T					*P* = 0.1635
	T1–T2	65	15	40	
	T3-T4	21	13	18	
N					*P* = 0.3607
	0	31	12	19	
	1–2	55	16	39	
M					*P* = 0.7859
	0	57	18	39	
	1	29	10	19	
Stage					***P*** = **0.0027**
	IA–IIB	58	25	33	
	Ⅲ–Ⅳ	28	3	25	
Tumor size					***P*** = **0.0213**
	≤3	49	11	38	
	>3	37	17	20	
Distant metastasis					*P* = 0.7902
	No	66	21	45	
	Yes	20	7	13	
Differentiation					
	Well Moderate/poor	29 57	23 5	23 35	***P*** = **0.0002**

Bold values indicate significant differences.

### Cell culture

Human PC cell lines, encompassing CFPAC-1, PANC-1, AsPC-1, MIA PaCa-2, BXPC-3, HuPT-3, Panc0403 and SU86.86 were obtained from ATCC (Manassas, VA, USA). HEK-293T was procured from the Chinese Academy of Sciences cell bank. The culture media applied for HEK-293T, MIA PaCa-2, and PANC-1 included 10% FBS in addition to DMEM. CFPAC-1, HuPT-3, Panc0403, SU86.86, AsPC-1, and BXPC-3 were cultivated in RPMI-1640 (Gibco, Life Technologies) with 10% FBS. In addition, gemcitabine (GEM)-resistant PANC-1/GR were generated by exposing GEM-sensitive PANC-1 parental cells to an initial concentration (0.002 μM) for 1 week. When cells resumed normal growth following the recovery period, the GEM concentration was progressively increased from the initial concentration 0.1 μM over a 10-month period. All of the cell lines were kept at 37 °C and 5% CO_2_ in a humidified incubator.

### Quantitative real-time PCR (qRT-PCR) analysis

Total RNA was isolated from tumor cells and tissues using the TRIzol reagent (Invitrogen, USA). In accordance with the manufacturer’s instructions, the extracted mRNA was transformed into cDNA and subjected to qRT-PCR using a two-step SYBR Green qRT-PCR kit (Takara, Dalian, China). Gene expression levels were normalized to GAPDH expression levels in each sample. The relative levels of gene expression were calculated using 2^−∆∆Ct^ method. Table S1 presents the primer sequences used for qRT-PCR.

### Western blotting, immunofluorescence (IF), and immunohistochemistry (IHC)

Equal volumes of total protein were separated using SDS-PAGE and transferred onto PVDF membranes for western blot analysis. Membranes were treated with 5% skim milk to block non-specific binding and then treated with primary antibody and the proper HRP-conjugated secondary antibodies for subsequent determination by chemiluminescence (Bio-Rad). The following antibodies were applied: antibodies against OTUB1 (1:1000, Abcam, ab270959), DDX3X (1:1000, Abcam, ab196032), DHODH (1:1000, Abcam, ab174288), Flag (1:1000, Sigma, F1804), Caspase-3 (1:1000, Abcam, ab13847), cleaved caspase-3 (1:1000, Abcam, ab2302), GAPDH (1:1000, Abcam, ab8245), ubiquitin (1:1000, Enzo Life Sciences, PW8805), and HA (1:1000, Abmart, M20003). Samples of normal and malignant pancreatic tissues embedded in paraffin were cut into 5-µm-thick sections for IHC analysis. Sections were boiled in citrate buffer and rehydrated. They were blocked with 5% BSA, and subsequently treated with the primary antibody and horseradish peroxidase-conjugated secondary antibody. Finally, the sections were stained using a DAB Detection Kit, in accordance with the manufacturer’s instructions. Antibodies were used for immunohistochemical detection of Ki-67 (1:200, Cell Signaling, 2586), cleaved caspase-3 (1:200, Abcam, ab2302), and OTUB1 (1:200, ab270959; Abcam). Cells were fixed in 4% paraformaldehyde (PFA) and inoculated with 0.1% TritonX-100 for IF analysis. Following 5% BSA blocking, the relevant primary and secondary antibodies were used to stain the samples. Antibodies used in IF were anti-DDX3X (1:100, Abcam, ab196032), anti-OTUB1 (1:100, Abcam, ab270959), and anti-HA (1:200, CST, #2367).

### Overexpression plasmids and short hairpin RNA (shRNA)

The overexpression plasmids used to generate recombinant lentiviruses, including pLVX-Puro-OTUB1, pLVX-Puro-OTUB1^C91A^, pLVX-Puro-DHODH, and pLVX-Puro-DXX3X, were constructed by GenePharma (Shanghai, China) and confirmed by direct DNA sequencing. Lentivirus-based shRNAs specific for DDX3X, DHODH, and OTUB1, or interference controls, were synthesized by GenePharma (Shanghai, China) and cloned into a pLKO.1-1TRC vector. For stable knockdown, PC cells were transformed with the relevant shRNA lentivirus for 24 h and subsequently cultured in fresh medium containing puromycin for 1 week. Following verification by western blotting and qRT-PCR, selected cells were cultured and employed in further experiments. Table S2 contains all shRNA sequences.

### Chemosensitivity assay, apoptosis analysis, and colony formation assay

Specified cells were subjected to gradually increasing concentrations of gemcitabine for 48 h to perform chemosensitivity experiments. The CCK-8 assay was used to examine cell viability in accordance with the manufacturer’s instructions. To calculate the IC_50_ value, data were analyzed using GraphPad Prism 8, and a dose-response curve was drawn. For apoptosis analysis, gemcitabine was administered at varying doses to the indicated cells for 36 h. Cell apoptosis was examined using a flow cytometer and an Annexin V-FITC/7-AAD apoptosis detection kit. Apoptotic cells were those that were stained with both 7-AAD and annexin V, whereas viable cells were those that were not stained with either of these substances. The appropriate cells were transfected with shRNA plasmids or overexpression constructs, selected after 4–6 days of culture, and plated in a 6-well plate (2000 cells per well) for the colony formation assay. Following 2–3 weeks of culture, the cells were stained with 0.5% crystal violet solution and subsequently inoculated with fixation buffer (5% methanol and 5% acetic acid).

### Deoxyribonucleoside triphosphate assay

Deoxyribonucleoside triphosphate levels were measured as described previously [22]. Briefly, PC cells were cultured in RPMI-1640 medium. In order to extract the metabolites, the medium was aspirated after 24 h and ice-cold 60% (v/v) methanol was added. After that, the cells were scraped and centrifuged at 15,000 rpm for 10 min at 4 °C. The resulting metabolite-containing supernatants were evaporated and resuspended in water for further analysis.

### [U-^13^C_6_]-Glucose tracing and LC/MS-MS metabolomics profiling

PC cells were grown in glucose-free RPMI-1640 medium supplemented with 11 mmol/L stable isotope D-Glucose-^13^C_6_ (Cambridge Isotope, CLM-1396-1) and 10% dialyzed FBS (Gibco, #30067334). Following a 24-h incubation period, cell plates were cleaned with ice-cold PBS containing 1% fatty acid-free bovine serum albumin (FAF-BSA; 126575, Sigma), and metabolites were separated using 80% ice-cold methanol. Thereafter, the cells were scraped and centrifuged at 15,000 rpm for 15 min at 4 °C. The cell pellet was used to measure protein levels to normalize the metabolite levels, and the supernatant was transported to LC-MS vials. Finally, a Dionex UltiMate 3000 LC System (Thermo Fisher Scientific) connected to a Q-Exactive Orbitrap mass spectrometer (Thermo Fisher Scientific) was used to evaluate the metabolites extracted from appropriate cells following the instructions provided. The abundance of metabolites was normalized to the protein content and displayed relative to the internal standard.

#### Cell line-derived xenograft (CDX) and PDX models

For CDX models, the firefly luciferase gene was stably transduced into PC cells for injection into the CDX animal models, allowing frequent in vivo monitoring of tumor growth by bioluminescent imaging using a Lumina Series III IVIS (In Vivo Imaging System) device (PerkinElmer, MA, USA). Male BALB/c-nu/nu mice, aged 6–8 weeks, were subcutaneously injected with 1 × 10^6^ cells in their flanks. When the tumor volume reached ~100 mm^3^, GEM (50 mg/kg/week) was injected into the mice intraperitoneally. The tumor size was measured every five days. For CDX models, tumor tissues from patients with PC were obtained and sliced into tiny 3-mm^3^-pieces and subcutaneously inserted into the axilla of 6-week-old female M-NSG mice. The mice were randomly assigned to four groups after the tumor volume reached 150 mm^3^, namely control, GEM plus batefenterol, batefenterol (intraperitoneal, 20 mg/kg/day), and GEM (intraperitoneal, 50 mg/kg/week). Before euthanasia, the diameter of the tumor was determined once a day, and a tumor volume of 1500 mm^3^ was considered to indicate death. Furthermore, tumor tissues from the PDX or CDX mouse model were embedded in paraffin and preserved in 4% PFA for further analysis. All animal experiments were conducted in accordance with the protocols approved by the Animal Experimental Ethics Committee of Nanchang University (NCUFII-2021468) and the procedures set forth in the NIH Guide for the Care and Use of Laboratory Animals.

### Actinomycin D assay

A 6-well plate containing ~1 × 10^5^ PC cells per well was prepared, and at the designated time intervals, actinomycin D (APExBIO, #A4448) was added. After harvesting the cells, total RNA was separated using qRT-PCR to assess gene expression.

### In vivo ubiquitination assay and co-immunoprecipitation (co-IP) assay

For the in vivo ubiquitination assay, the indicated expression plasmids or recombinant lentiviruses were transfected into HEK-293T or PC cells. Following transfection, the cells were exposed to the proteasome inhibitor MG132 for 6 h, and the following steps were conducted in accordance with the earlier instructions. Cell lysates were extracted from the indicated cells for the co-IP assay and then inoculated with a matching primary antibody and protein A/G-Sepharose beads. Subsequently, the proteins that coprecipitated were gathered and determined by western blotting.

### Virtual docking

Virtual docking was conducted using the Schrödinger Suite 2021-2 (Schrödinger, LLC). Briefly, (1) the OTUB structural model (PDB: 4DDI) was processed using the default settings within the Protein Preparation Wizard, (2) the active site Cys91 was determined as the binding area for small-molecule compound screening using the Receptor Grid Generation Wizard, and (3) the compounds used for this screening were sourced from the MCE Bioactive Compound Library (HY-L001V, total 23,562 compounds). The small-molecule structures were generated and optimized using the OPLS4 force field with the LigPrep program. (4) Molecular docking was performed through a three-step screening process using the Glide program. First, a high-throughput virtual screening program was employed to screen the compounds, and the first 3000 candidates were selected based on their docking scores. Next, these were re-docked utilizing the standard accuracy and Prime MM-GBSA studies. Finally, 20 compounds were selected and processed for subsequent activity assessment.

### Surface plasmon resonance (SPR)

The SPR assay for inhibitors targeting OTUB1 was performed using the sensor chip CM5 (GE Healthcare, USA) and Biacore 8 K (Cytiva). Recombinant human OTUB1 protein (Abcam, ab157086) was immobilized on the Fc2 channel via the CM5 chip amino coupling approach, while the Fc1 channel served as the reference channel for blocking and activation. Recombinant human OTUB1 typically has a protein-coupling capacity of approximately 13800 RU. The following were the conditions for protein coupling: the system was a pH 4.0 sodium acetate solution, the concentration was around 50 μg/mL, the chip activation time was 900 s, and the blocking duration was 420 s. A 60-s dissociation and association time and a 30-μL/min flow rate were used.

### Statistical analysis

All data are represented as means ± standard error using GraphPad Prism 8 (GraphPad Software, USA). Two-tailed distributions and Student’s *t* tests were used to analyze the significant differences. The survival curve was computed using the Kaplan–Meier approach, and significance was assessed using the log-rank test. Statistical significance was set at *P* < 0.05.

## Results

### OTUB1 was identified as a predictor of poor prognosis in patients with PC

**Fig. 1:**
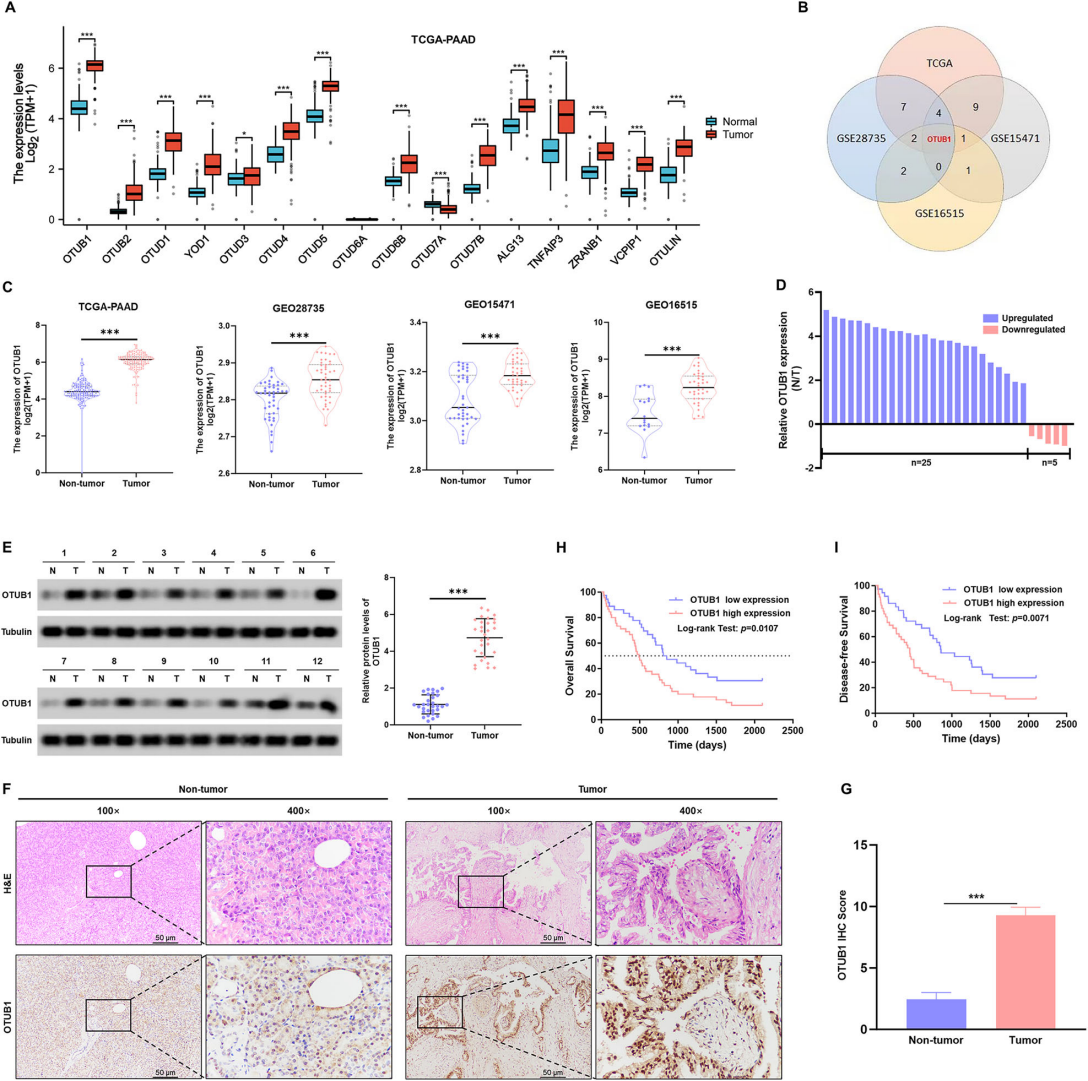
Elevated expression of OTUB1 is associated with poor PC patient prognosis. **A** TCGA-PAAD database analysis shows the 16 differentially expressed human OTU domain DUBs in PC tissues and their corresponding adjacent normal tissues. *p < 0.05; ***p < 0.001. **B** Venn diagram showing OTUB1 expression in the PCGA-PAAD, GEO28735, GEO16515, and GEO15471 dataset. **C** OTUB1 expression levels were significantly higher in the PC tissues compared to the adjacent normal tissues in all four datasets. **D** qRT-PCR analysis of OTUB1 mRNA expression in PC tumors and paired normal tissues. **E** Determination (left) and quantitation (right) of OTUB1 protein levels in PC tissues and paired normal tissues by Western blot assay. Tubulin was used as the internal standard. ***p < 0.001. **F**, **G** Re*p*resentative image (left) and quantitation (right) of OTUB1 staining were displayed in PC tissues and paired normal tissues (magnification ×100, inset magnification×400). Scale bar, 50 μm. ***p < 0.001. **H**, **I** Kaplan–Meier *p*lots representing probabilities of overall (H) and progression-free survival (I) in 86 PC patients with high- and low-OTUB1 expression.

To determine which members of the OTU family of DUBs play a role in PC tumorigenesis, we initially explored the expression levels of OTU DUBs in the publicly available TCGA-PAAD, GEO28735, GEO16515, and GEO15471 datasets (Fig. 1A, and Fig. S1A). Furthermore, we generated Venn diagrams of OTU DUBs with differential expression in the TCGA-PAAD, GEO28735, GEO16515, and GEO15471 datasets by removing the influence of tissue specificity and found OTUB1 significantly overexpressed in each overlapping region (Fig. 1C, D). At the same time, the frequency and location of copy number variation alterations in OTU DUBs are shown in Fig. S1B, C, respectively. Using a receiver operating characteristic analysis, we found the OTUB1 expression levels accurately distinguished PC tissues from the adjacent pancreatic tissues with an AUC value of 0.974 (Fig. S1D). Importantly, we also found that OTUB1 expression was closely correlated with a worse prognosis of PC in the TCGA-PAAD database (Fig. S1E, F). Therefore, we focused our investigation on the role of OTUB1 in the malignant progression of PC.

We evaluated the expression levels of OTUB1 in the in-house 86-paired PC samples. When PC tissues were compared to the neighboring normal tissues, there was a clear increase in the mRNA and protein levels of OTUB1 (Fig. 1D, E). Consistent with these findings, IHC data revealed that OTUB1 was elevated in 67.4% (58/86) of the PC tissue specimens (Fig. 1F, G). Next, we evaluated the relationship between the clinical characteristics and OTUB1 expression patterns in patients with PC. The data revealed that high-OTUB1 levels had a positive association with TNM stage (*P* = 0.0027), tumor size (*P* = 0.0213) and Differentiation (*P* = 0.0002) (Table 1). In addition, multivariate Cox regression analysis showed that high-OTUB1 level was an independent prognostic factor for poor survival of patients with PC (Table 2). Kaplan–Meier analysis showed that, in line with the findings from the TCGA database, patients with PC having high-OTUB1 expression had a considerably shorter overall survival (OS) time than those with low-OTUB1 expression (Fig. 1H, I). Overall, the findings indicate that elevated OTUB1 expression is strongly associated with poor clinical prognosis in patients with PC.

**Table 2:** Univariate and multivariate analyses of overall survival in PC patients.

Parameters	Univariate analysis HR	95% CI	*P* value	Multivariate analysis HR	95% CI	*P* value
Age (≥65 vs <65)	1.365	0.756–2.531	0.353	—	—	—
Sex (female vs male)	1.159	0.566–1.315	0.296	—	—	—
Distant metastasis (no vs yes)	1.527	0.891–3.729	0.631	—	—	—
Tumor size (<5 vs ≥6)	1.952	1.268–3.982	0.009*	1.598	1.096–2.596	0.038*
TNM stage (T1–T2 vs T3-–T4)	2.295	1.139–4.355	0.016*	1.793	1.115–3.961	0.043*
Differentiation (well vs moderate/poor)	1.686	1.713–3.679	0.007*	1.533	1.217–3.082	0.059
OTUB1 expression (high vs low)	5.167	2.139–5.200	0.001*	2.398	1.863–4.973	0.016*

*HR* hazard ratio, *CI* confidence interval.

*P < 0.05.

### Elevated expression of OTUB1 imparts gemcitabine resistance to PC cells

**Fig. 2:**
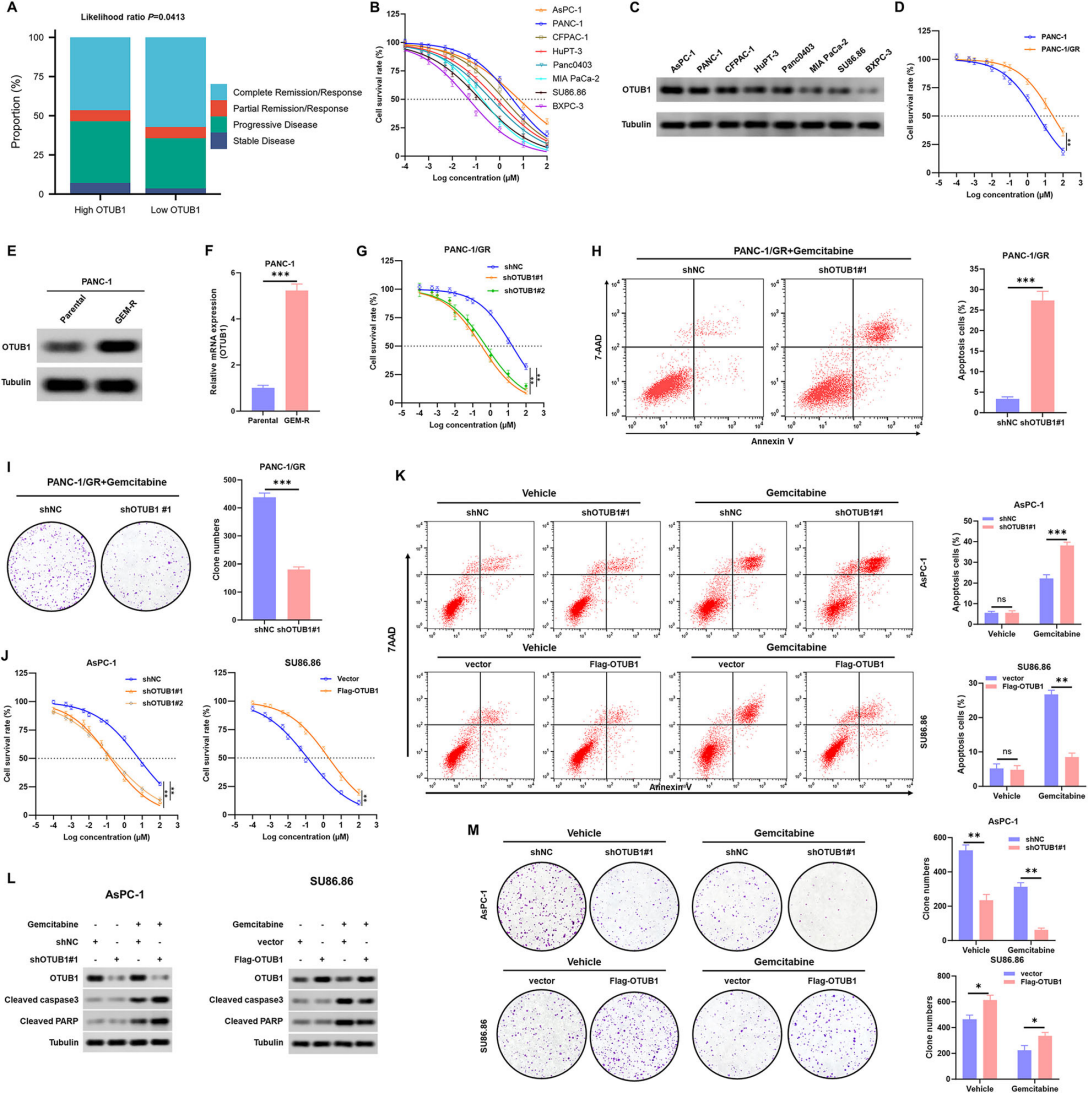
OTUB1 protein expression level is associated with PC cell sensitivity to gemcitabine. **A** Hierarchy graph demonstrating TCGA analysis on high and low-OTUB1-expressing PC patients with progressive disease and complete response after gemcitabine treatment. **B** Drug responses to gemcitabine of eight human PC cell lines. BxPC-3, PANC-1, AsPC-1, MIA PaCa-2, SU86.86, Panc0403, and HuPT-3 cells were exposed to various concentrations of gemcitabine (GEM) for 72 h, and cell viability was measured by CCK-8 assays. **C** Determination of OTUB1 protein levels in eight PC cell lines by Western blot assay. Tubulin was used as the internal standard. **D** IC50 (50% inhibitory concentration) value of gemcitabine in PANC-1 and PANC-1/GR cell lines was detected by CCK-8 assays. **p < 0.01. **E**, **F** The mRNA and protein levels of OTUB1 in PANC-1 and PANC-1/GR cell lines were detected by qRT-PCR and western blot assay, respectively. Tubulin was used as the internal standard. **p < 0.01, ***p < 0.001. **G** Sensitivity to gemcitabine in PANC-1/GR cells transfected with OTUB1 shRNA was detected by CCK-8 assay. **p < 0.01. **H** Determination (left) and quantitation (right) of the apoptosis rate in PANC-1/GR cells following OTUB1 knockdown by flow cytometry. ***p < 0.001. **I** Representative images (left) and quantitation (right) of colony formation assays in PANC-1/GR cells transfected with OTUB1 shRNA. ***p < 0.001. **J** Sensitivity to gemcitabine in the indicated cells was detected by CCK-8 assay. **p < 0.01. **K** Determination (left) and quantitation (right) of the a*p*optosis rate in the indicated cells by flow cytometry. **p < 0.01, ***p < 0.001. **L** Protein levels of OTUB1, Cleaved-caspase-3, and Cleaved-PARP were determined by western blot assays in the indicated cells upon treatment with or without gemcitabine. Tubulin was used as the internal standard. **M** Representative images (left) and quantitation (right) of colony formation assays in the indicated cells. *p < 0.05, **p < 0.01.

We aim to investigate whether OTUB1 was involved in the development of gemcitabine resistance, since the progression of clinical disease in PC invariably follows the emergence of gemcitabine resistance. We first explored the clinical relationship between OTUB1 and PC chemoresistance in the PAAD-TCGA cohort. As shown in Fig. 2A, patients with high-OTUB1 expression showed progressive disease after gemcitabine therapy, whereas those with low-OTUB1 expression were more responsive to gemcitabine. Then, we examined gemcitabine susceptibility in eight human cell lines, namely AsPC-1, PANC-1, CFPAC-1, HuPT-3, BXPC-3, MIA PaCa-2, SU86.86, and Panc0403. The CCK-8 assay data indicated that AsPC-1 and PANC-1 cells displayed the strongest resistance to gemcitabine, whereas SU86.86 and BXPC-3 cells showed the highest sensitivity to gemcitabine (Fig. 2B). Interestingly, PC cell lines with higher OTUB1 expression were more resistant to gemcitabine (Fig. 2C), indicating that OTUB1 may protect PC cells from the cytotoxic effects of gemcitabine. Finally, we ascertained whether increased OTUB1 expression led to gemcitabine resistance in PC cells. Our data indicated that the expression level of OTUB1 in a GR cell model was higher than that in the corresponding parental cells (Fig. 2D–F). As shown in Fig. 2G, OTUB1 knockdown reduced gemcitabine resistance and decreased the IC_50_ value in PANC-1/GR cells. Following gemcitabine treatment, we observed a decrease in colony counts and an increase in apoptosis rates in PANC-1/GR cells with OTUB-knockdown (Fig. 2H, I).

To further investigate the functional contribution of OTUB1 in gemcitabine sensitivity, we constructed the OTUB1-overexpressing SU86.86 and BXPC-3 cells using lentivirus overexpression vectors, and used OTUB1-specific shRNA to knock down OTUB1 in AsPC-1 and PANC-1 cells (Supplementary Fig. 2A, B). OTUB1-knockdown cells displayed remarkable attenuation of cell viability with a concurrent increase in the average cell apoptosis rate upon gemcitabine administration, whereas overexpression of OTUB1 in PC cells increased cell viability and enhanced resistance to gemcitabine-induced apoptosis (Fig. 2J, K; Supplementary Fig. 2C, D). Consistently, remarkably higher levels of pro-apoptotic indicators, such as cleaved PARP and caspase 3, were observed in OTUB1-knockdown cells after gemcitabine treatment, whereas OTUB1-overexpressing cells showed decreased expression of these apoptosis factors (Fig. 2L and Fig. S2E). Furthermore, in OTUB1-knockdown cells, we found a decrease in colony counts after gemcitabine treatment, whereas in OTUB1-overexpressing cells, the opposite tendency was seen (Fig. 2M and Fig. S2F). These findings demonstrate that the effect of gemcitabine on PC cells is hindered by OTUB1, thus providing PC cells with a survival advantage even under gemcitabine therapy.

### OTUB1 impedes the response of PC to gemcitabine in vivo

**Fig. 3:**
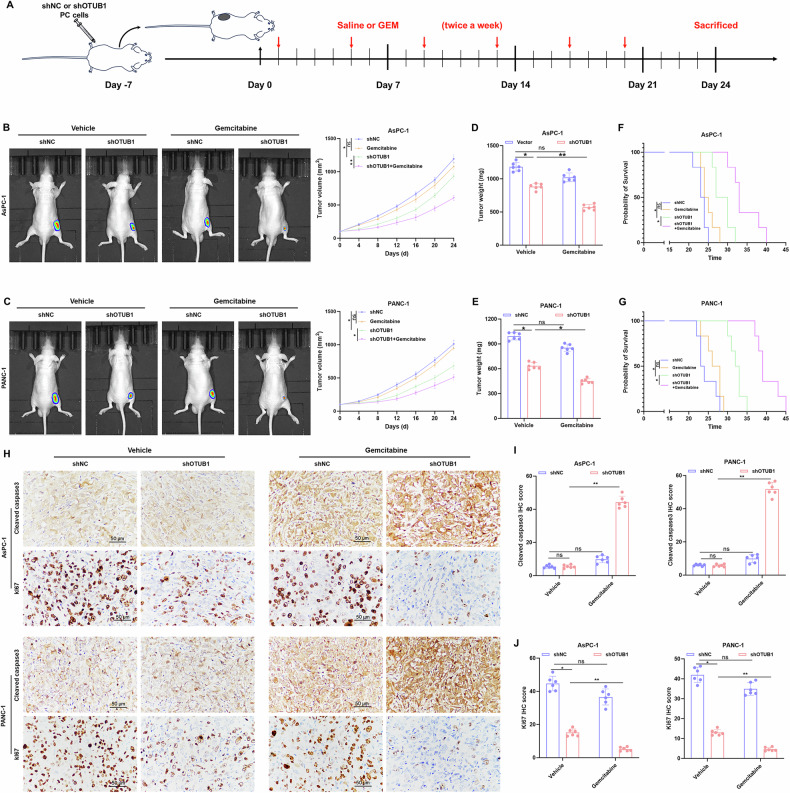
OTUB1 impedes the gemcitabine response to PC cells in vivo. **A** Schematic diagram of the generation and treatment of PC in CDXs. **B**, **C** Images of the tumor tissues (left) and the tumor growth curve (right) of the shOTUB1 group compared to the control (shNC group), with or without gemcitabine treatment. *n* = 6, *p < 0.05, **p < 0.01. **D**, **E** Comparison of tumor weights of the shOTUB1 group compared to the control (shNC group), with or without gemcitabine treatment. *n* = 6, *p < 0.05, **p < 0.01. **F**, **G** Kaplan–Meier analysis of OTUB1 deficiency CDXs treated with or without gemcitabine treatment. *n* = 6-10, *p < 0.05. **H** Representative images of Clevead-caspase-3 and Ki-67 staining of the tumor tissues from the indicated CDXs. Scale bar, 50 μm. **I**, **J** Quantification results of Clevead-caspase-3 and Ki-67 in the indicated groups. *n* = 6, *p < 0.05, **p < 0.01.

Mice were subcutaneously implanted with OTUB1-deficient PC cells to verify the effect of OTUB1 on gemcitabine resistance in vivo. The mice were administered gemcitabine for a specific duration after tumor formation (Fig. 3A). We observed that gemcitabine treatment alone did not have an obvious impact on tumor growth, whereas the tumor growth was slightly slower in the OTUB1-deficient group without gemcitabine treatment; the weight and volume of the tumor were considerably attenuated in the OTUB1-deficient group under gemcitabine treatment (Fig. 3B–E). Compared to that with gemcitabine monotherapy, the OS of CDXs was significantly prolonged in the OTUB1-deficient group combined with gemcitabine treatment (Fig. 3F, G). Furthermore, the data showed weaker staining for Ki-67 (a proliferative indicator) and stronger staining for cleaved caspase 3 (an apoptotic marker) in tumor tissues derived from the OTUB1-deficient group treated with gemcitabine (Fig. 3H–J). Taken together, the results suggest that the abundance of OTUB1 might result in poor response to gemcitabine therapy, and suppression of OTUB1 could enhance the efficacy of therapy.

### OTUB1 confers resistance to gemcitabine by increasing pyrimidine metabolism

**Fig. 4:**
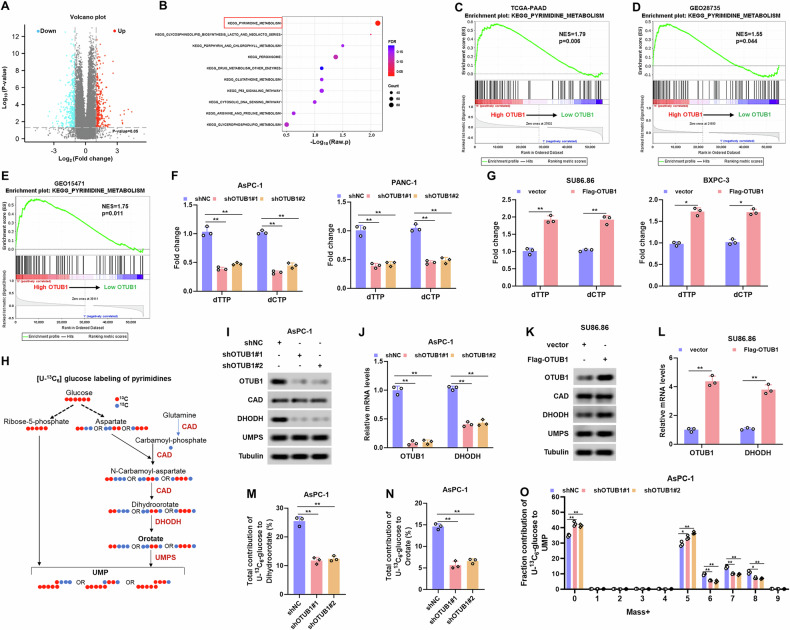
OTUB1 deficiency impaired pyrimidine de novo synthesis via downregulation of DHODH. **A** Volcano plots showing differentially expressed genes in high and low-OTUB1-expressing PC patients from the TCGA-PAAD dataset. |Log_2_FC | >1, *p* value < 0.05. **B** KEGG pathway analysis of OTUB1-associated differentially expressed genes using the TCGA-PAAD dataset. **C**–**E** An enrichment analysis of gene sets (GSEA) available from the TCGA-PAAD, GEO28735, and GEO15471 datasets revealed that OTUB1 expression is positively correlated with pyrimidine metabolism. **F**, **G** Fold changes in deoxycytidine triphosphate (dCTP) and deoxythymidine triphosphate (dTTP) levels from the indicated cells were monitored using a fluorescence-based assay. *p < 0.05, **p < 0.01. **H** Schematic of [U-^13^C^]^ glucose labeling of pyrimidines. **I–L** The mRNA and protein levels of OTUB1, CAD, DHODH, and UMPS in OTUB1-knockdown (**I**, **J**) or -overexpression PC cell (**K**, **L**) were detected by qRT-PCR and western blot assay, respectively. Tubulin was used as the internal standard. **p < 0.01. **M**, **N** Incorporation of carbon atoms from [U-^13^C] glucose into dihydroorotate (**M**) or orotate (**N**) in control and OTUB1 silencing PC cells. **p < 0.01. **O** Massisotopomer distribution (MID) of UMP ^(^uridine monophosphate) from [U-^13^C] glucose in the indicated cells. *p < 0.05, **p < 0.01.

To explore how OTUB1 modulates chemoresistance, we performed a GSEA in TCGA-PAAD database to analyze the possible relationship between a variety of signaling pathways and OTUB1. Kyoto Encyclopedia of Genes and Genomes (KEGG) enrichment analysis revealed a strong correlation between OTUB1 knockdown and the pyrimidine metabolism pathway in TCGA-PAAD database (Fig. 4A, B). Based on GSEA analysis, the pyrimidine metabolic pathway was determined to be a key enrichment pathway in PC tissues with high levels of OTUB1 from the TCGA-PAAD database (Fig. 4C). Simultaneously, data from GEO28735, and GEO15741 supported that OTUB1 is strongly linked to pyrimidine metabolic profiles (Fig. 4D, E). Next, using a sensitive fluorescence-based assay, we found that OTUB1 silencing significantly reduced deoxycytidine triphosphate (dCTP) and deoxythymidine triphosphate (dTTP) levels in PC cells, whereas OTUB1 overexpression increased the levels (Fig. 4F, G). Eventually, we altered the expression of OTUB1 in PC cell lines to detect changes in several critical enzymes involved in the de novo synthesis of pyrimidines, including UMPS, DHODH, and CAD (Fig. 4H). OTUB1 silencing led to a decrease in the protein and mRNA levels of DHODH, whereas OTUB1 overexpression significantly upregulated the expression levels of DHODH in PC cells (Fig. 4I–L and Fig. S3A–D). Nevertheless, UMPS and CAD expression levels remained unchanged in PC cells after OTUB1 knockdown (Fig. 4I–L and Fig. S3A–C).

For further insight, we evaluated the de novo pyrimidine synthesis in AsPC-1 cells in which OTUB1 was silenced by applying uniformly ^13^C-labeled glucose (U-^13^C_6_-glucose), followed by LC-MS/MS analysis. Isotope metabolite tracing showed that OTUB1 silencing resulted in a remarkable reduction in the production of orotate and downstream 5’-monophosphorylated orotidine (Fig. 4M, N), which are critical components of the pyrimidine de novo synthesis pathway. Meanwhile, OTUB1 knockdown caused changes in the UMP distribution (m + 5 to m + 8; Fig. 4O). Overall, the data indicate that OTUB1 suppression could effectively suppress de novo pyrimidine synthesis from glucose in PC cells.

### Impact of OTUB1 on gemcitabine resistance of PC cells relies on its regulation of DHODH

**Fig. 5:**
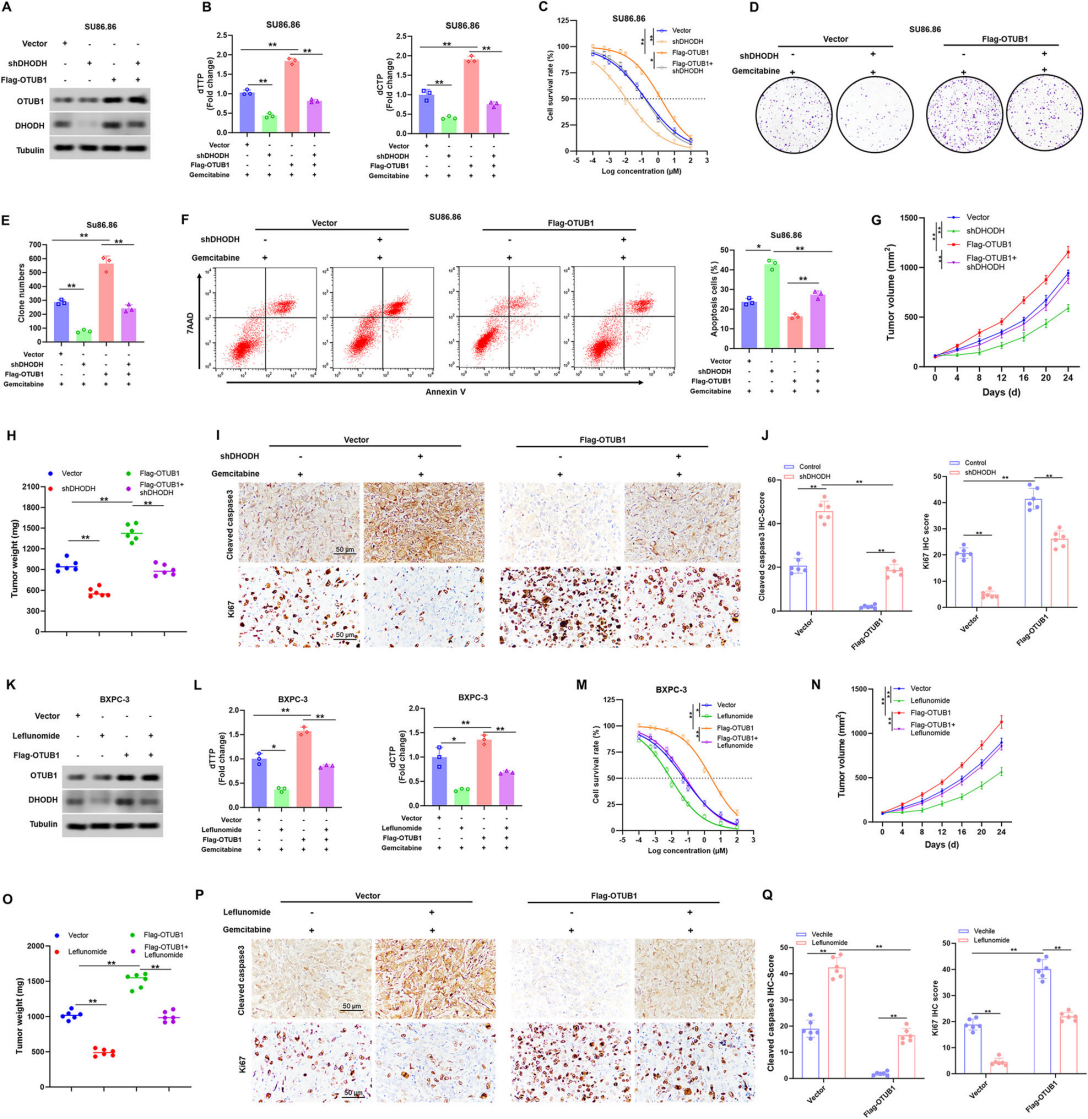
Inhibition of DHODH-mediated pyrimidine synthesis impaired OTUB1-induced gemcitabine resistance in PC. **A** Western blot analysis showing OTUB1 and DHODH expression in the indicated cells upon treatment with gemcitabine. Tubulin was used as the internal standard. **B** Fold changes in dCTP and dTTP levels from the indicated cells were monitored using a fluorescence-based assay. **p < 0.01. **C** Sensitivity to gemcitabine in the indicated cells was detected by CCK-8 assay. *p < 0.05, **p < 0.01. **D**, **E** Re*p*resentative images (**D**) and quantitation (**E**) of colony formation assays were displayed in the indicated cells. *p < 0.05, **p < 0.01. **F** Determination (left) and quantitation (right) of the apoptosis rate in the indicated cells upon treatment with gemcitabine by Flow cytometry. **p < 0.01, ***p < 0.001. **G** Inhibitory effect on tumor growth of treatments with gemcitabine in the indicated CDX models. n = 6, **p < 0.01. **H** The tumor weight in the indicated CDX models upon treatment with gemcitabine. *n* = 6, **p < 0.01. **I**, **J** Representative images and **I** quantitation **J** of Cleaved-caspase 3 and Ki-67 staining in the indicated CDX models upon treatment with gemcitabine. **p < 0.01. Scale bar, 50 μm. **K** Western blot analysis showing OTUB1 and DHODH expression in the indicated cells upon treatment with gemcitabine alone or in a combination of leflunomide. Tubulin was used as the internal standard. **L** Fold changes in dCTP and dTTP levels from the indicated cells upon treatment with gemcitabine alone or in a combination of leflunomide, as determined by fluorescence-based assay. **p < 0.01. **M** Sensitivity to gemcitabine in the indicated cells upon treatment with gemcitabine alone or in a combination of leflunomide were detected by CCK-8 assay. *p < 0.05, **p < 0.01. **N** Inhibitory effect on tumor growth in the indicated CDX models upon treatment with gemcitabine alone or in a combination of leflunomide. **n** = 6, **p < 0.01. **O** The tumor weight in the indicated CDX models upon treatment with gemcitabine alone or in a combination of leflunomide. n = 6, **p < 0.01. **P**, **Q** Representative images and (**I**) quantitation (**J**) of Cleaved-caspase 3 and Ki-67 staining in the indicated CDX models upon treatment with gemcitabine alone or in a combination of leflunomide. **p < 0.01. Scale bar, 50 μm.

DHODH inhibition overcomes gemcitabine resistance in PC cells. Therefore, we investigated whether DHODH could play a critical role in OTUB1-mediated gemcitabine resistance. Here, we found that the OTUB1-induced upregulation of dCTP and dTTP levels was significantly rescued by the knockdown of DHODH (Fig. 5A, B). Under gemcitabine treatment conditions, the increased survival of PC cells, caused by ectopic OTUB1 expression, was abrogated by DHODH silencing (Fig. 5C–F and Fig. S4A). Furthermore, in vivo mouse models showed that xenografts generated from OTUB1-overexpressing PC cells had the smallest tumor weight and volume after DHODH knockdown (Fig. 5G, H). At the same time, rescue experiments showed that knockdown of DHODH expression significantly decreased the expression of proliferative markers and increased the expression of apoptotic markers in tumor tissues from high-OTUB1-expressing mouse tumoroids (Fig. 5I, J).

To further determine whether OTUB1-mediated PC chemoresistance depends on DHODH-mediated pyrimidine metabolism, we treated OTUB1-overexpressing PC cells with or without leflunomide, a DHODH inhibitor. As expected, treatment with leflunomide significantly diminished the OTUB1-induced upregulation of DHODH and reduced dCTP and dTTP levels in OTUB1-overexpressing PC cells (Fig. 5K, L). The cell viability assay using OTUB1-overexpressing PC cells showed a significant reduction in cell survival following treatment with leflunomide and gemcitabine compared to those using cells treated with gemcitabine alone (Fig. 5M and Fig. S4B, C). Furthermore, the number of apoptotic OTUB1-overexpressing PC cells treated with the combination drug was remarkably higher than that of PC cells treated with gemcitabine alone (Fig. S4D, E). Accordingly, the combined treatment with gemcitabine and leflunomide led to higher levels of apoptotic markers in OTUB1-overexpressing PC cells than in the control group (Fig. S4F). Moreover, compared to gemcitabine alone, combination drug treatment weakened the effect of OTUB1 overexpression on gemcitabine resistance in in vivo mouse models (Fig. 5N–Q). Collectively, the findings suggested that OTUB1 confers gemcitabine resistance in PC by modulating DHODH-mediated pyrimidine synthesis.

### OTUB1 up-regulates DHODH expression via interaction with DDX3X

**Fig. 6:**
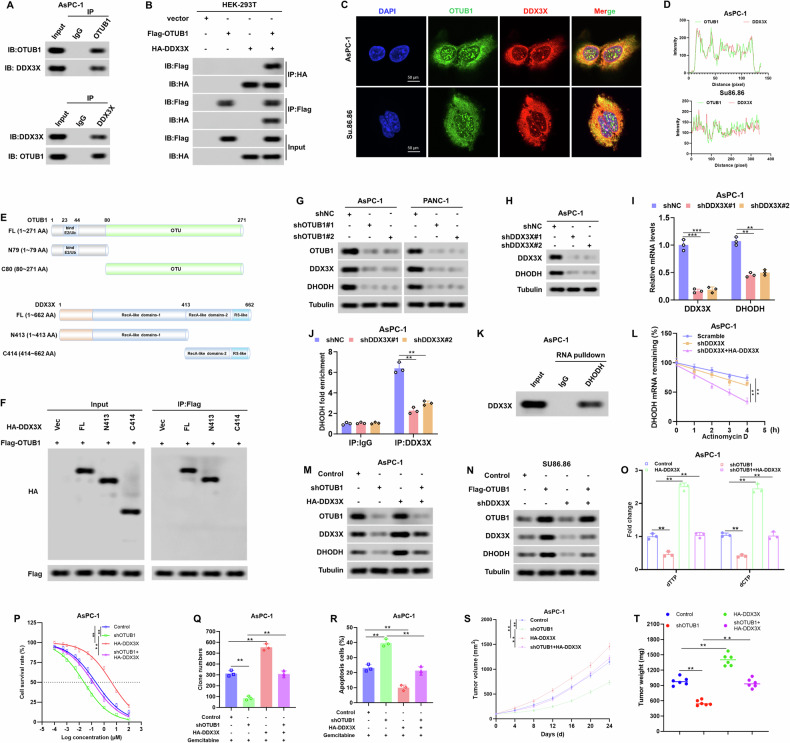
OTUB1 regulates DHODH expression by interacting with DDX3X. **A** AsPC-1 cells were subjected to co-IP assay using either a specific antibody for OTUB1, DDX3X or IgG control, followed by Western blot assay. **B** The HEK-293T cells were transfected with Flag-OTUB1 and HA-DDX3X construct. Total cell lysates were subjected to IP-western blot assay. **C**, **D** Immunofluorescence images (**C**) and quantitation (**D**) demonstrating colocalization of OTUB1 and DDX3X in AsPC-1 and Su86.86 cells. Scale bar, 50 μm. **E** A schematic diagram of OTUB1 (upper panel) and DDX3X (lower panel) domain structure. **F** The HEK-293T cells were transfected with Flag-OTUB1 and HA-DDX3X (FL) or an indicated mutant construct. Total cell lysates were subjected to an IP-western blot assay. **G** Western blot analysis showing OTUB1, DDX3X, and DHODH expression in the indicated cells. Tubulin was used as the internal standard. **H**, **I** The mRNA and protein levels of DDX3X and DHODH in DDX3X-knockdown AsPC-1 cells were detected by qRT-PCR and Western blot assay, respectively. Tubulin was used as the internal standard. **p < 0.01, ***p < 0.001. **J** RIP assays for qRT-PCR quantification of DHODH binding to endogenous DDX3X in AsPC-1 cells. IgG served as a control. **p < 0.01. **K** Exogenous RNA pulldown assays validate the specific interaction between DHODH and DDX3X in AsPC-1 cells. **L** Quantitation of the half-life of DHODH over time following treatment with actinomycin D (10 mg/mL) in the indicated cells. **p < 0.01. **M**, **N** Western blot analysis showing OTUB1, DDX3X and DHODH expression in the indicated cells. Tubulin was used as the internal standard. **O** Fold changes in dCTP and dTTP levels from the indicated cells were monitored using a fluorescence-based assay. **p < 0.01. **P** Sensitivity to gemcitabine in the indicated cells were detected by CCK-8 assay. *p < 0.05, **p < 0.01. **Q** Quantitation of colony formation assays was displayed in the indicated cells. *p < 0.05, **p < 0.01. **R** Quantitation of the apoptosis rate in the indicated cells upon treatment with gemcitabine by Flow cytometry. **p < 0.01. **S** Inhibitory effect on tumor growth of treatments with gemcitabine in the indicated CDX models. *n* = 6, **p < 0.01. **T** The tumor weight in the indicated CDX models upon treatment with gemcitabine. *n* = 6, **p < 0.01.

Using OTUB1 immunoprecipitates from PC cells, we conducted a mass spectrometry assay to investigate the mechanism underlying OTUB1-mediated elevation of DHODH (Fig. S5A, B). Among these, DDX3X was objectively identified as one of the top eight most abundant OTUB1-interacting proteins in our IP-MS data, as ranked by the number of unique peptides detected (Table S3). Importantly, DDX3X was a novel binding protein for OTUB1 in PC cells (Fig. S5C). As an RNA helicase and RNA-binding protein that regulates mRNA stability, DDX3X showed significant co-expression with the OTUB1-regulated gene DHODH in the TCGA-PAAD database (Fig. S5D). And, our results also demonstrate a positive correlation between OTUB1 and DDX3X protein levels in PC cell lines (Fig. S5E). Coupled with OTUB1’s role in regulating protein stability, this correlation suggests their interaction coordinates post-transcriptional and post-translational regulation of pyrimidine synthesis pathways involving DHODH. A series of experiments was performed to validate this hypothesis. Co-IP assay demonstrated an interaction between endogenous DDX3X and OTUB1 in PC cells (Fig. 6A and Fig. S5F), and between exogenous DDX3X and OTUB1 in HEK-293T cells (Fig. 6B). This interaction was further confirmed by the colocalization of DDX3X and OTUB1 according to confocal imaging in PC cells (Fig. 6C, D). Mapping of the binding regions of DDX3X and OTUB1 revealed that OTUB1 interacted with DDX3X through its N-terminal region (aa 1–413), which was predominantly occupied by the recombinase A (RecA)-like structural domain 1 (Fig. 6E, F). Meanwhile, the C-terminal region of OTUB1 (aa 80–271) was necessary for OTUB1 to interact with DDX3X (Fig. S5G). Then, we determined whether OTUB1 modulated DDX3X expression. Notably, in OTUB1-knockdown PC cells, there was a clear decrease in DDX3X protein levels (Fig. 6G), whereas PC cells overexpressing OTUB1 had higher levels of DDX3X protein (Fig. S6A). However, our data demonstrated that the mRNA level of DDX3X was not significantly different after alteration of OTUB1 in PC cells (Fig. S6B). Moreover, high-OTUB1 levels positively correlated with high levels of DDX3X and DHODH in tissues from patients with PC (Fig. S6C–E).

Next, we examined the relevance of DDX3X and DHODH in PC cells. As expected, DHODH expression was reduced in DDX3X-knockdown PC cells at both protein and mRNA levels (Fig. 6H, I). DDX3X, an RNA-binding protein, is involved in RNA splicing and mRNA degradation, and is critical for tumorigenesis and chemoresistance in many cancers, including PC. As shown in Fig. 6J, K, RNA immunoprecipitation and RNA pulldown assays demonstrated that DDX3X directly interacts with DHODH mRNA. Importantly, the knockdown of DDX3X significantly accelerated the decay of DHODH mRNA in PC cells after treatment with actinomycin D to block transcription (Fig. 6L).

To further verify this result, ectopic expression of DDX3X abrogated the effect of OTUB1 deficiency on DHODH downregulation in PC cells (Fig. 6M) and vice versa (Fig. 6N), suggesting that OTUB1-mediated DHODH upregulation is DDX3X dependent. In addition, the results of the fluorescence-based assay revealed that overexpression of DDX3X reversed the OTUB1 deficiency-induced dCTP and dTTP reduction (Fig. 6O) and vice versa (Fig. S6F). Consistently, overexpression of DDX3X significantly abolished the OTUB1 knockdown-mediated gemcitabine sensitivity as per in vivo and in vitro assays (Fig. 6P–T) and overexpression of DDX3X revealed an opposite result (Fig. S6G–K). In addition, we transfected a DHODH overexpression plasmid into OTUB1-knockdown PC cells and examined its effects on these cellular functions (Fig. S7A). The results showed DHODH overexpression significantly reversed the reduction in dCTP and dTTP levels caused by OTUB1-knockdown (Fig. S7B). And, DHODH overexpression abolished OTUB1 knockdown-mediated gemcitabine sensitivity in both in vivo and in vitro assays (Fig. S7C–G). These findings together demonstrate that in PC, the induction of OTUB1-mediated expression of DHODH and reprogramming of pyrimidine metabolism is dependent on DDX3X.

### OTUB1 stabilizes DDX3X protein by reducing its K48-linked polyubiquitination level

**Fig. 7:**
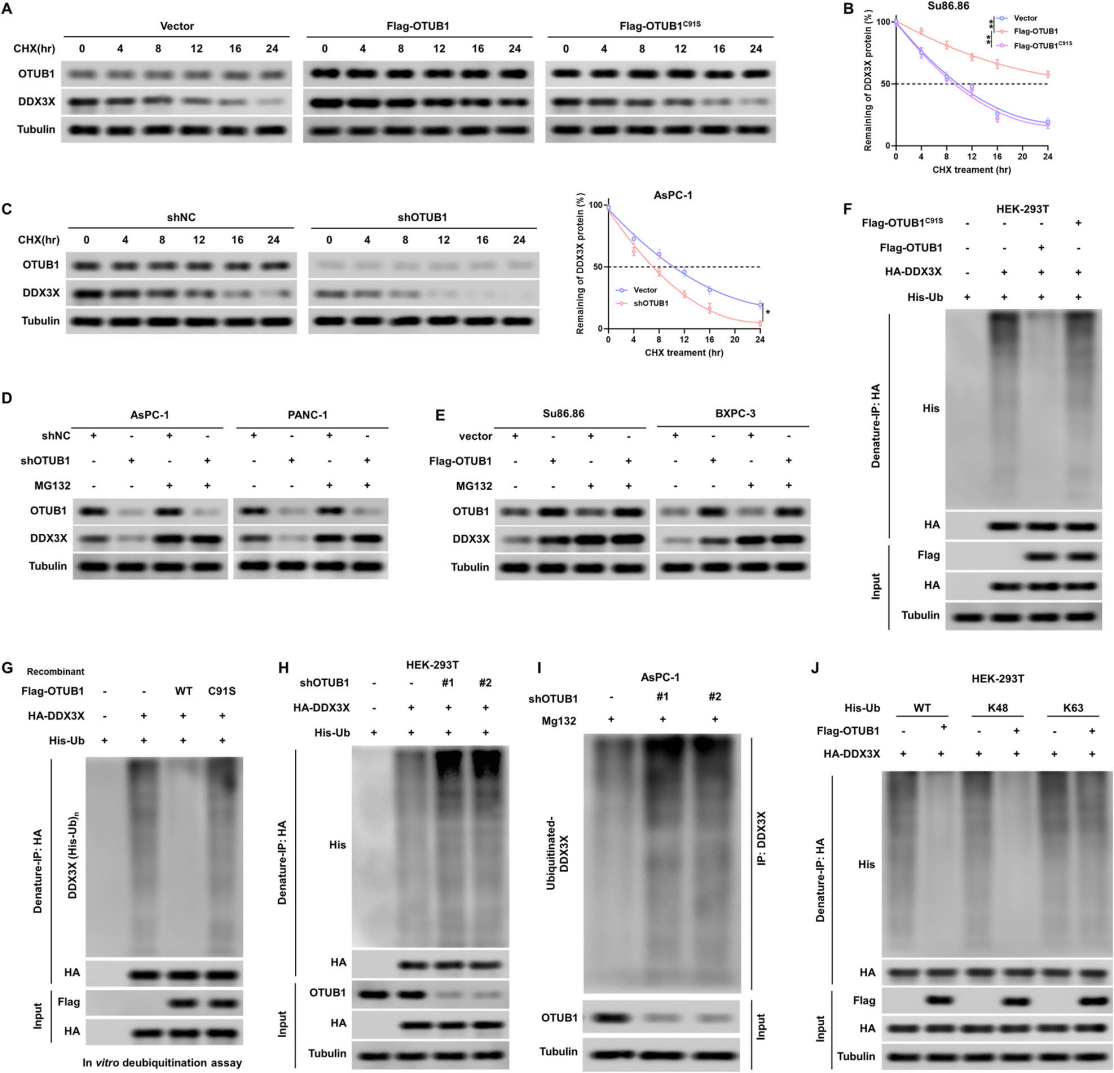
OTUB1 suppressed the ubiquitin-proteasome-mediated degradation of DDX3X. **A**, **B** Representative (**A**) and quantitative (**B**) results of DDX3X protein level in Su86.86 cells stably expressing OTUB1 or OTUB1^C91S^. The cells were treated with cycloheximide (CHX, 50 μg/mL) for the indicated time points and subjected to western blot analysis. **p < 0.01. **C** OTUB1-knockdown AsPC-1 cells were treated with 50 μg/mL CHX for the indicated time intervals (left). The turnover of DDX3X is indicated graphically (right). **p < 0.01. **D**, **E** PC cells stably expressing shOTUB1 (**D**) or Flag-OTUB1 (**E**) were treated with 10 μM MG132. Cells were collected at 6 h and immunoblotted with the antibodies indicated. Tubulin was used as the internal standard. **F** HEK-293T cells were transfected with HA-DDX3X and Flag- Flag-OTUB1 or Flag-OTUB1^C91S^ plasmid. Total cell lysates were subjected to IP-western blot analysis. **G** Ubiquitinated HA-DDX3X expressed in HEK-293T cells was purified by denature-IP and incubated with recombinant Flag-OTUB1 or Flag-OTUB1^C91S^. **H** HEK-293T cells were co-transfected with indicated expressing plasmids upon treated with 20 μM MG132 for 6 h before collection. Ubiquitination of DDX3X was examined by denatured IP-Western blot analysis. **I** Knockdown of OTUB1 altered the ubiquitination of DDX3X. The cells in each group were treated with proteasomal inhibitor MG132. Cell lysates were prepared and subjected to immunoprecipitation with anti-DDX3X antibodies. **J** HEK-293T cells were co-transfected with indicated expressing plasmids upon treated with MG132. Ubiquitination of DDX3X was examined by denature-IP-western blot analysis.

We examined the mechanism by which OTUB1 modulates the expression of DDX3X in PC. Our results showed that OTUB1 dysregulation had no effect on DDX3X mRNA levels in PC cells (Fig. S5H), suggesting that OTUB1 promotes the protein accumulation of DDX3X in a post-transcriptional manner. To test this hypothesis, we first investigated the effect of OTUB1^WT^ or OTUB1^C91S^, a mutant defective in deubiquitination [23], on DDX3X expression in PC cells. As expected, the ectopic expression of OTUB1^WT^ but not OTUB1^C91S^, delayed the protein half-life of DDX3X in the cycloheximide pulse-chase assay (Fig. 7A, B). In contrast, the lack of OTUB1 remarkably shortened the half-life of DDX3X protein in PC cells (Fig. 7C). Furthermore, our data demonstrated that after treatment with the proteasome inhibitor MG132, the DDX3X protein level recovered in OTUB1-overexpressing or -knockdown PC cells (Fig. 7D, E). Moreover, the overexpression of OTUB1^WT^ but not OTUB1^C91S^ significantly removed the ubiquitin chains of DDX3X both in vivo and in vitro (Fig. 7F, G), whereas silencing of OTUB1 enhanced the polyubiquitination level of DHODH (Fig. 7H). And, OTUB1 deficiency significantly increased polyubiquitination of endogenous DDX3X compared to parental cells (Fig. 7I). Finally, ectopic expression of OTUB1 dramatically reduced the level of ubiquitination of the DDX3X K48 linkage while having no impact on the ubiquitination of the K63 linkage (Fig. 7J). Overall, the findings suggest that OTUB1 promotes DDX3X protein stability by mediating DDX3X deubiquitination.

#### Small-molecule inhibitors of OTUB1 sensitized PC to gemcitabine therapy

**Fig. 8:**
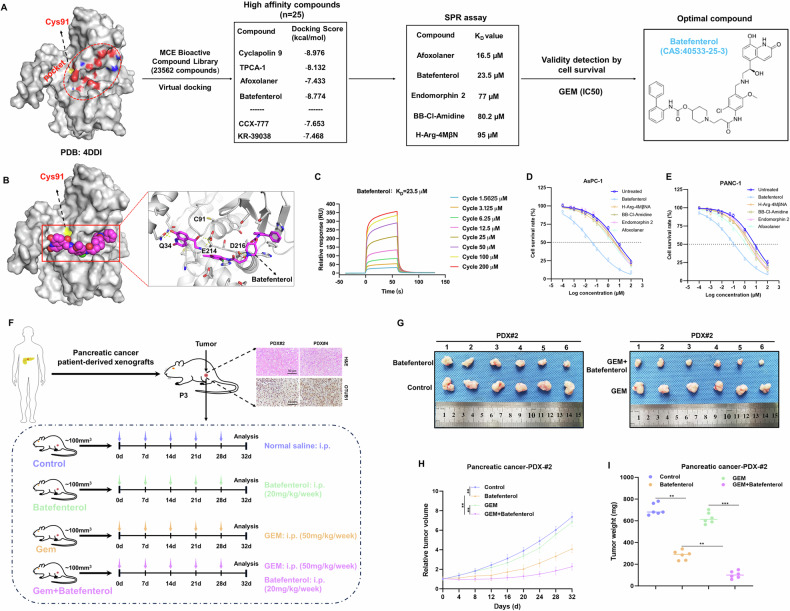
The combination of OTUB1 inhibitor and gemcitabine inhibits tumor growth and improves survival in preclinical models of PC. **A** The flow diagram for OTUB1 inhibitor screening. **B** Computational model and interactions of batefenterol and OTUB1. **C** Kinetic constant (KD) analysis of batefenterol interacting with OTUB1 using surface plasmon resonance (SPR) assay. **D**, **E** IC50 value of Afoxolaner, Batefenterol, Endomorphin 2, BB-Cl-Amidine, and H-Arg-4MβNA in AsPC-1 (**D**) and PANC-1 (**E**) cells upon treatment with gemcitabine were detected by CCK-8 assays, respectively. **F** Schematic diagram of the generation and treatment of PC in PDXs. **G**–**I** The representative images (**G**), tumor growth (**H**), and tumor weight (**I**) in OTUB1^high^ PDXs treated with gemcitabine, batefenterol, or both. n = 6, **p < 0.01, ***p < 0.001.

Recent research revealed that pharmacological suppression of the drivers of cancer enhances the effectiveness of chemotherapy in a wide range of cancer types [24]. To determine this, the active site Cys91 of the OTUB1 protein was identified as an inhibitor-binding pocket for small-molecule compound screening (Fig. 8A). From the MCE bioactive chemical library, including 23,562 compounds using virtual screening, 25 possible compounds were chosen based on their molecular weight, skeletal variety, and docking score (Fig. 8A). Subsequently, we further chose 5 compounds (Afoxolaner, Batefenterol, Endomorphin 2, BB-Cl-Amidine, and H-Arg-4MβNA) using the SPR assays. The results of the SPR assay revealed that batefenterol was able to assemble with OTUB1, with a dissociation constant (*K*_d_) of 23.5 μM (Fig. 8B, C, Fig. S8A). Furthermore, batefenterol was identified as the optimal compound, through efficacy assays, in PC cells with high-OTUB1 levels. We found that batefenterol treatment significantly reduced gemcitabine resistance and the IC_50_ value in PC cells (Fig. 8D, E). Moreover, our results indicated that batefenterol inhibited the DDX3X-DHODH axis in PC cells (Fig. S8B).

We assessed the pharmacological synergy/antagonism of gemcitabine and batefenterol using PDX models. Firstly, to clinically validate the effect of targeting OTUB1 chemically on the gemcitabine response in PC, a subcutaneous PDX model was established based on the high expression OTUB1 in PC tumor tissues (Fig. S8C). Then, we evaluated the therapeutic effect of the combination of batefenterol and gemcitabine in PDX models with high-OTUB1 levels (Fig. 8F). Our data indicate that there was a little or modest effect of gemcitabine monotherapy on the development of high-OTUB1-expressing PDX tumors; co-administration of gemcitabine and batefenterol resulted in significant tumor regression (Fig. 8G–I, Fig. S8D, E). Finally, compared to gemcitabine monotherapy, the OS of PDXs was significantly prolonged in the group following co-administration of gemcitabine and batefenterol (Fig. S8F, G). Collectively, these results demonstrate that batefenterol was identified as a potent OTUB1 inhibitor and gemcitabine sensitizer, and combination therapy with batefenterol and gemcitabine may improve the therapeutic efficacy in PC.

## Discussion

Failure of gemcitabine-based chemotherapy is a significant challenge in the successful treatment of PC. Uncovering the mechanisms by which resistance to gemcitabine can develop is important for overcoming this limitation and for developing effective therapies [25]. Although the exact mechanisms underlying this phenomenon are still unknown, data point to the possibility that ubiquitin-specific peptidases (USPs) are important modulators of cancer chemoresistance [26]. Here, we revealed that OTUB1-mediated synthesis of pyrimidine is an early event that increases resistance to gemcitabine in PC cells both in vivo and in vitro. Additionally, the effect of OTUB1 on the response of PC to gemcitabine, found in our study, indicated that OTUB1 may be a predictor of chemotherapeutic efficacy and a potential therapeutic target for combating gemcitabine resistance in PC.

Metabolic reprogramming is a hallmark of many cancers, wherein pyrimidine biosynthesis is boosted by the activation of the pentose phosphate pathway, leading to an increase in the formation of deoxycytidine triphosphate (dCTP), a competitor of gemcitabine that neutralizes its therapeutic impact [27]. The regulation of pyrimidine metabolism is multistep and complex, and numerous upstream regulatory variables related to pyrimidine metabolism have been suggested in earlier research [28]. For instance, by controlling pyrimidine metabolism, targeted suppression of UBE2T was shown to improve gemcitabine efficacy in PC [29]. Previous studies have demonstrated that progression and poor prognosis of hepatocellular carcinoma are closely linked to the increasing pyrimidine metabolism [30, 31]. In this study, we demonstrated that OTUB1 knockdown alone significantly sensitized murine tumors and PC cells to gemcitabine and inhibited de novo nucleotide biosynthesis in PC cells. Furthermore, our study predicted that blocking the deubiquitinating activity of OTUB1 could functionally mirror OTUB1 knockdown and sensitize refractory PDAC to gemcitabine therapy. In the current study, we reported the high-affinity and specific inhibition of OTUB1 by a compound (batefenterol) both in vitro and in human cells. Compared to gemcitabine monotherapy, the use of batefenterol combined with gemcitabine improved the effectiveness of chemotherapy both in vivo and in vitro. The results collectively suggested that OTUB1 leads to gemcitabine resistance in PC and that use of batefenterol may be a prospective strategy for the treatment of patients with PC. However, optimization of the chemical structure of batefenterol, characterization of its toxicological and pharmacokinetic profiles, and conduct of subsequent clinical trials would be essential to realize the clinical application of this new combination therapy in the future. In addition, batefenterol was identified as a novel OTUB1 inhibitor through our original high-throughput screening. We confirmed its on-target activity by consistent suppression of OTUB1-regulated effector molecules and functional recapitulation of OTUB1-knockdown phenotypes in PC cells. While these findings establish batefenterol as an effective OTUB1 modulator in our model, we acknowledge its known β2-adrenergic receptor agonism and potential activity against other unexamined DUBs as possible contributors to the observed phenotypes.

The pathway for de novo pyrimidine synthesis is tightly regulated by various enzymes [32]. DHODH is a rate-limiting enzyme that catalyzes the fourth reaction of pyrimidine biosynthesis in the mitochondria [33]. The upregulation of DHODH has been reported to augment the synthesis of pyrimidines and promote the progression of various cancers [31, 34]. Aberrant alterations in DHODH by many oncogenes have been demonstrated to enhance the synthesis of pyrimidines in many cancers. For instance, the transcription factor SOX2 has been shown to enhance de novo pyrimidine synthesis to promote OSCC progression by upregulating DHODH [35]. Knockdown of aryl hydrocarbon receptor (AHR) decreased DHODH expression, which regulates metabolic pathways essential for glioblastoma cell growth [36]. Previous studies have shown that leflunomide, a DHODH inhibitor, exhibits anti-proliferative activity as a single agent in PC cells and synergistically inhibits their growth in combination with gemcitabine [37]. In the present study, we also demonstrated that a decrease in DHODH expression induced by OTUB1 knockdown changed the process of de novo pyrimidine biosynthesis. Furthermore, the combination of gemcitabine with a pharmacological inhibitor of DHODH (leflunomide) significantly potentiated the efficacy of gemcitabine in high-OTUB1-expressing murine tumoroids. These data provided the basis for exploring other regulatory factors involved in the pyrimidine synthesis pathway. While systematic evaluation of leflunomide-gemcitabine toxicity and pharmacokinetic compatibility remains beyond the scope of this study, several lines of evidence support its translational feasibility: (1) the established clinical safety profile of leflunomide in chronic autoimmune disorders; (2) the mechanistic complementarity between pyrimidine synthesis inhibition (by leflunomide) and nucleoside analog chemotherapy (gemcitabine); and (3) the significant tumor suppression coupled with preserved animal viability observed in our study. Further in vivo safety assessments will strengthen the clinical translational potential of this combination and will be studied in future.

DDX3X is a member of the Asp-Glu-Ala-Asp (DEAD)-box helicase family, and is implicated in many aspects of RNA metabolism [38]. Aberrant DDX3X expression is a common phenomenon in various tumors and is correlated with the development and progression of human cancers [39, 40]. Here, we found that OTUB1 directly interacted with DDX3X and suppressed its degradation and ubiquitination, thereby stabilizing the expression of DDX3X in PC cells. DDX3X is a multifunctional RNA helicase that participates in a wide range of biological activities including RNA splicing, mRNA stability, and ribosome synthesis [21, 41]. DDX3X, as an RNA helicase and co-regulator of many variables, may regulate multiple mRNA targets that promote cancer development. In breast cancer cells, DDX3X directly interacts with the KLF4 mRNA and regulates its splicing [42]. DDX3X has been found to promote Zc3h12a in mouse embryonic fibroblasts by stabilizing Zc3h12a mRNA following IL-17 stimulation [43]. According to a previous study, kindlin-2 promotes PC development by increasing c-Myc translation through its interaction with DDX3× [44]. And, DDX3X binds to and stabilizes Rab27a mRNA in hepatocellular carcinoma [45]. Specifically, our study showed that DDX3X directly binds to DHODH mRNA and enhances its expression by inhibiting mRNA degradation in PC cells. More importantly, knockdown or inhibition of either DDX3X or DHODH significantly reverses the phenotypic changes induced by OTUB1 overexpression in PC cells. This body of evidence strongly supports the conclusion that the OTUB1-DDX3X axis promotes gemcitabine chemoresistance in pancreatic cancer by stabilizing DHODH mRNA. While DDX3X may regulate a broader spectrum of mRNA targets in PC cells, our functional and mechanistic data robustly indicate that DHODH is the primary and functionally critical target downstream of the OTUB1-DDXX3 axis in regulating pyrimidine synthesis and gemcitabine resistance.

## Conclusions

**Fig. 9:**
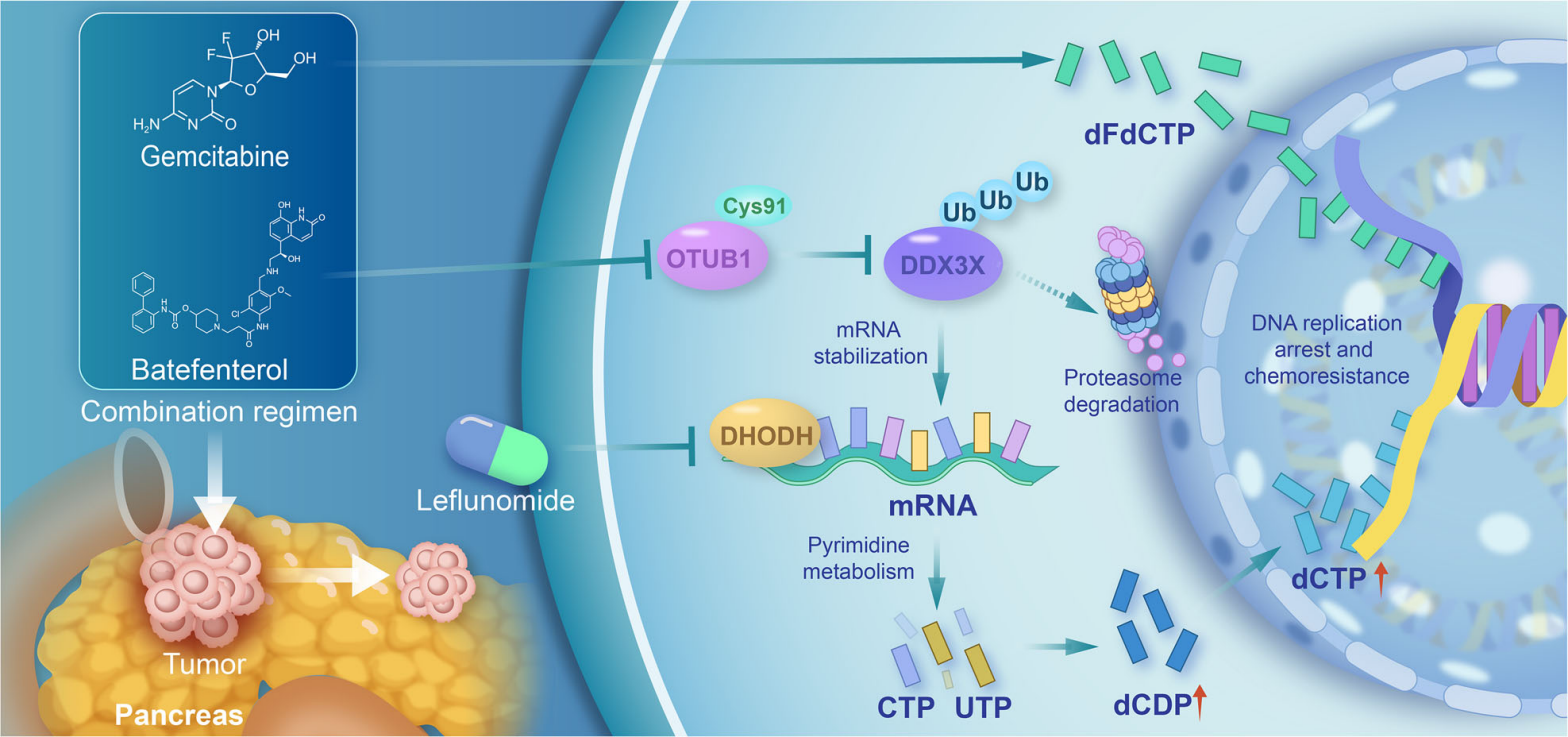
Diagram depicts that targeting OTUB1 sensitizes pancreatic cancer to gemcitabine chemotherapy. OTUB1 interacts and stabilizes DDX3X to enhance DHODH-mediated de novo pyrimidine synthesis, thereby upregulating dCDP and dCTP for efficient DNA replication, reducing gemcitabine-induced replication stress, and conferring gemcitabine resistance in PC.

In summary, the therapeutic significance of OTUB1 in providing chemoresistance to patients with PC was validated; high-OTUB1-expressing murine and human tumors demonstrated noticeably lower gemcitabine sensitivity than the low-OTUB1-expressing group. Our findings demonstrated the pathogenic function of the OTUB1-DDX3X-DHODH axis in PC gemcitabine resistance (Fig. 9) and implied that gemcitabine resistance in PC may be addressed by targeting and inhibiting this axis. These findings could not only expand our knowledge of metabolic adaptation-mediated chemoresistance in cancer but also facilitate the development of a new strategy to improve the gemcitabine efficacy in PC.

## Supplementary information

Supplementary Figures and Figure legends

Table S1

Table S2

Table S3

Uncropped version of all Figures

## Data Availability

All data, analytical methods, and study materials are available from the corresponding author on request.
