# Supplementary material for: The deubiquitylase OTUB1 drives gemcitabine resistance in pancreatic cancer by enhancing pyrimidine metabolism through modulating DHODH mRNA stability
Source: Cell Death Dis. 2025 Oct 6;16(1):697. doi: 10.1038/s41419-025-08001-4 (PMC12501277; doi:10.1038/s41419-025-08001-4)
Supplement: Supplementary file 1 — Supplementary Figures and Figure legends [file 41419_2025_8001_MOESM1_ESM.docx]

**Supplementary Figures and Figure legends**


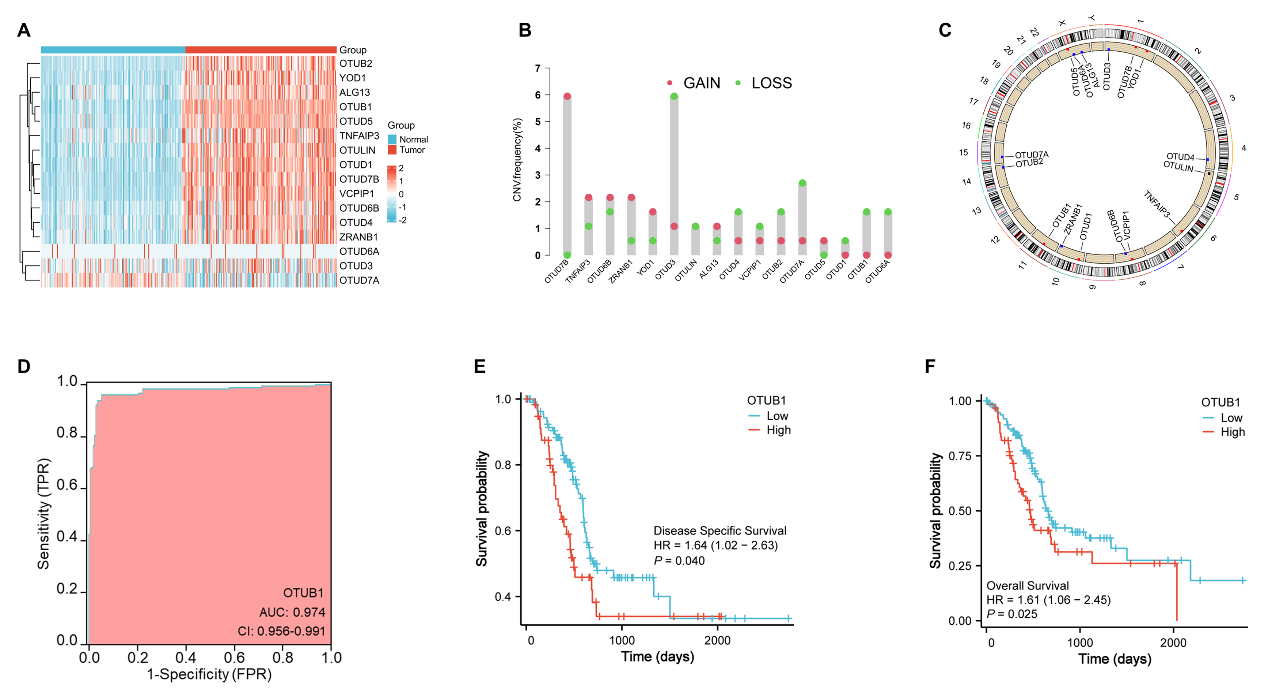


**Figure S1. OTUB1 may serve as a valuable new prognostic factor for the patients with PC. (A)** The heatmap volcano plot of the 16 differentially expressed human OTU domain DUBs in PC tissues and the normal tissue from the TCGA-PAAD dataset and GTEx dataset. Gene expression values are z-transformed. **(B)** The CNV variation frequency of OTU domain DUBs in TCGA-PAAD cohort. The height of the column represented the alteration frequency. The deletion frequency, blue dot; The amplification frequency, red dot. **(C)**The location of CNV alteration of OTU domain DUBs on 23 chromosomes using TCGA-PAAD cohort. **(D)** Diagnostic ROC curves to distinguish PC tissues and normal tissues based on the OTUB1 expression levels. **(E and F)** PC patients with higher OTUB1 expression show poorer clinical prognosis in the TCGA cohort.


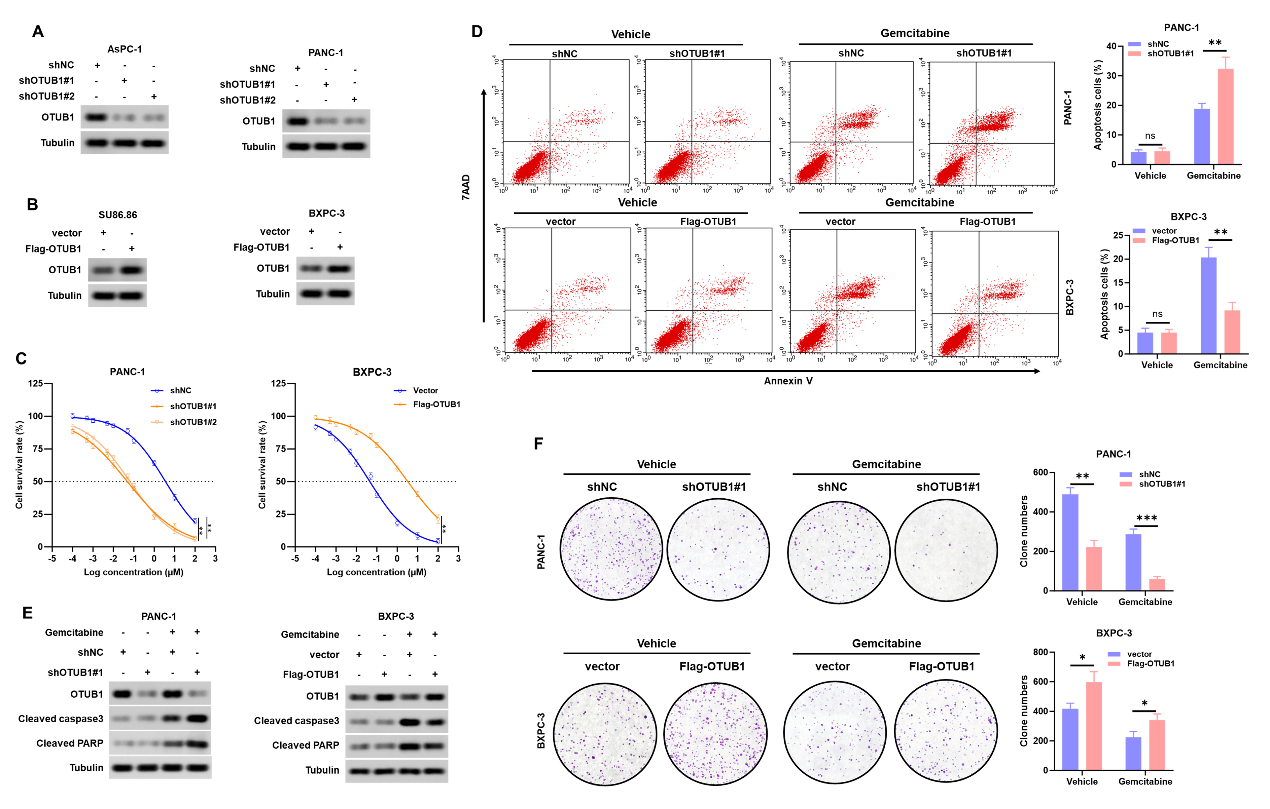


**Figure S2. Knockdown of OTUB1 enhances gemcitabine sensitivity of PC cells.** **(A)** Protein levels of OTUB1 were assessed in AsPC-1 and PANC-1 cells transfected with shNC or shOTUB1 by western blotting assay. Tubulin was used as the internal standard. **(B)** Protein levels of OTUB1 assessed in Su86.86 and BXPC-3 cells transfected with vector or Flag-OTUB1 by western blotting assay. Tubulin was used as the internal standard. **(C)** Sensitivity to gemcitabine in the indicated cells were detected by CCK-8 assay. ^**^*p*< 0.01. **(D)** Determination (left) and quantification (right) of the apoptosis rate in the indicated cells. ^**^*p*< 0.01. **(E)** Protein level of OTUB1, Cleaved-caspase3, and Cleaved-PARP were determined by Western blot assays in the indicated cells after treatments with or without gemcitabine. Tubulin was used as the internal standard. **(F)** Representative images (left) and quantification (right) of colony formation assays in the indicated cells. ^*^*p*< 0.05, ^**^*p*< 0.01, ^***^*p*< 0.01.


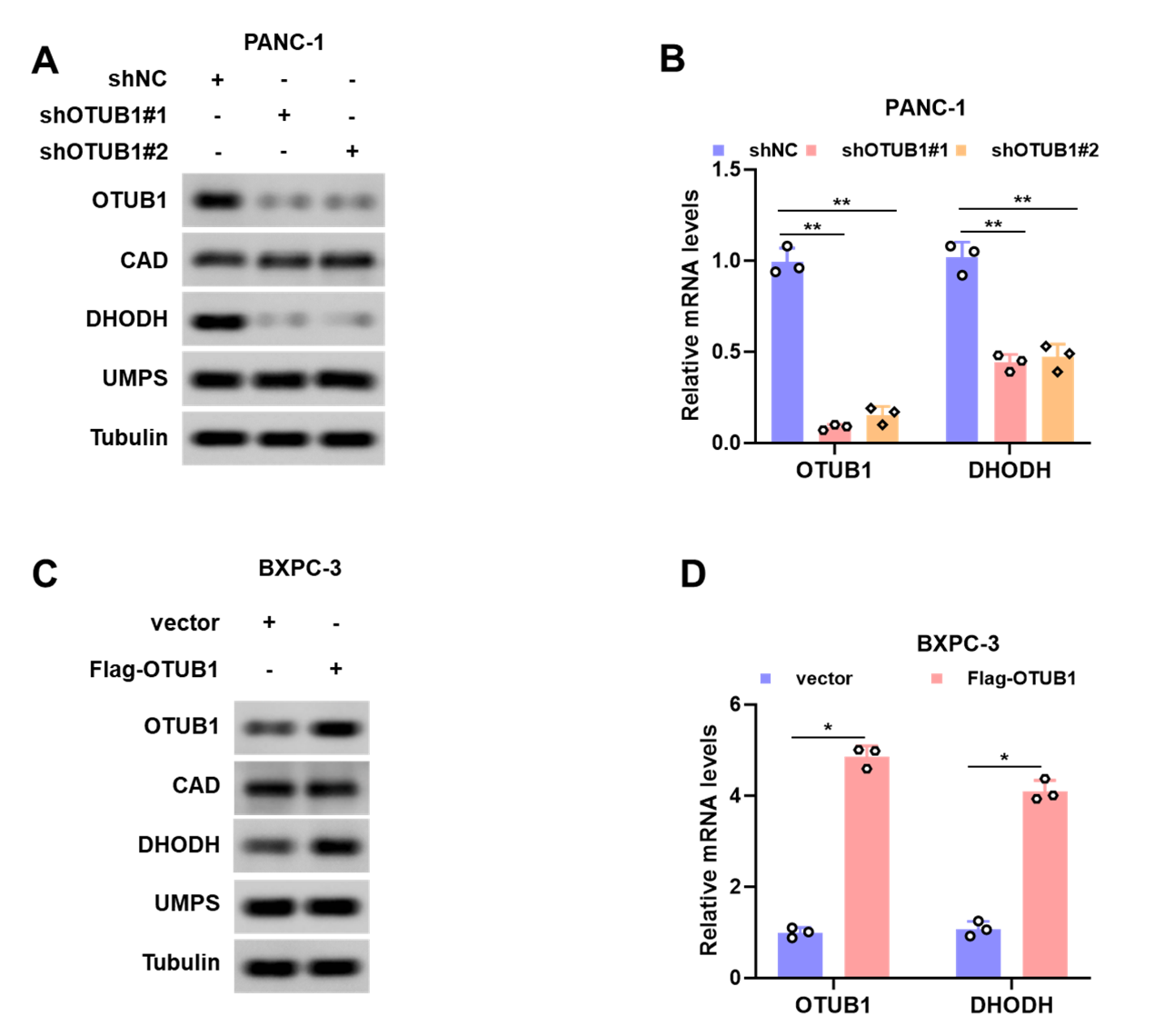


**Figure S3. Silencing of OTUB1 impaired *de novo* pyrimidine synthesis.** **(A and B)** The mRNA and protein levels of OTUB1, CAD, DHODH, and UMPS in OTUB1-knockdown PC cell were detected by qRT-PCR and Western blot assay, respectively. Tubulin was used as the internal standard. Tubulin was used as the internal standard. ^**^*p*< 0.01. **(C and D)** The mRNA and protein levels of OTUB1, CAD, DHODH, and UMPS in OTUB1-overexpression PC cell were detected by qRT-PCR and Western blot assay, respectively. Tubulin was used as the internal standard. Tubulin was used as the internal standard. ^**^*p*< 0.01.


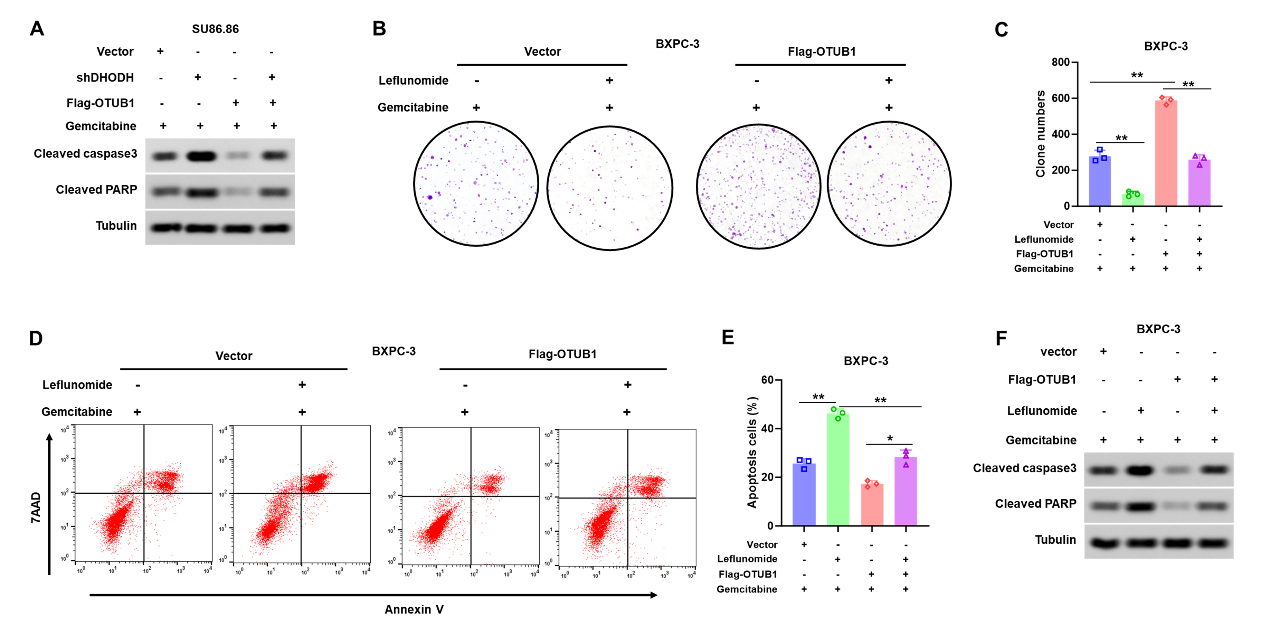


**Figure S4. OTUB1 confers gemcitabine resistance to PC cells in DHODH-dependent manner. (A)** Protein level of Cleaved-caspase3, and Cleaved-PARP were determined by Western blot assays in the indicated cells upon treatments with gemcitabine. Tubulin was used as the internal standard. **(B and C)** Representative images (B) and quantification (C) of colony formation assays in the indicated cells upon treatment with gemcitabine alone or in a combination of leflunomide. ^*^*p*< 0.05, ^**^*p*< 0.01. **(D and E)** Determination (D) and quantification (E) of the apoptosis rate in the indicated cells upon treatment with gemcitabine alone or in a combination of leflunomide as determined by Flow-cytometry. ^**^*p*< 0.01, ^***^*p*< 0.001. **(F)** Protein level of Cleaved-caspase3, and Cleaved-PARP were determined by Western blot assays in the indicated cells upon treatment with gemcitabine alone or in a combination of leflunomide. Tubulin was used as the internal standard.


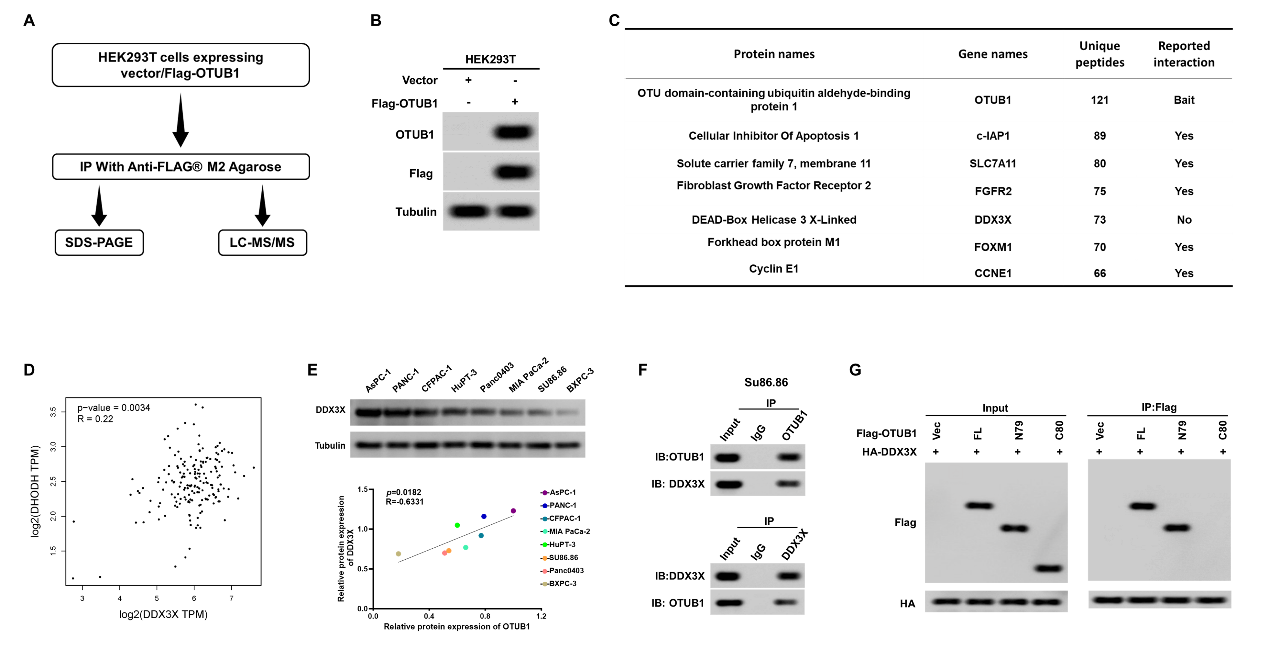


**Figure S5. OTUB1 directly interacts with DDX3X. (A and B)** HEK-293T cells stably expressing empty vector and Flag-OTUB1 were subjected to IP assays using anti-Flag affinity gel beads. **(C)** The top six OTUB1-interacting proteins according to the number of identified unique peptides. **(D)** Pearson correlation analysis of DDX3X and DHODH in TCGA-PAAD dataset. **(E)** Top: Determination of DDX3X protein levels in eight PC cell lines by Western blot assay. Tubulin was used as the internal standard. Bottom: Correlation among the expression of OTUB1 and DDX3X in PC cell lines. **(F)** Su86.86 cells were subjected to co-IP assay using either a speciﬁc antibody for OTUB1, DDX3X or IgG control followed by Western blot assay. **(G)** The HEK-293T cells were transfected with HA-DDX3X and Flag-OTUB1 (FL) or an indicated mutant construct. Total cell lysates were subjected to IP-western blot assay.


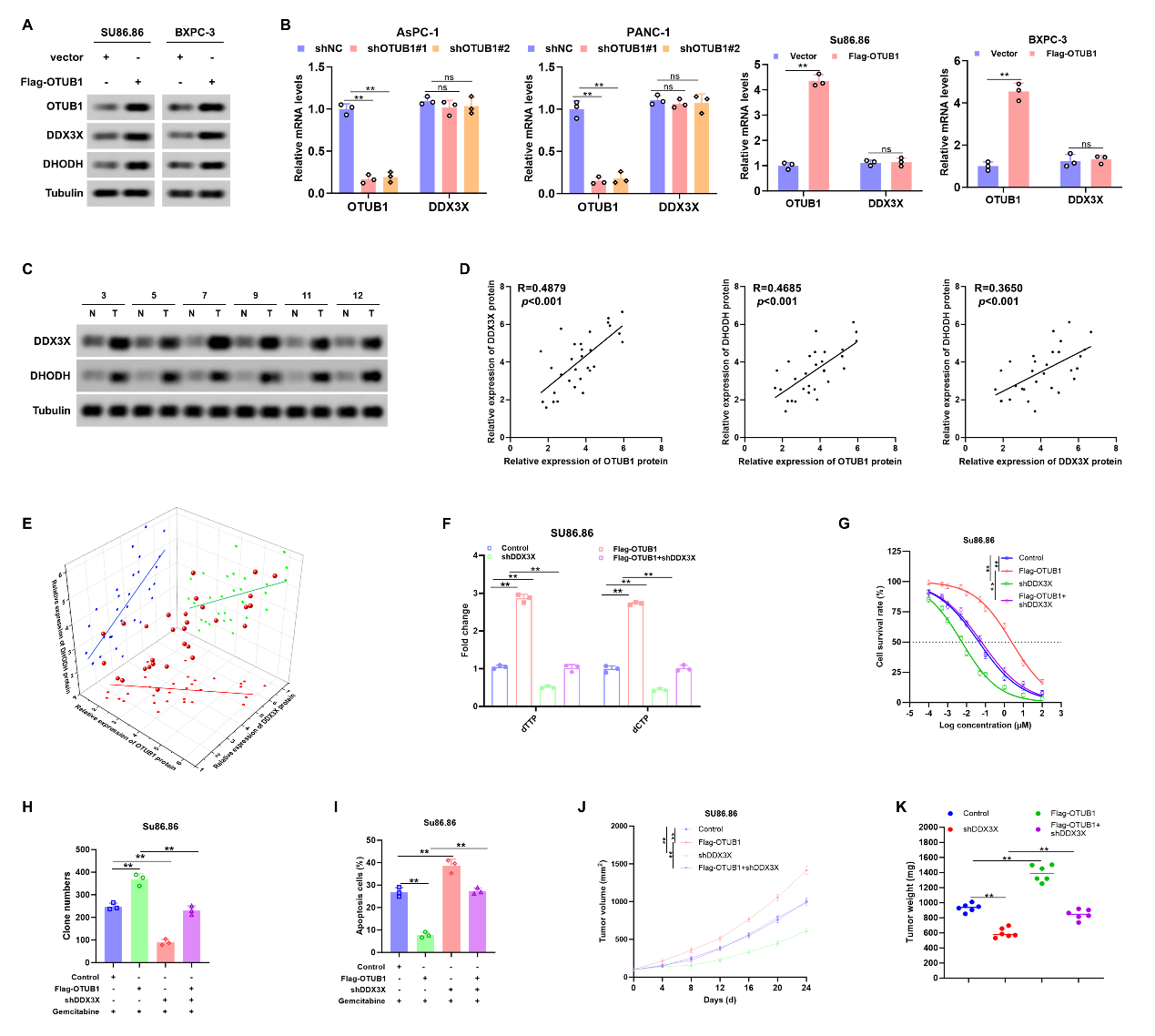


**Figure S6. OTUB1 regulates DHODH expression though DDX3X. (A)** Western blot analysis showing OTUB1, DDX3X, and DHODH expression in the OTUB1-overexpression PC cells. Tubulin was used as the internal standard. **(B)** The mRNA of OTUB1 and DDX3X in the indicated cells were detected by qRT-PCR. ^**^*p*< 0.01. **(C)** Representative western blotting analysis of DDX3X and DHODH protein levels in PC tissues with high OTUB1 expression. Tubulin was used as the internal standard. **(D)** Spearman correlation analysis of OTUB1, DDX3X, and DHODH protein expression in PC tissues, respectively. **(E)** Correlation among the expression of OTUB1, DDX3X, and DHODH in PC tissues. **(F)** Fold changes in dCTP and dTTP levels from the indicated cells were monitored using a fluorescence-based assay. ^**^*p*< 0.01. **(G)** Sensitivity to gemcitabine in the indicated cells were detected by CCK-8 assay. ^*^*p*< 0.05, ^**^*p*< 0.01. **(H)** Quantitation of colony formation assays was displayed in the indicated cells. ^*^*p*< 0.05, ^**^*p*< 0.01. **(I)** Quantitation of the apoptosis rate in the indicated cells upon treatment with gemcitabine by Flow-cytometry. ^**^*p*< 0.01. **(J)** Inhibitory effect on tumor growth of treatments with gemcitabine in the indicated CDX models. n=6, ^**^*p*< 0.01. **(K)** The tumor wight in the indicated CDX models upon treated with gemcitabine. n=6, ^**^*p*< 0.01.


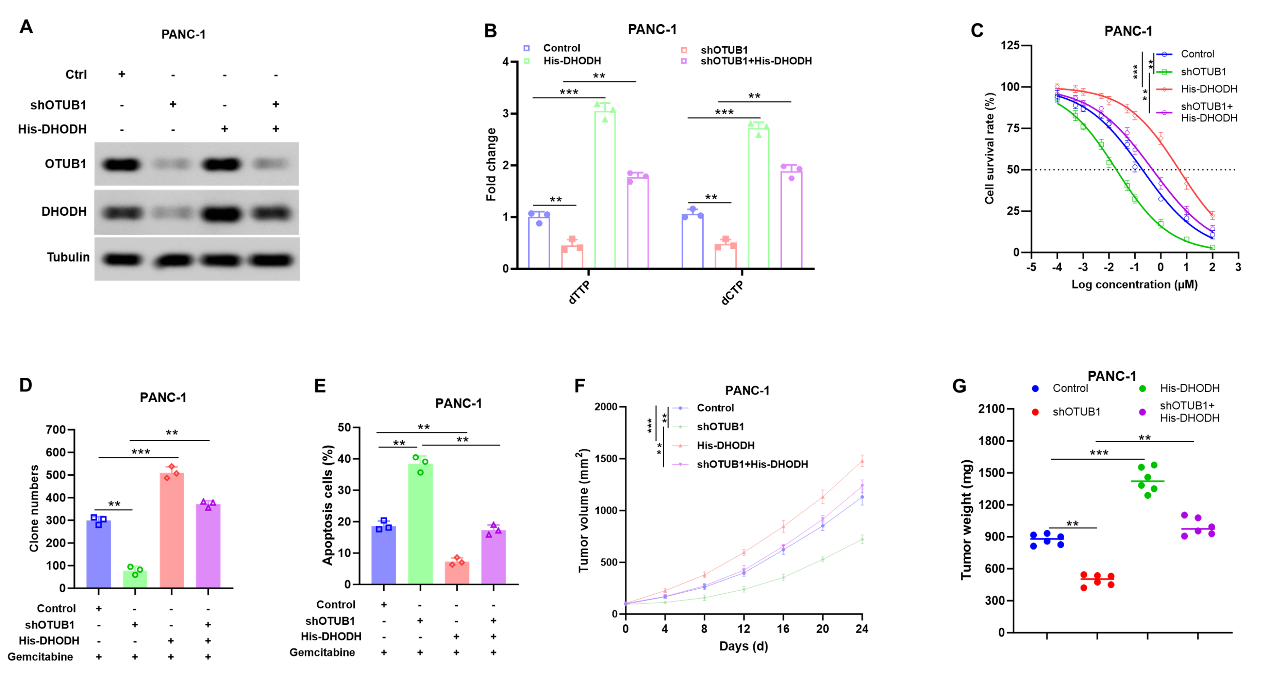


**Figure S7. Oncogenic effect of OTUB1 is dependent on DHODH enhancement. (A)** Western blot analysis showing OTUB1 and DHODH expression in the indicated cells upon treatment with gemcitabine. Tubulin was used as the internal standard. **(B)** Fold changes in dCTP and dTTP levels from the indicated cells were monitored using a fluorescence-based assay. ^**^*p*< 0.01, ^***^*p*< 0.001. **(C)** Sensitivity to gemcitabine in the indicated cells were detected by CCK-8 assay. ^**^*p*< 0.01, ^***^*p*< 0.001. **(D)** Quantitation of colony formation assays was displayed in the indicated cells. ^**^*p*< 0.01, ^***^*p*< 0.001. **(E)** Quantitation of the apoptosis rate in the indicated cells upon treatment with gemcitabine by Flow-cytometry. ^**^*p*< 0.01. **(F)** Inhibitory effect on tumor growth of treatments with gemcitabine in the indicated CDX models. n=6, ^**^*p*< 0.01, ^***^*p*< 0.001. **(G)** The tumor wight in the indicated CDX models upon treated with gemcitabine. n=6, ^**^*p*< 0.01, ^***^*p*< 0.001.


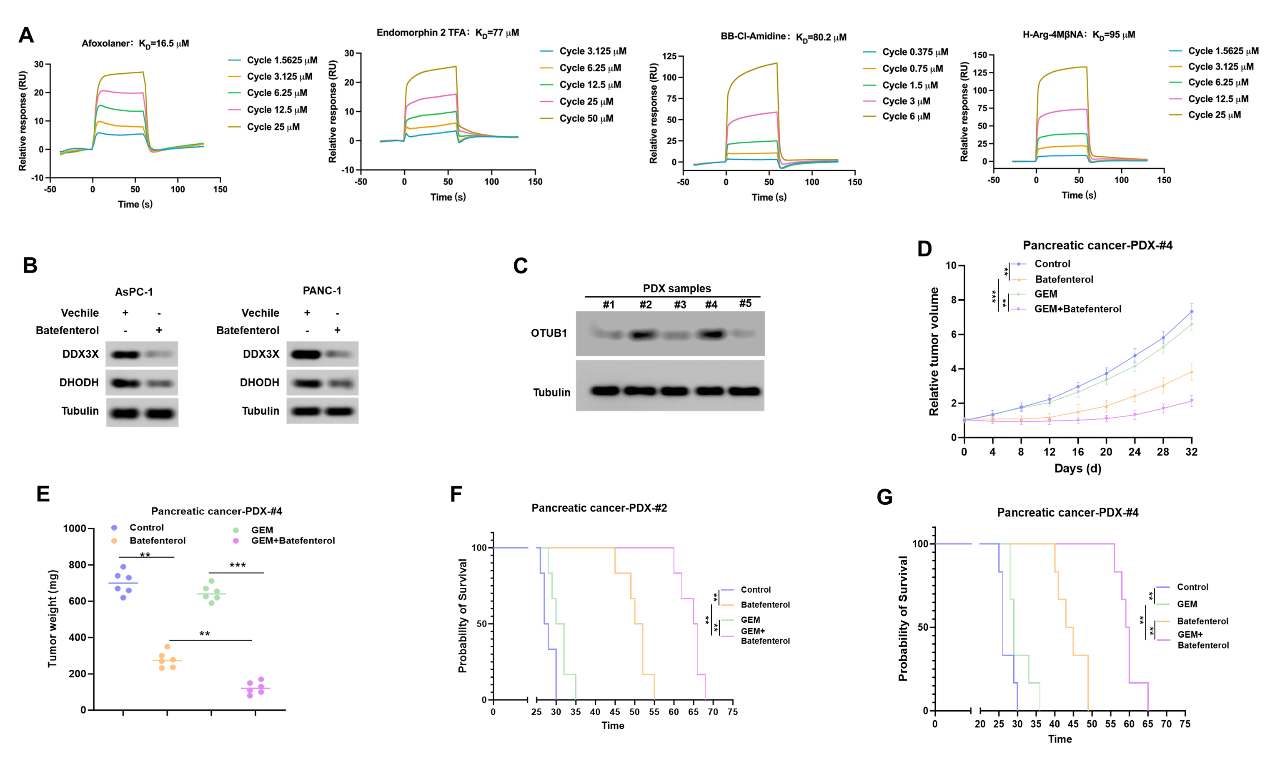


**Figure S8. Identiﬁcation and characterization of Batefenterol as a OTUB1 inhibitor and gemcitabine sensitizer. (A)** Kinetic constant (KD) analysis of Afoxolaner, Endomorphin 2, BB-Cl-Amidine, and H-Arg-4MβNA interacting with OTUB1 using SPR assay, respectively. **(B)** Western blot analysis showing DDX3X and DHODH expression in AsPC-1 and PANC-1 cells upon treatment with or without Batefenterol. Tubulin was used as the internal standard. **(C)** Western blot analysis showing OTUB1 expression in human pancreatic tumor tissues for PDX models. Tubulin was used as the internal standard. **(D and E)** Tumor growth (D) and tumor weight (E) in OTUB1^high^ PDXs treated with gemcitabine, batefenterol, or both. n=6, ^**^*p*< 0.01, ^***^*p*< 0.001. (F and G) Kaplan-Meier analysis of OTUB1^high^ PDXs treated with gemcitabine, batefenterol, or both. n=6, ^**^*p*< 0.01, ^***^*p*< 0.001.
