# Supplementary material for: The deubiquitylase OTUB1 drives gemcitabine resistance in pancreatic cancer by enhancing pyrimidine metabolism through modulating DHODH mRNA stability
Source: Cell Death Dis. 2025 Oct 6;16(1):697. doi: 10.1038/s41419-025-08001-4 (PMC12501277; doi:10.1038/s41419-025-08001-4)
Supplement: Supplementary file 2 — Table S1 [file 41419_2025_8001_MOESM2_ESM.docx]

**Supplementary Table 1: Sequences of primers.**

| **Gene** | **Primer** |
| --- | --- |
| OTUB1  (human) qPCR | F: 5’- AGCAGGACCGAATTCAGCAA-3’  R: 5’- GGAGAATCCGAAAGCCCGAT-3’ |
| DDX3X  (human), qPCR | F: 5’-TTCGCGGTGGAACAAACACT-3’  R: 5’-GAGGAATATAGCGCCCTTTGC-3’ |
| DHODH (human), qPCR | F: 5’-GCTGCAGGATTTGACAAGCA-3’  R: 5’-ACTGAAAGCCCGTGACTGTT-3’ |
| GAPDH  (human), qPCR | F: 5’-AATGAATGGGCAGCCGTTAG- 3’  R: 5’-AGAGTTAAAAGCAGCCCTGG-3’ |
