# Supplementary material for: The deubiquitylase OTUB1 drives gemcitabine resistance in pancreatic cancer by enhancing pyrimidine metabolism through modulating DHODH mRNA stability
Source: Cell Death Dis. 2025 Oct 6;16(1):697. doi: 10.1038/s41419-025-08001-4 (PMC12501277; doi:10.1038/s41419-025-08001-4)
Supplement: Supplementary file 3 — Table S2 [file 41419_2025_8001_MOESM3_ESM.doc]

**Supplementary Table 2**. shRNA sequences used in our research.

| **shRNA** | **Sense (5’-3’)** | **Anti-sense (5’-3’)** |
| --- | --- | --- |
| shRNA-NC | TTCTCCGAACGGTCACGT | ACGTGACCGTTCGGAGAA |
| shRNA-OTUB1#1 | actgtcaaggagttctgccag | CTGGCAGAACTCCTTGACAGT |
| shRNA-OTUB1#2 | TGTGGTTGTAAATGGTCCTAT | ATAGGACCATTTACAACCACA |
| shRNA-DDX3X | agacagttcagggtggagttc | GAACTCCACCCTGAACTGTCT |
| shRNA-DHODH | atggcttgcggagagtgcaca | TGTGCACTCTCCGCAAGCCAT |
