# Supplementary material for: The deubiquitylase OTUB1 drives gemcitabine resistance in pancreatic cancer by enhancing pyrimidine metabolism through modulating DHODH mRNA stability
Source: Cell Death Dis. 2025 Oct 6;16(1):697. doi: 10.1038/s41419-025-08001-4 (PMC12501277; doi:10.1038/s41419-025-08001-4)

Uncropped version of all Western blots in main figures and supplementary figures

Figure 1E top

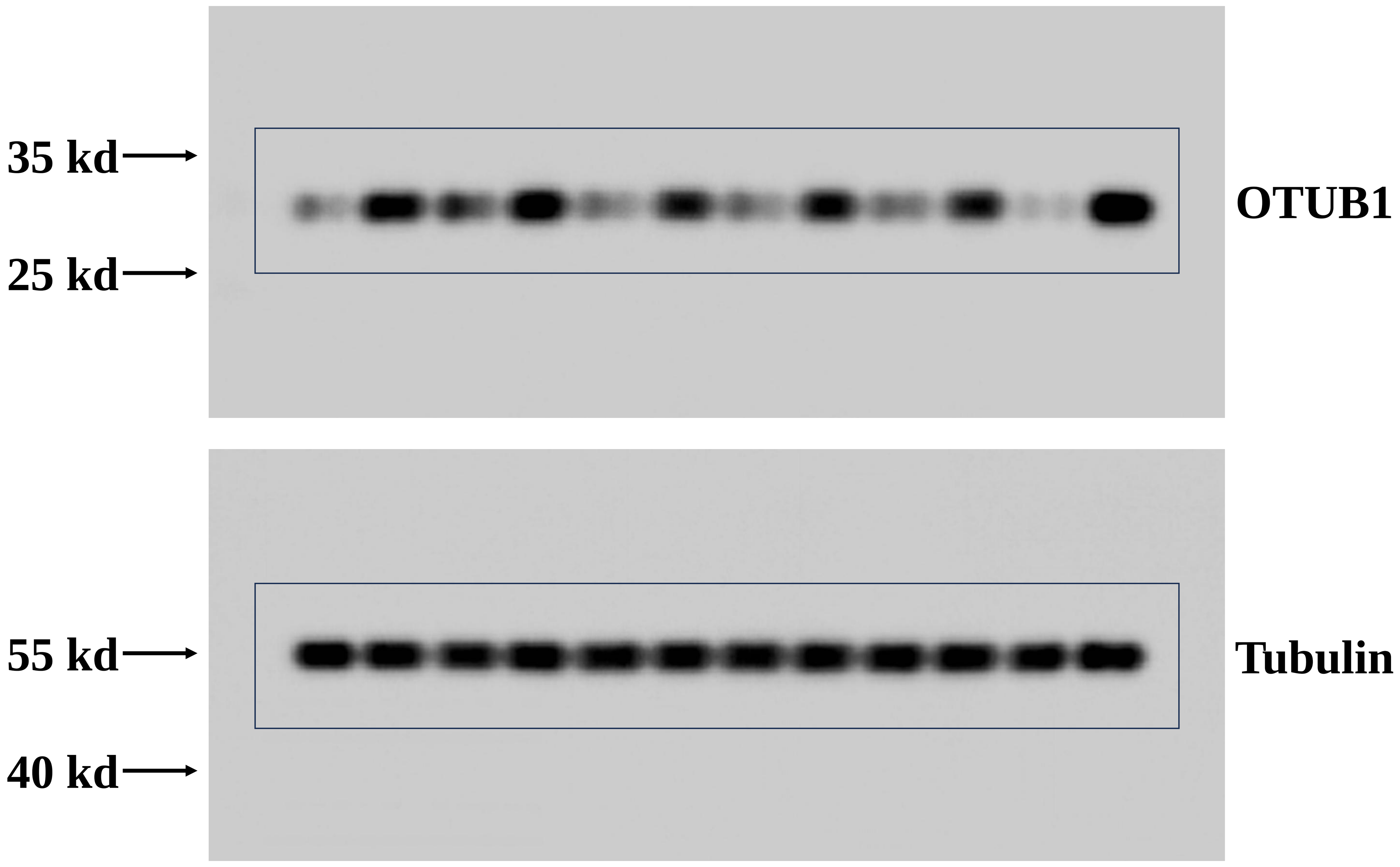

Figure 1E bottom

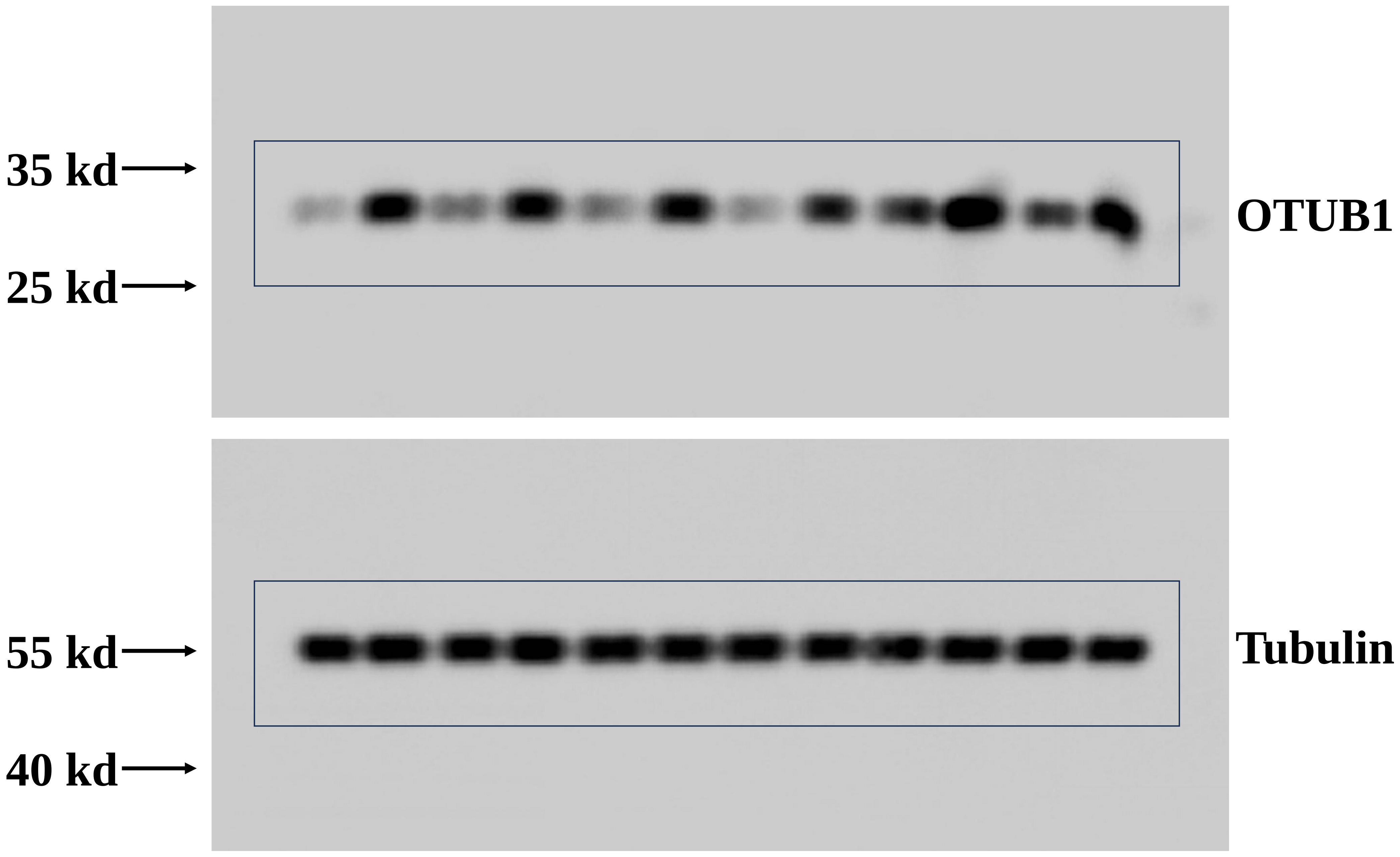

Figure 2C

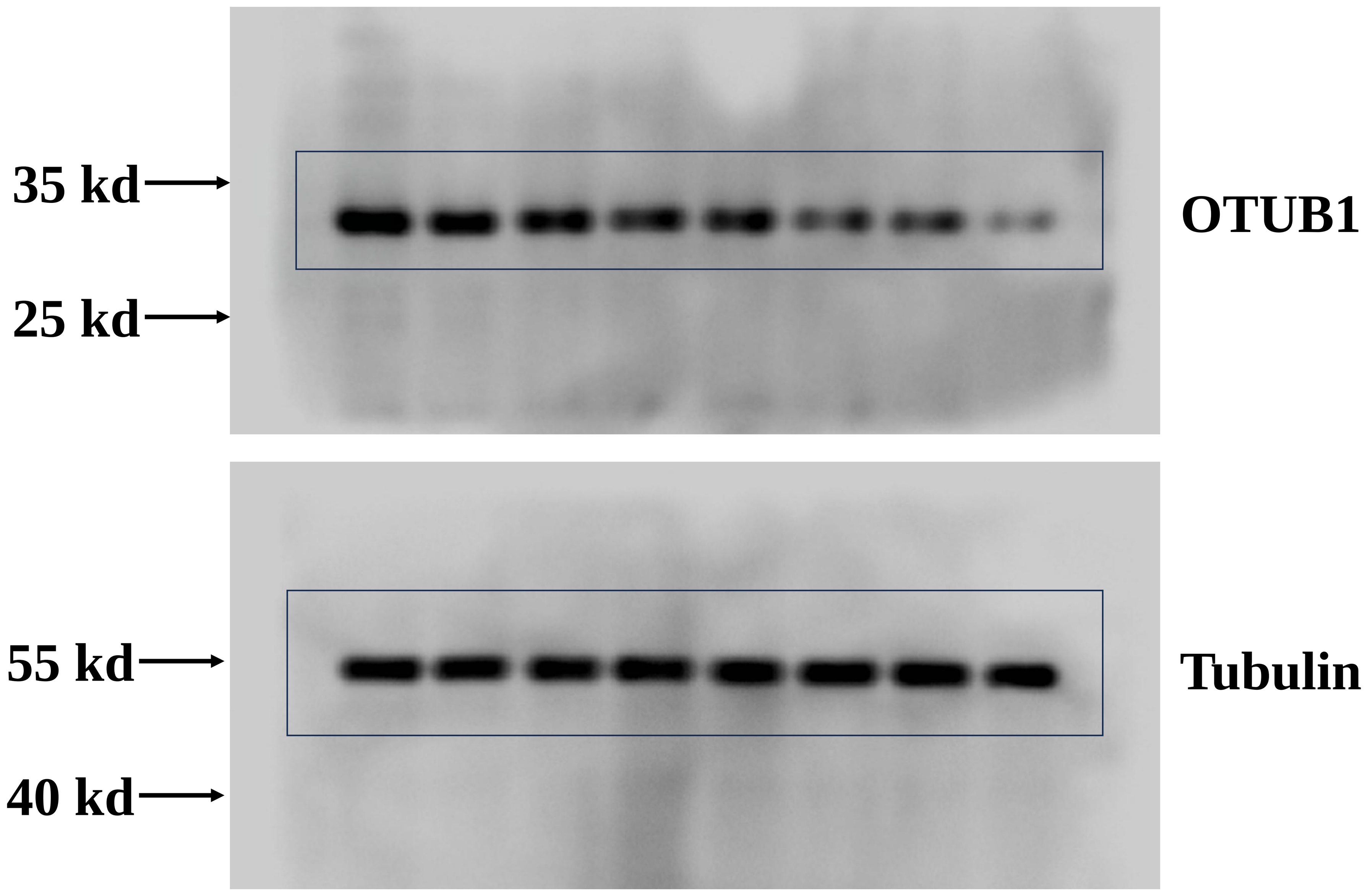

Figure 2E

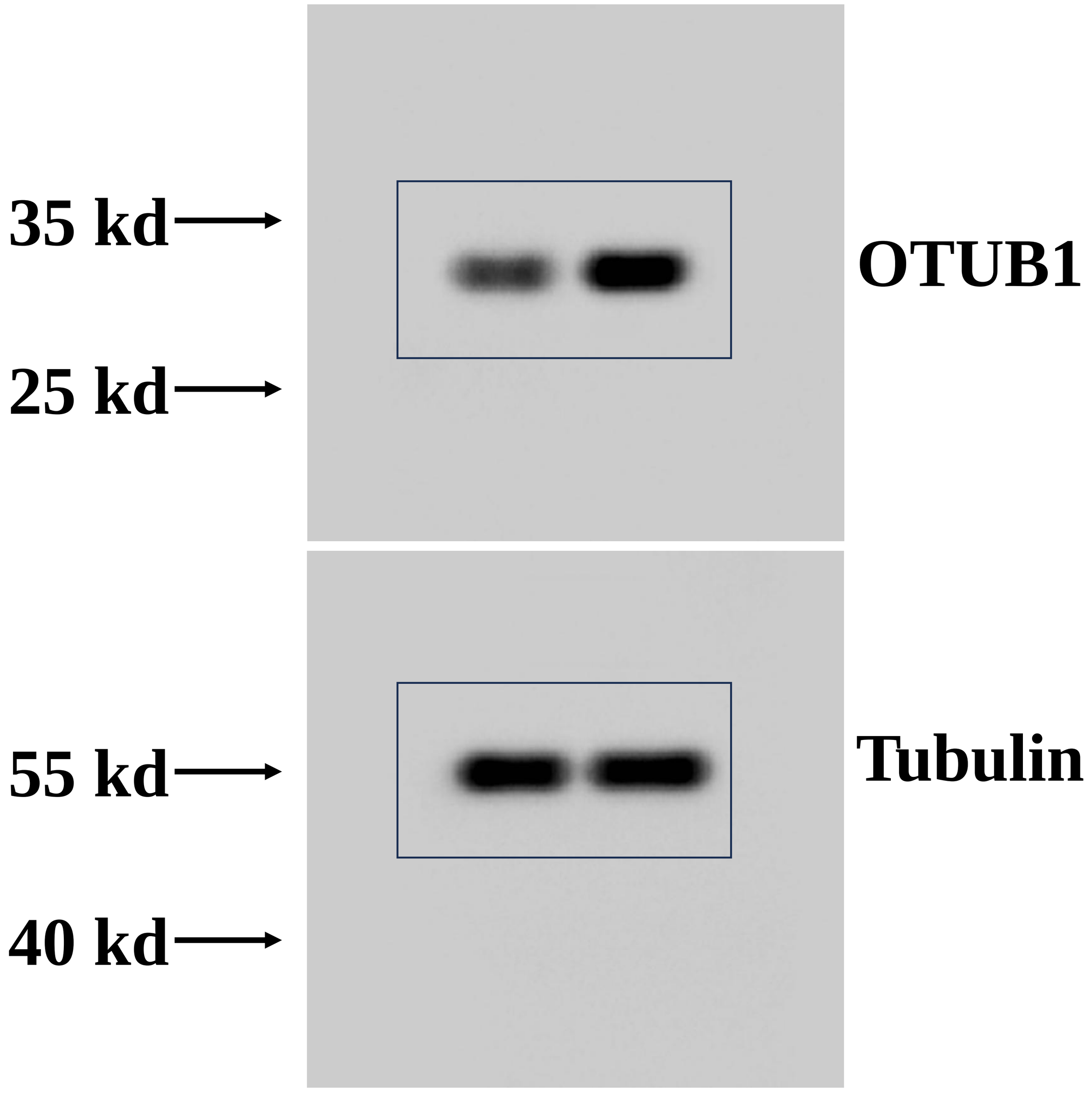

Figure 2L left

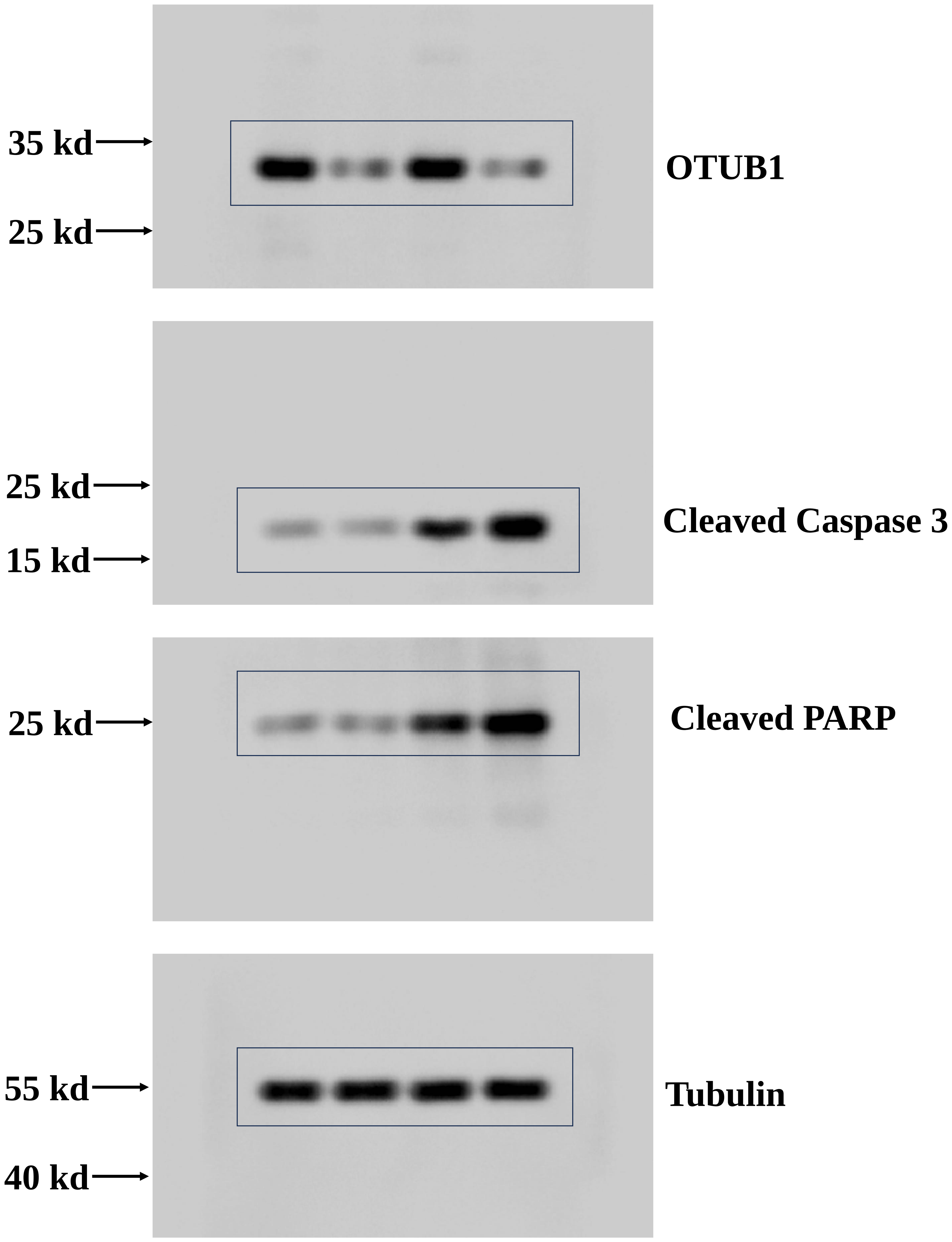

Figure 2L right

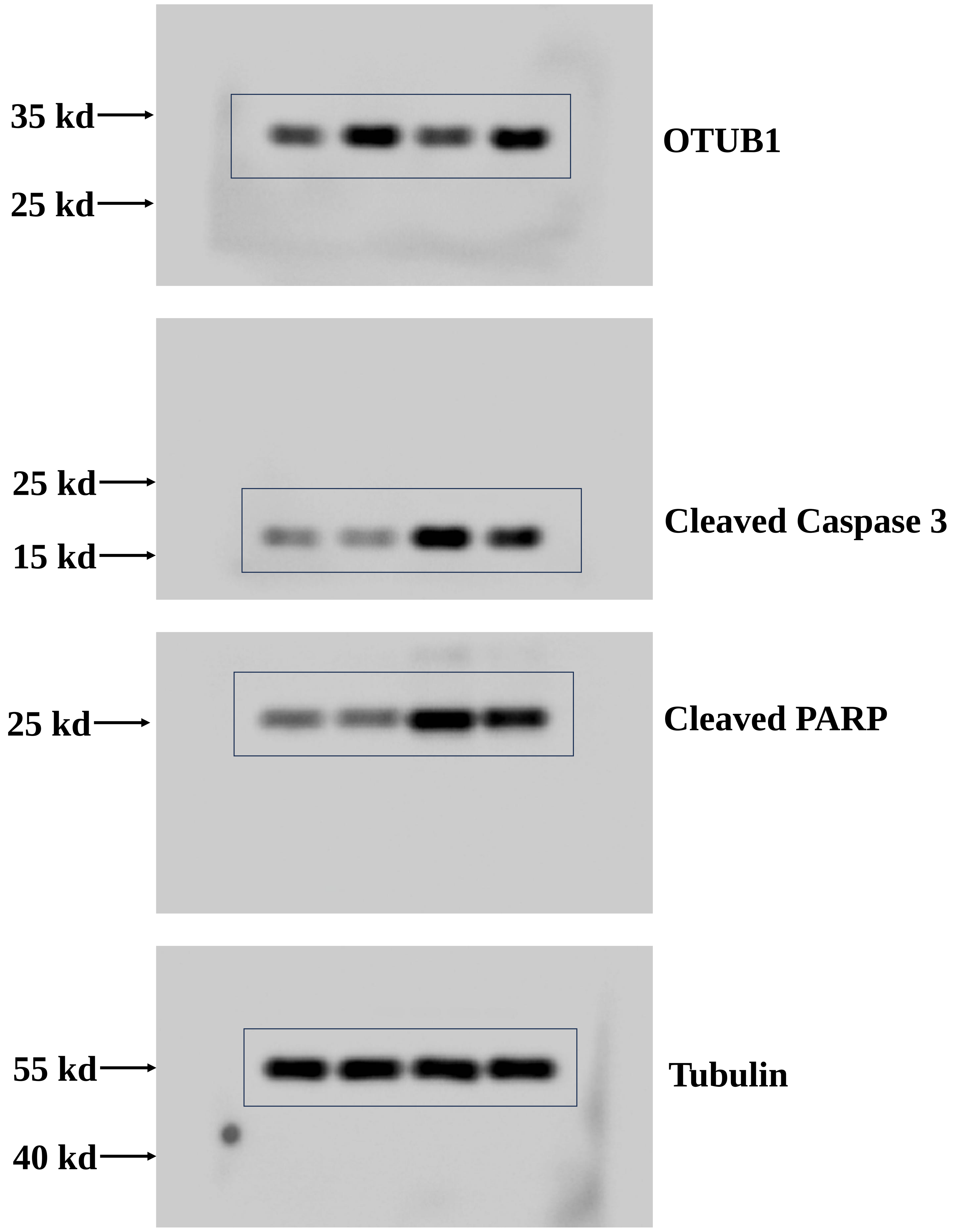

Figure 4I

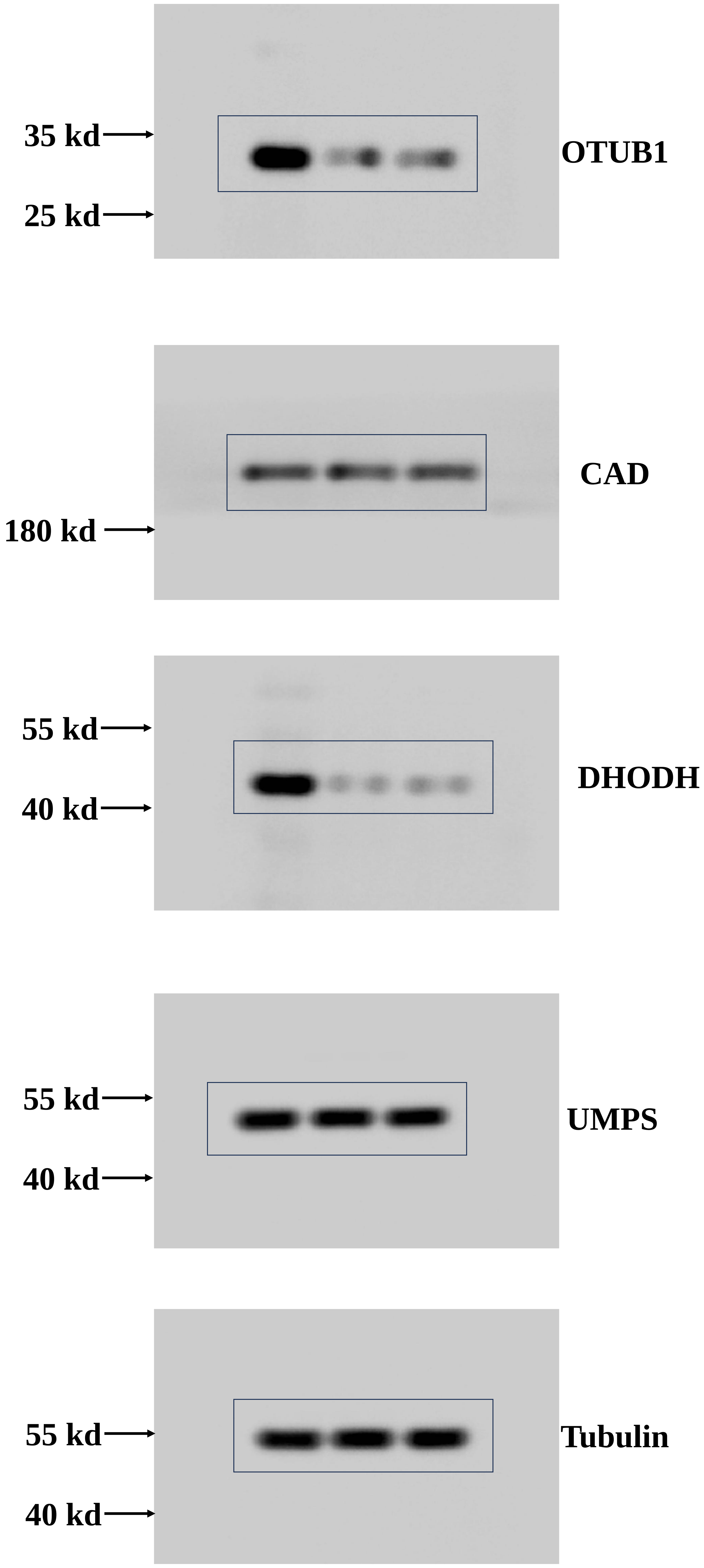

Figure 4K

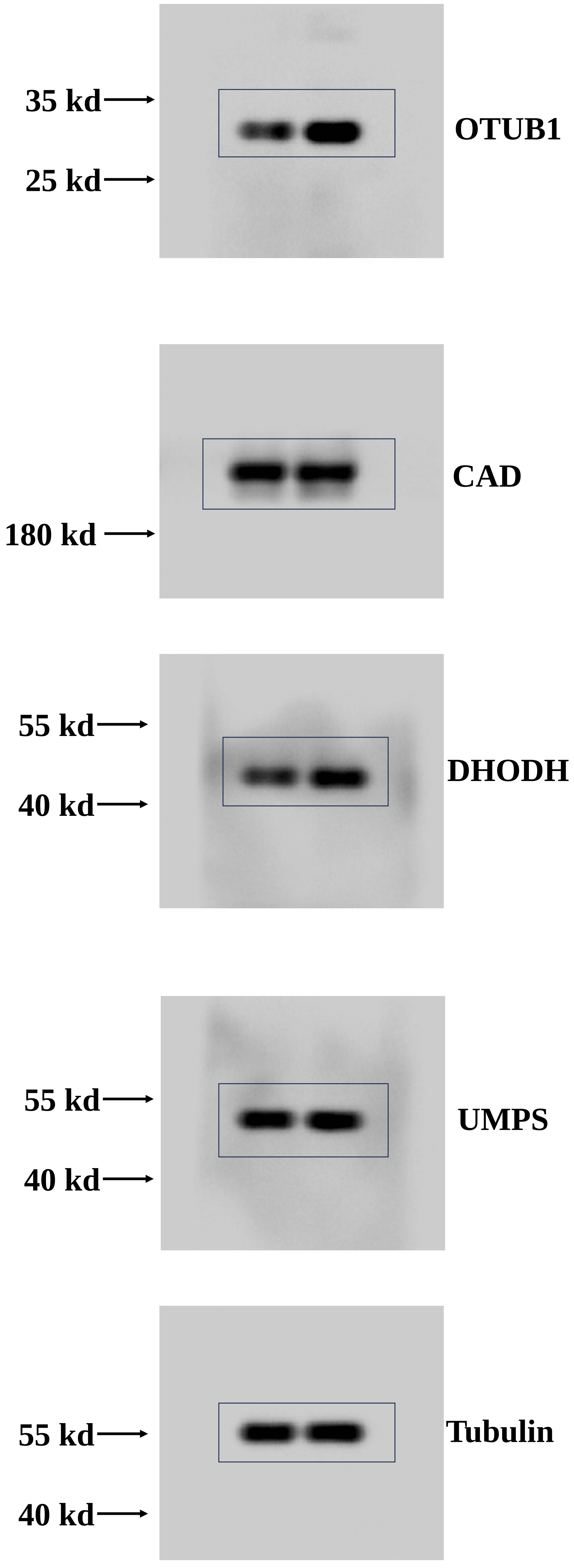

Figure 5A

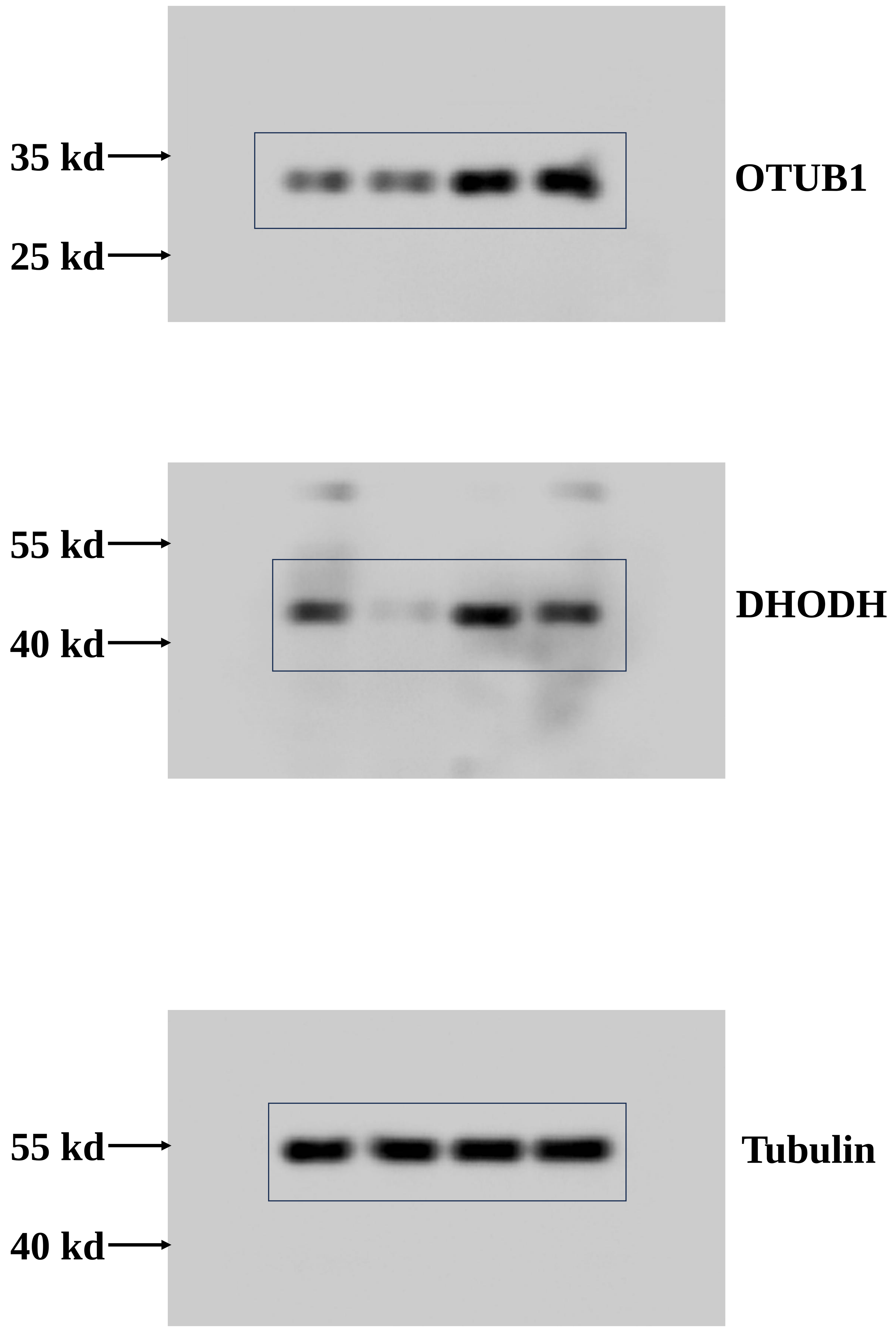

Figure 5K

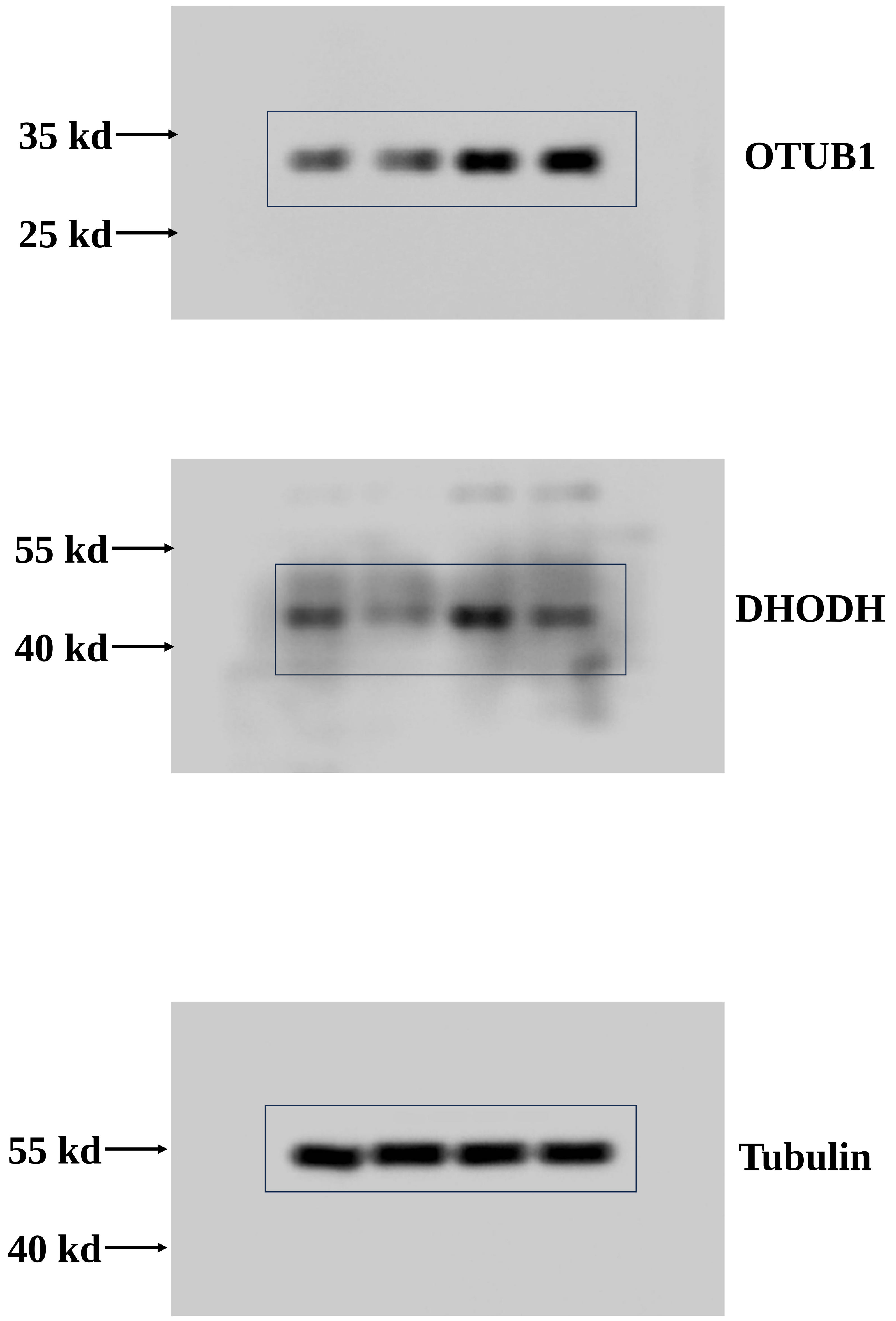

Figure 6A top

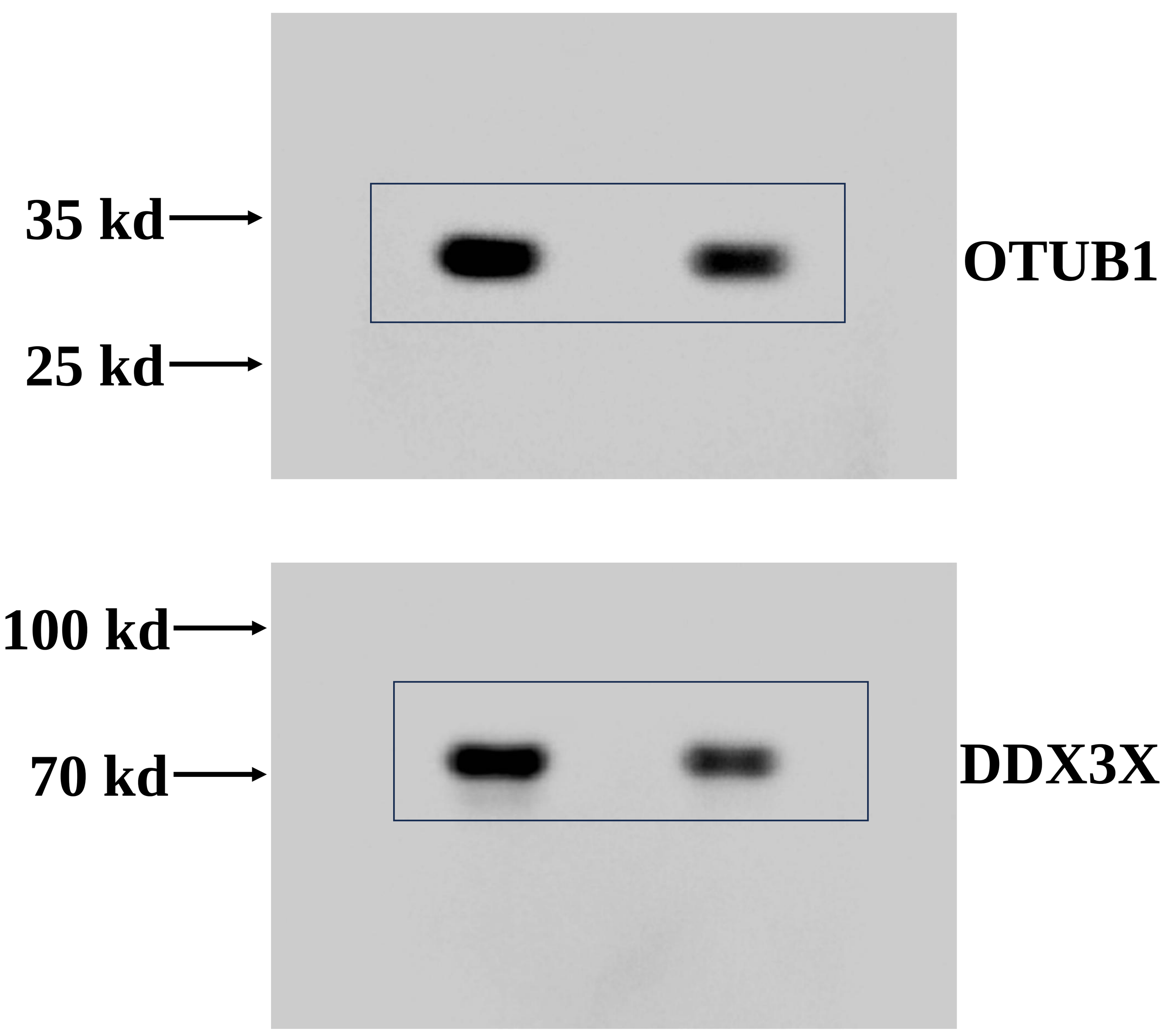

Figure 6A bottom

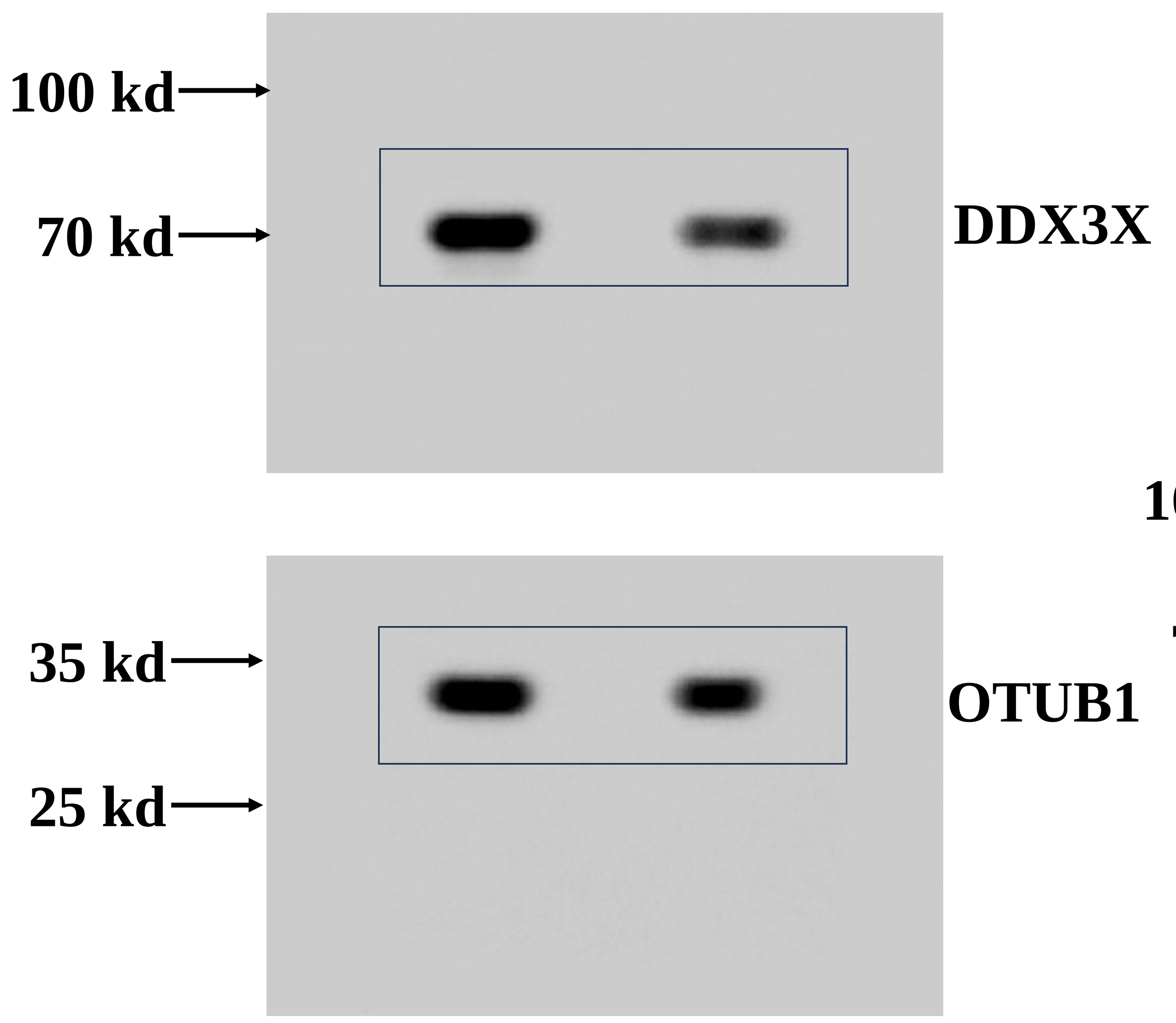

Figure 6B

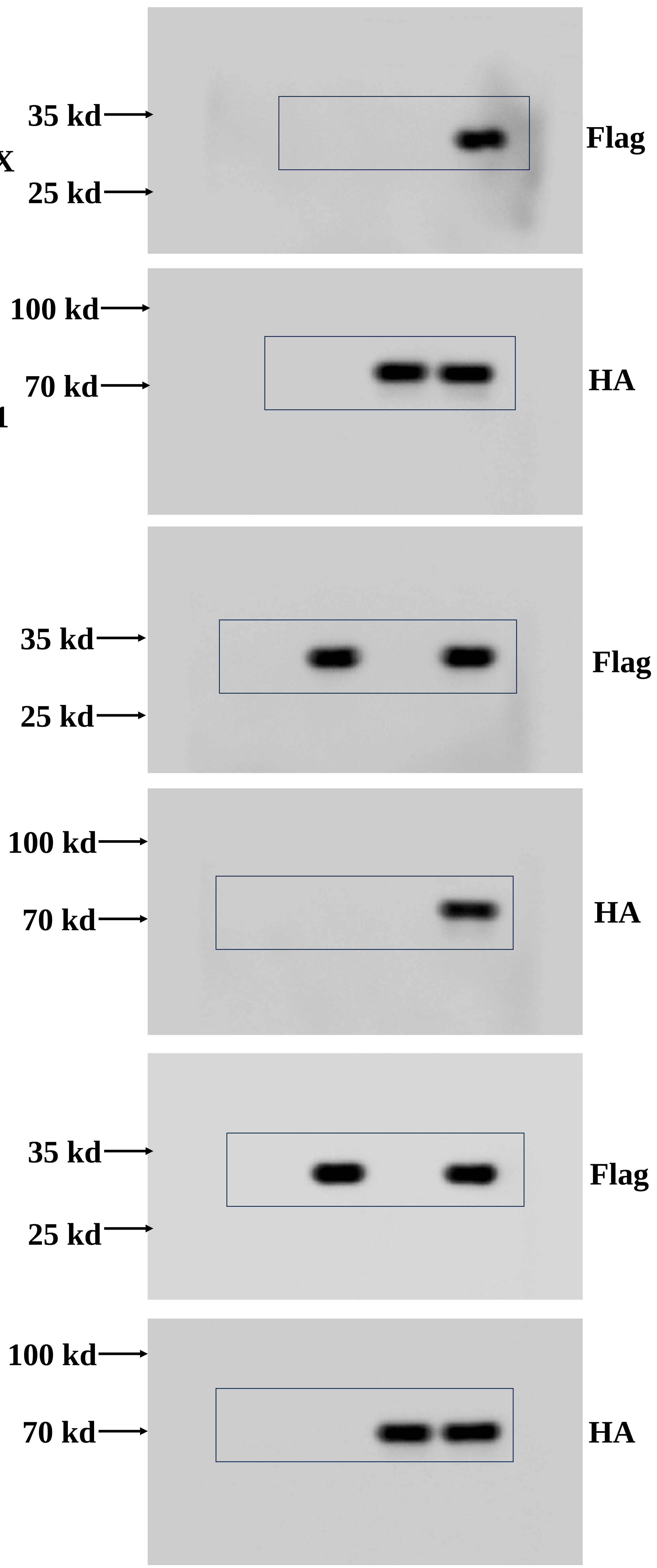

Figure 6G (left)

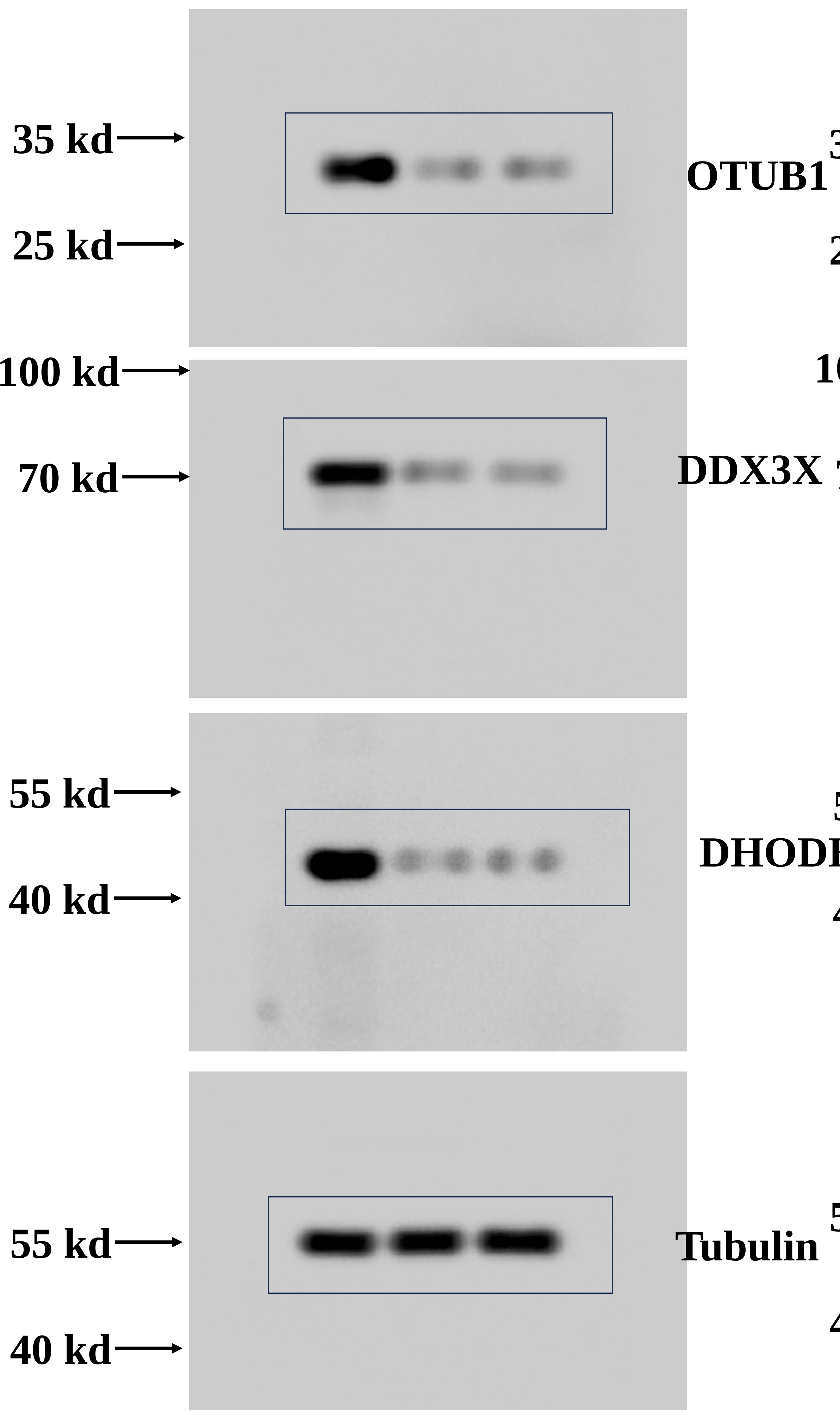

Figure 6G (right)

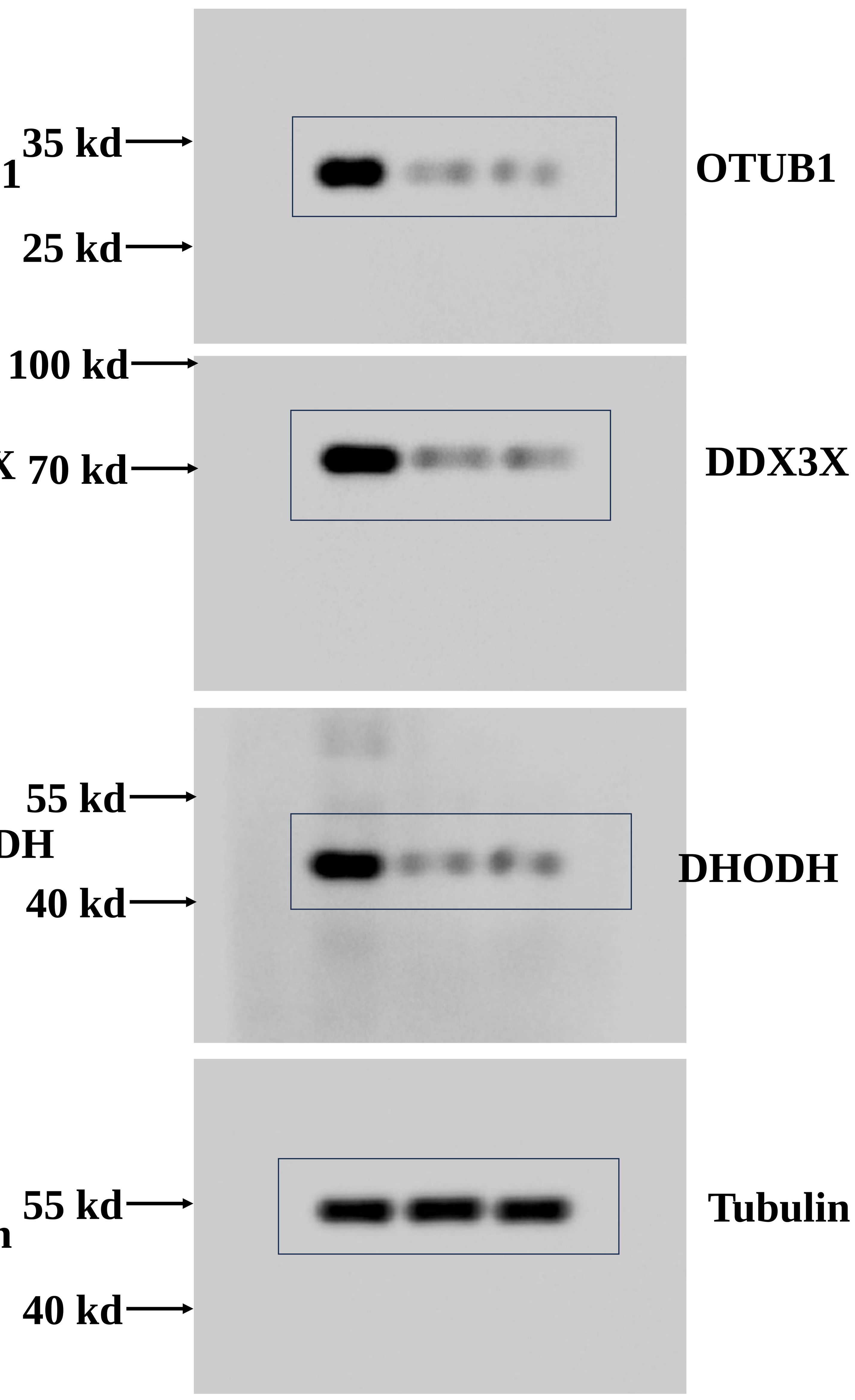

Figure 6H

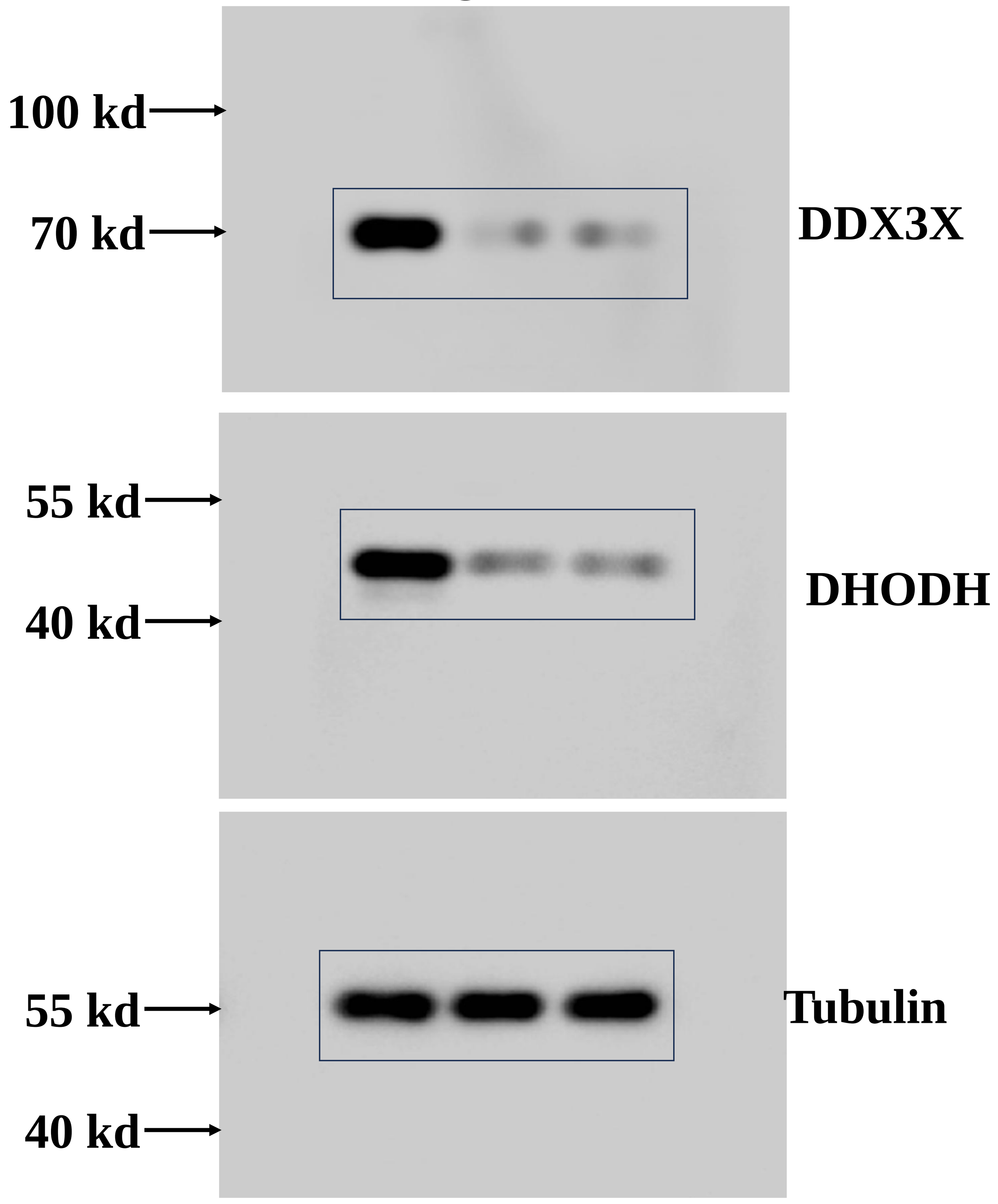

Figure 6F

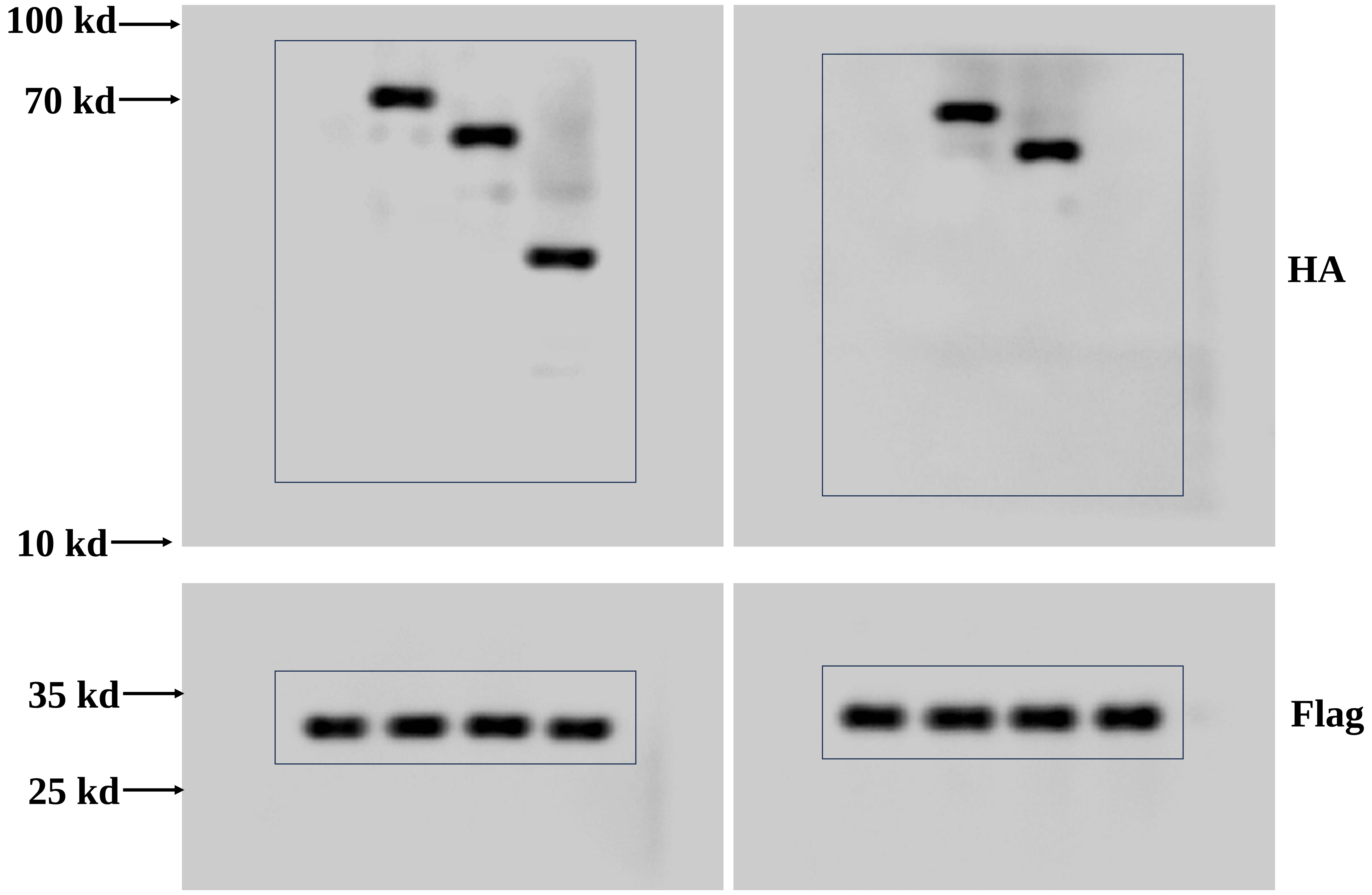

Figure 6M

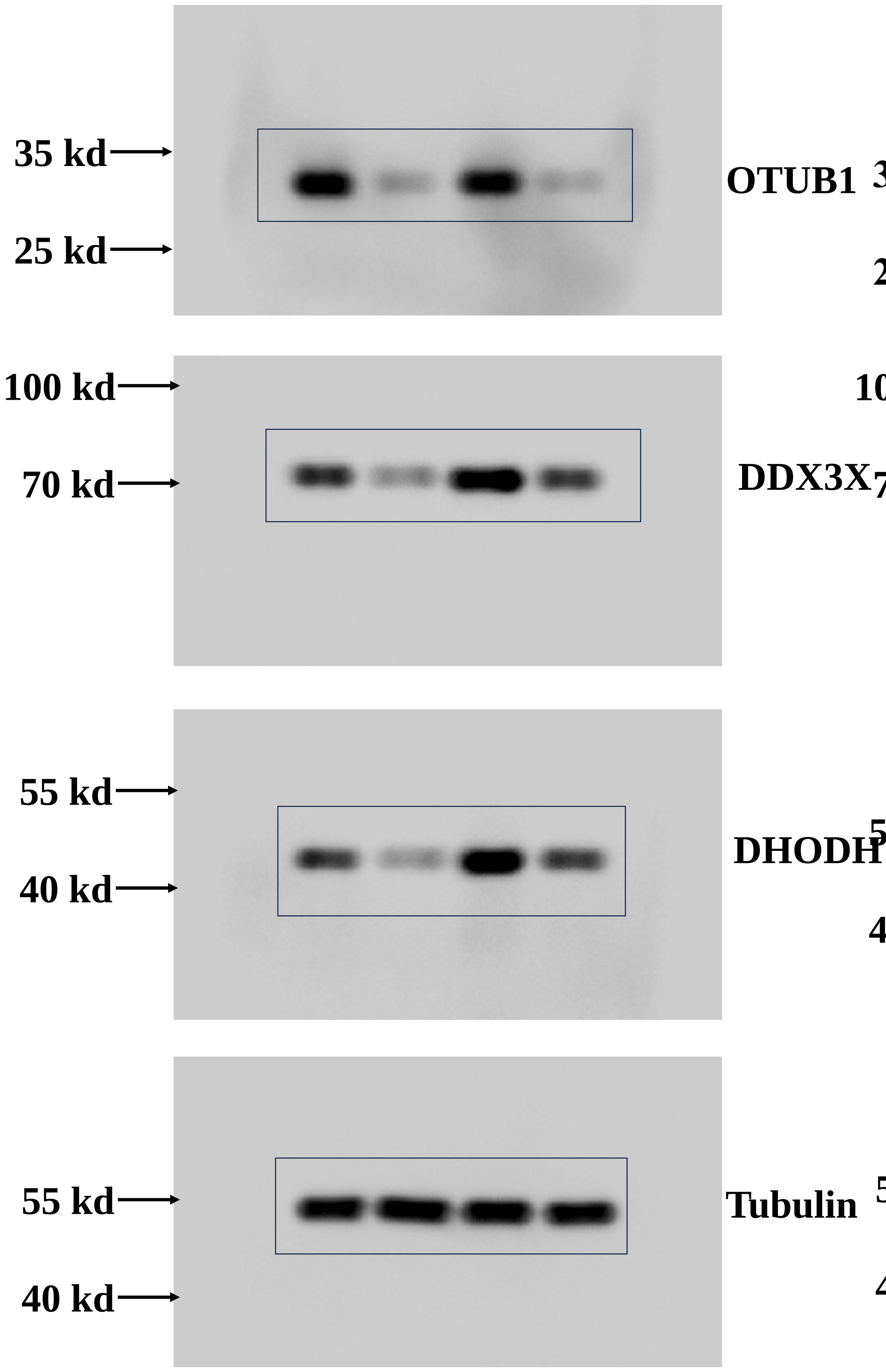

Figure 6N

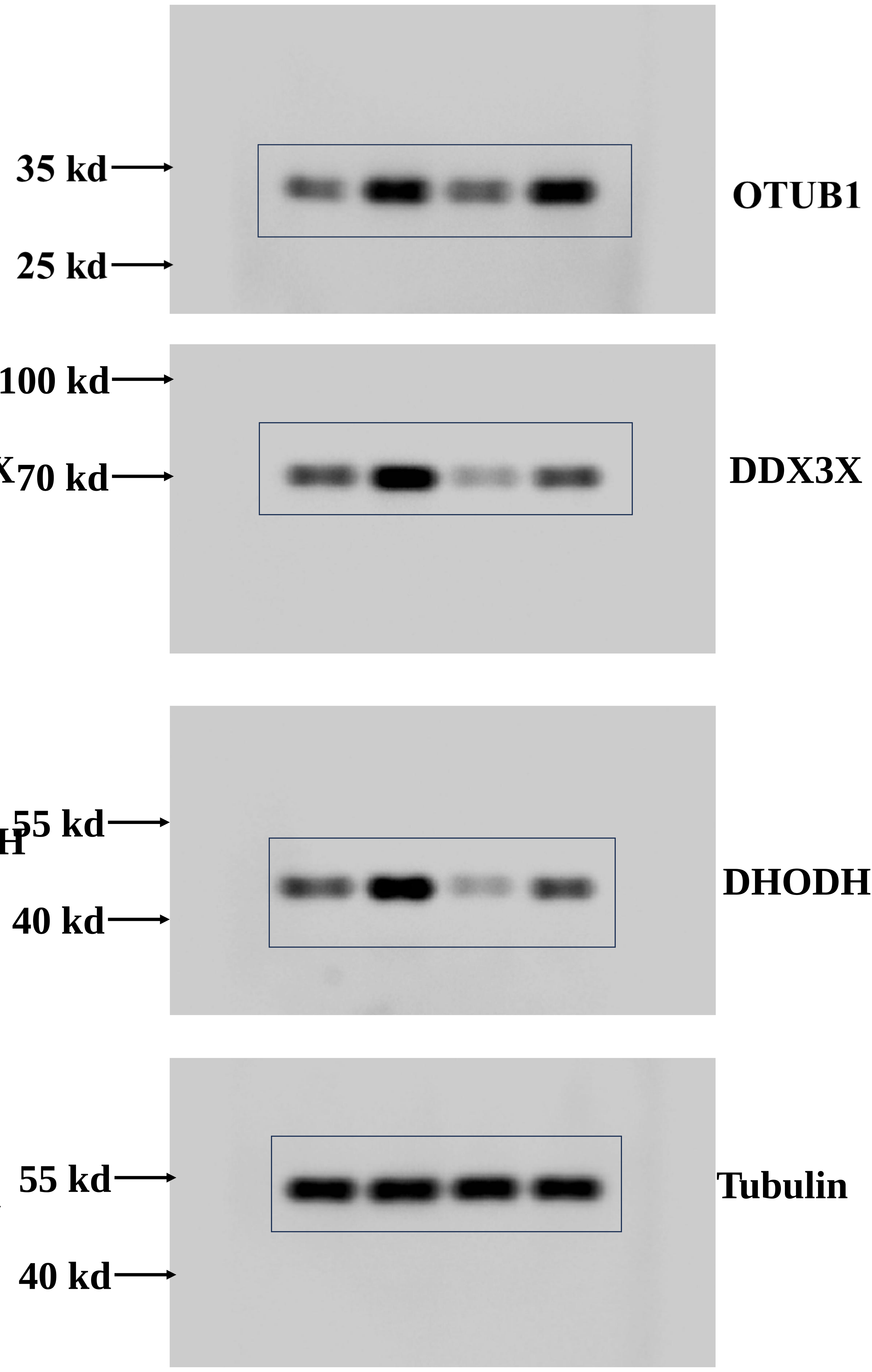

Figure 6K

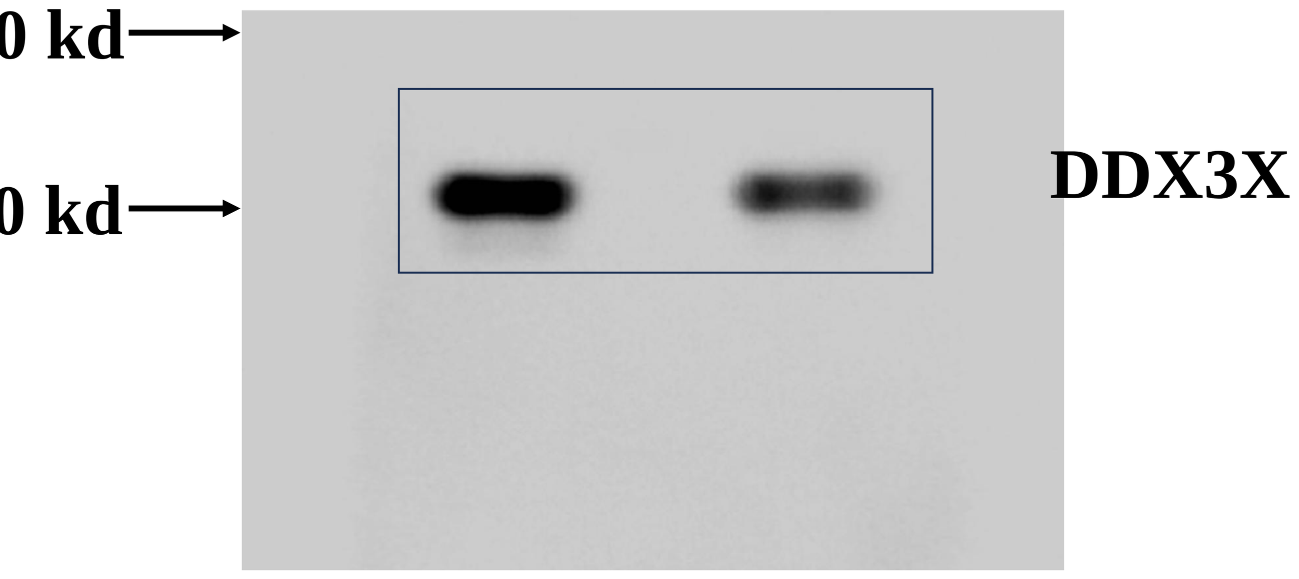

Figure 7A

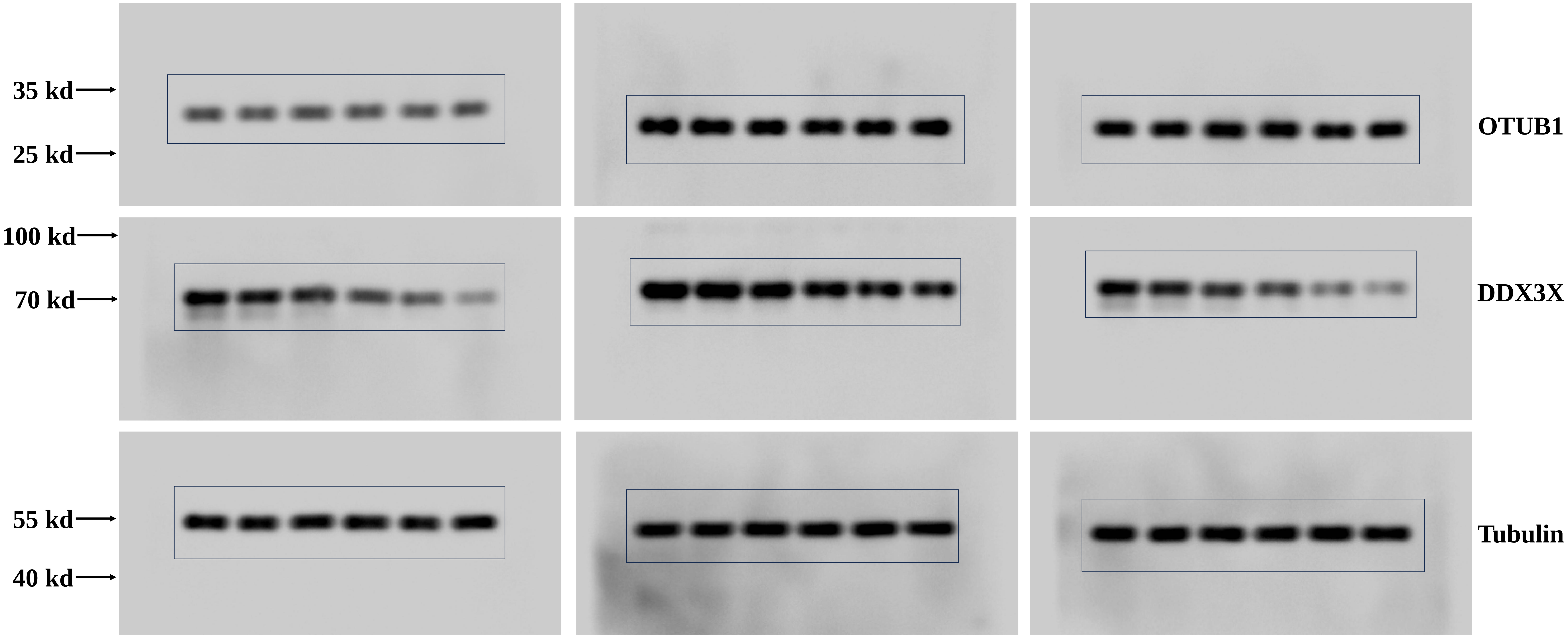

Figure 7C

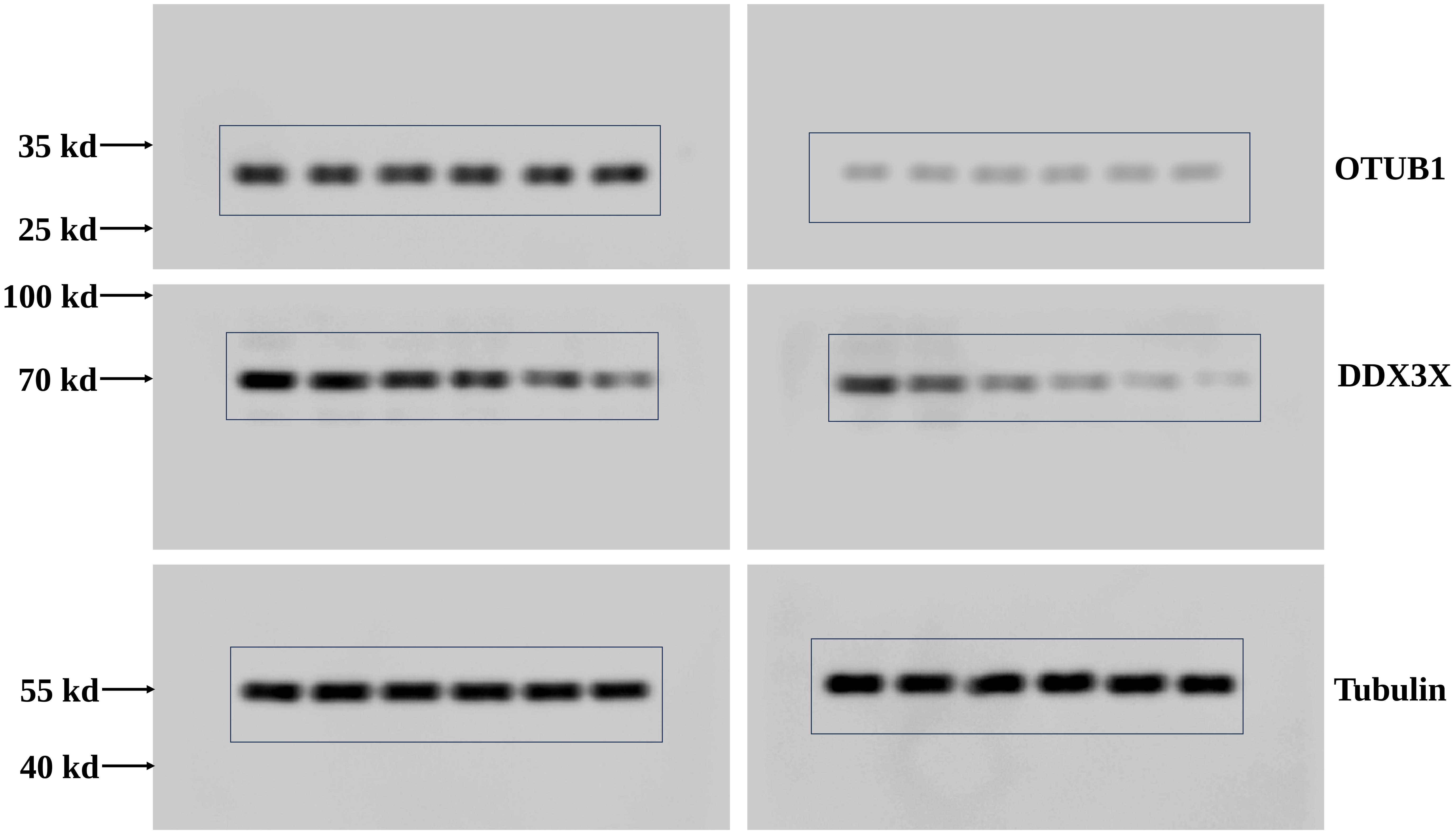

Figure 7D

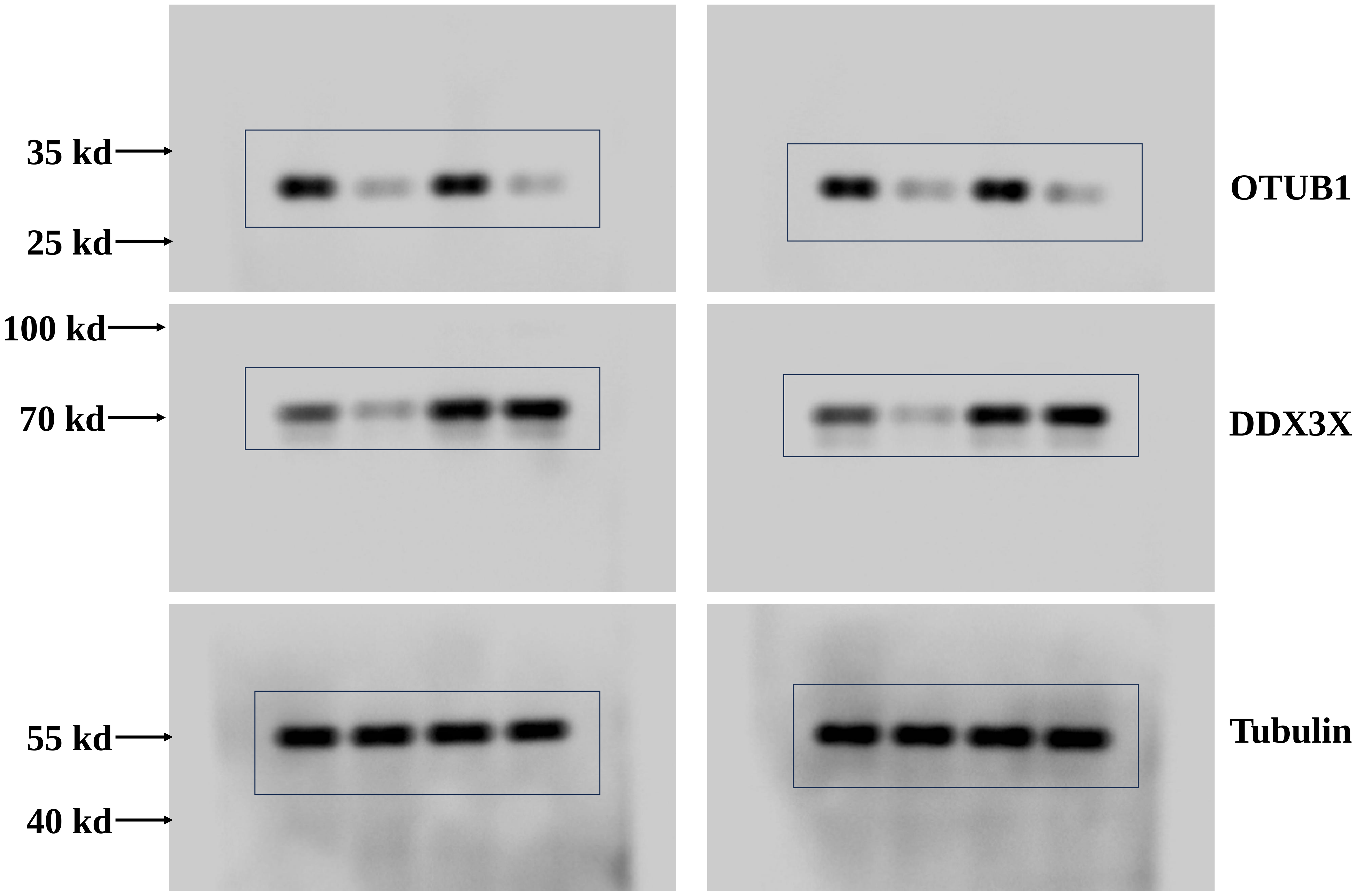

Figure 7E

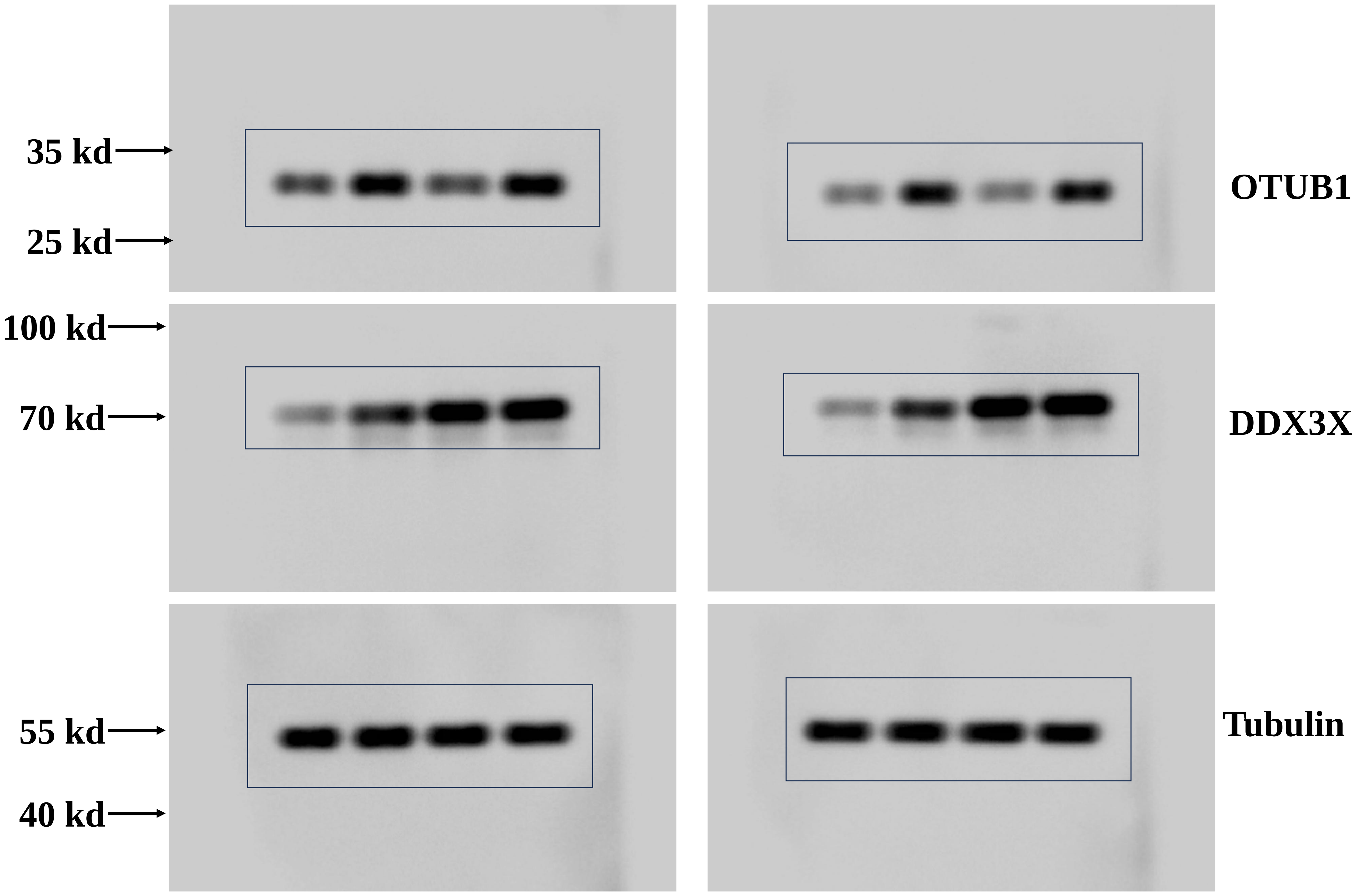

Figure 7F

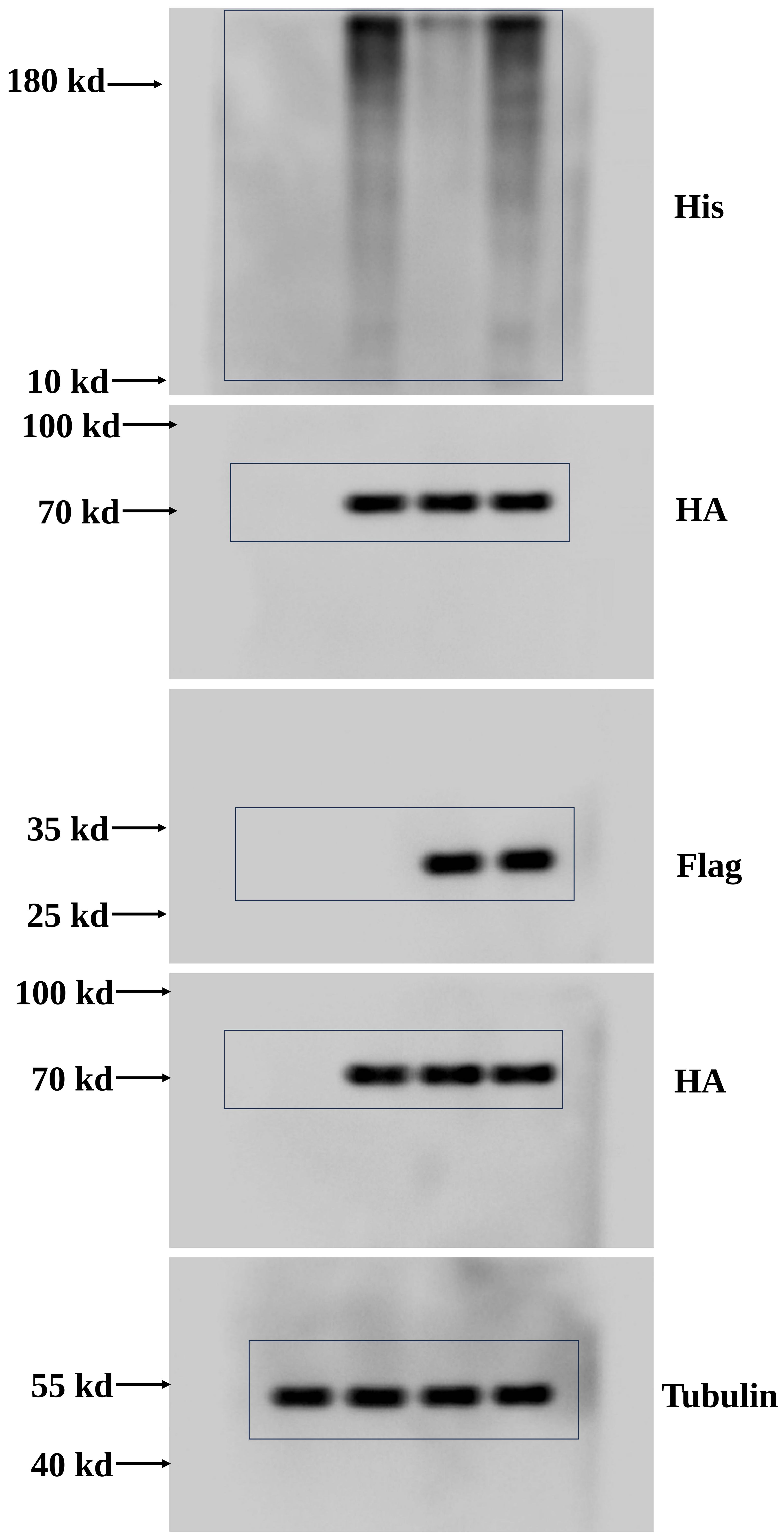

Figure 7G

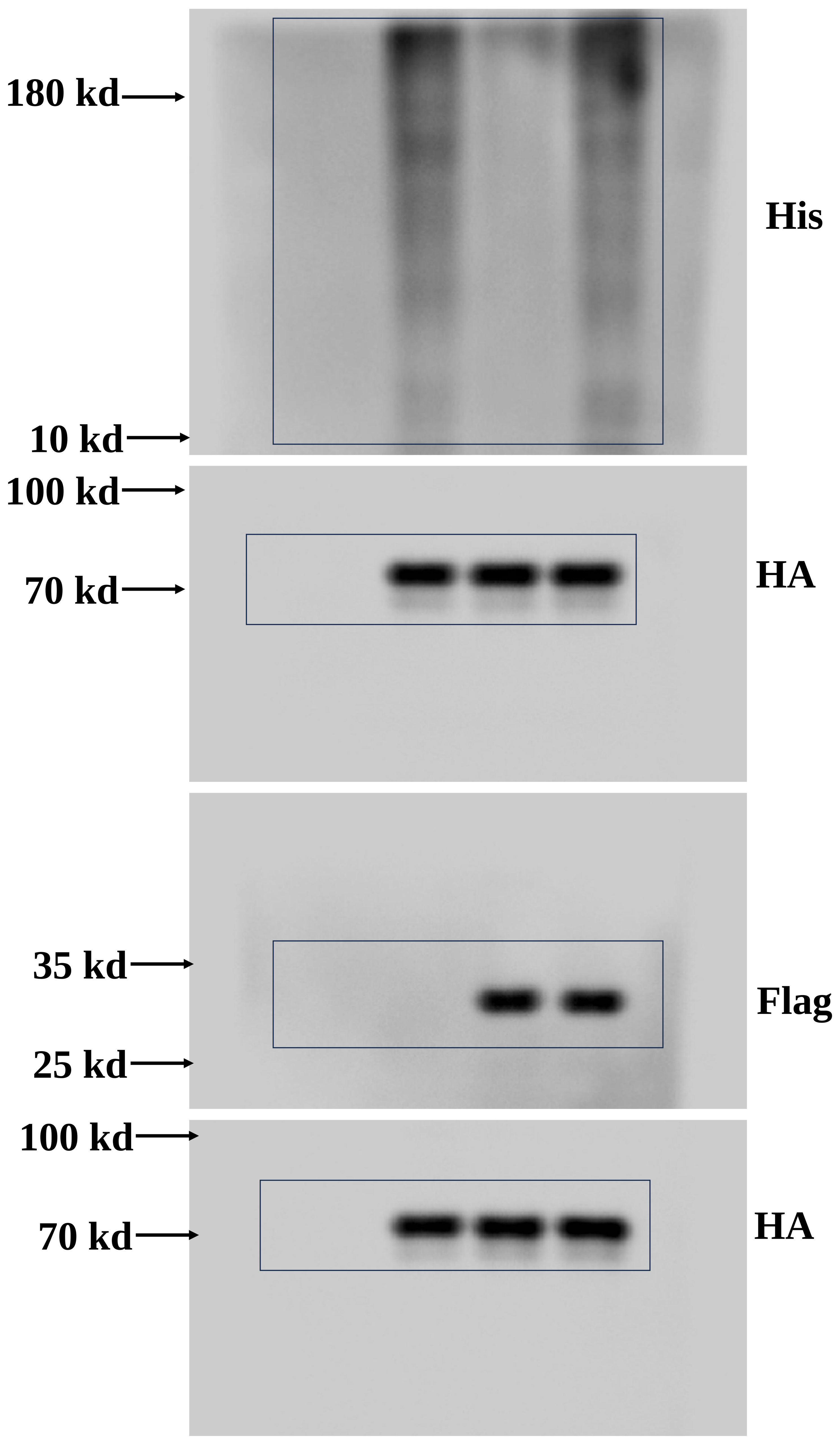

Figure 7H

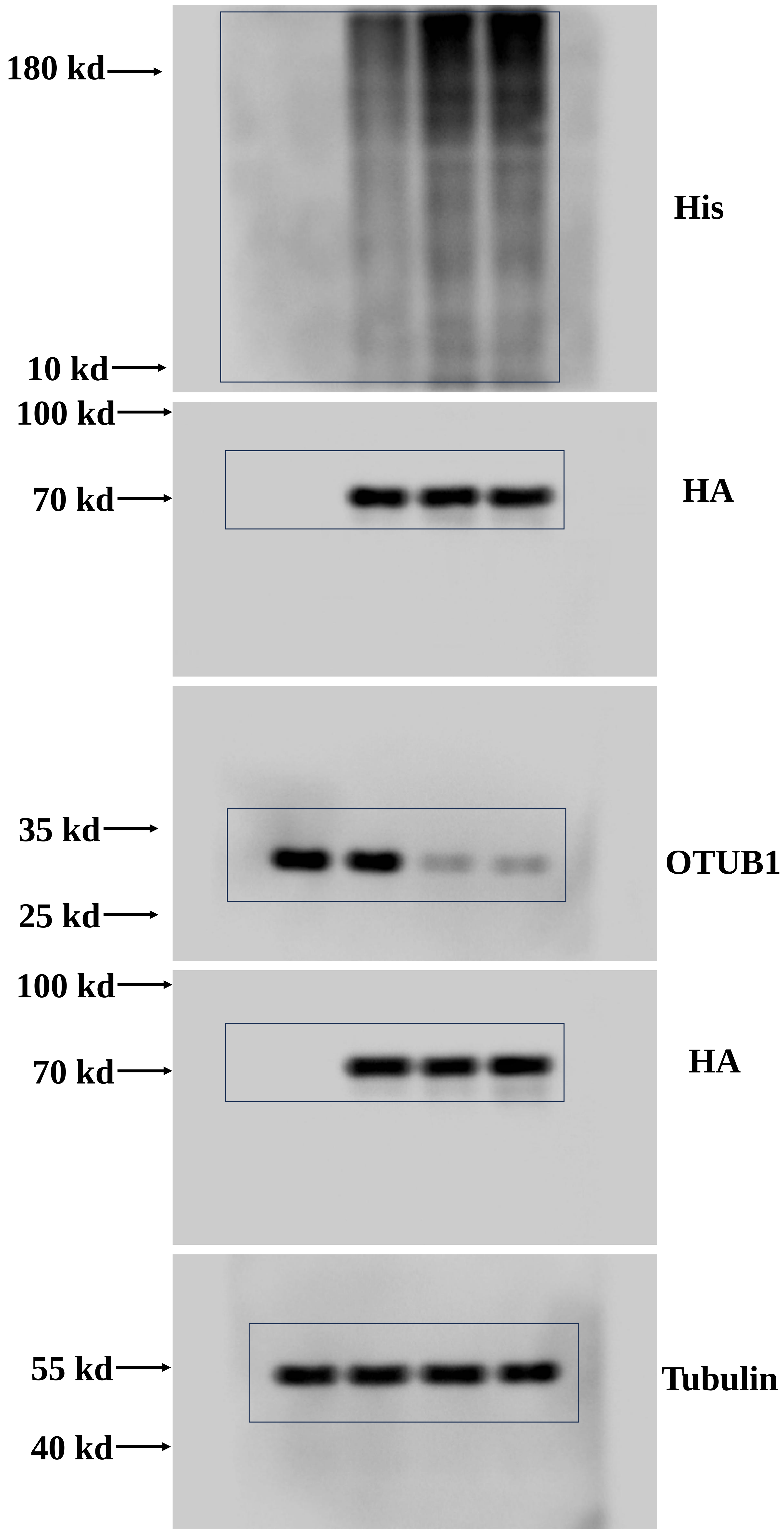

Figure 7I

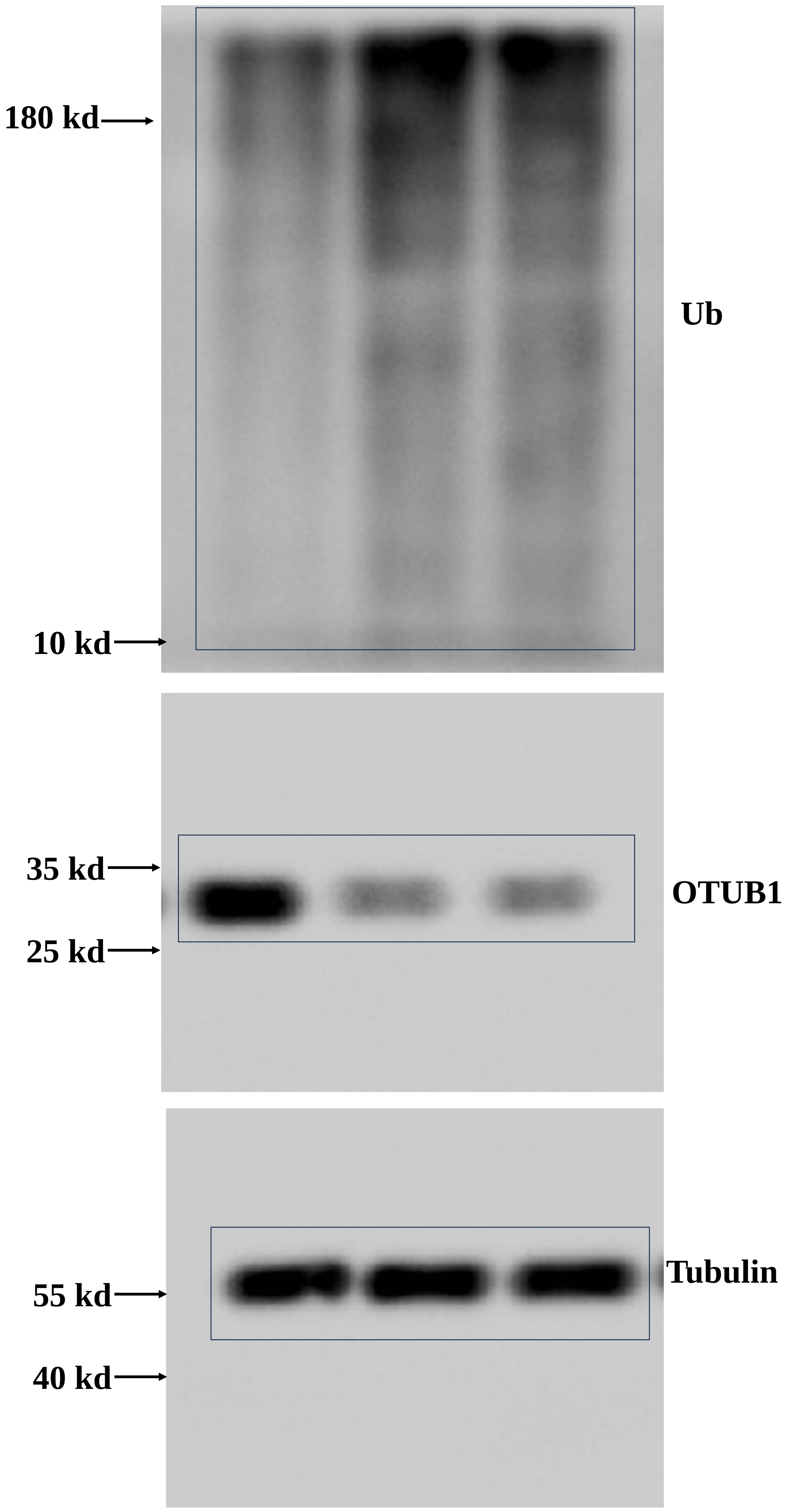

Figure 7J

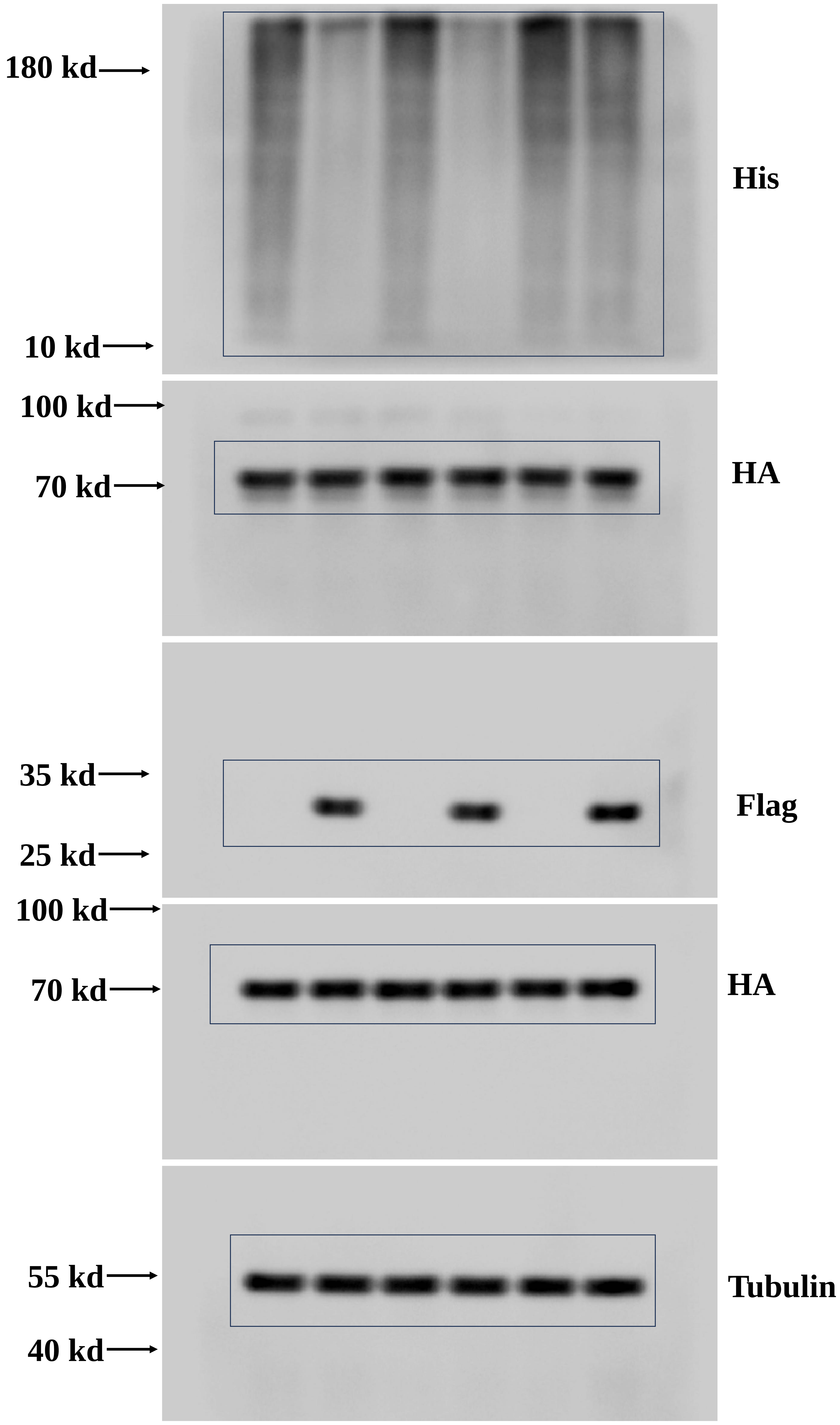
180 kd
10 kd
His
100 kd
70 kd
HA
35 kd
25 kd
Flag
100 kd
70 kd
HA
55 kd
40 kd
Tubulin

Figure S2A

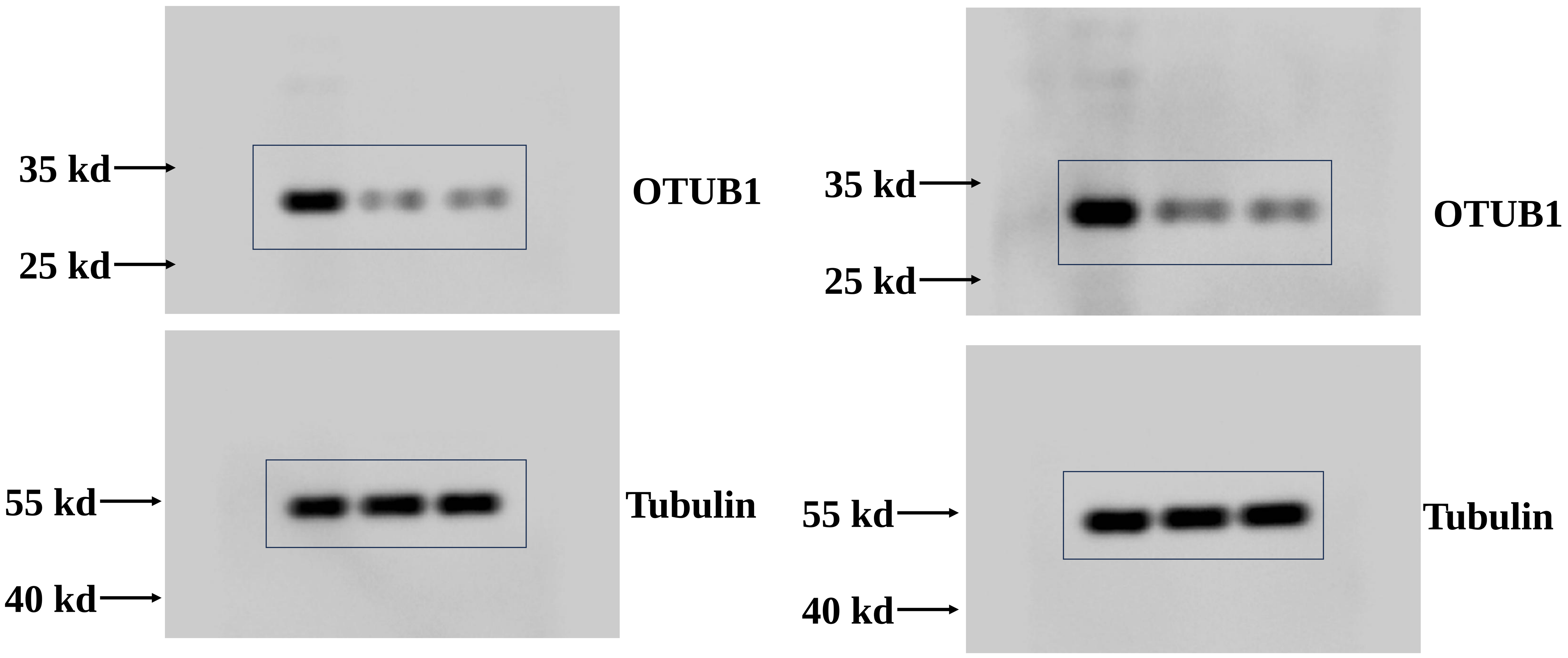

Figure S2B

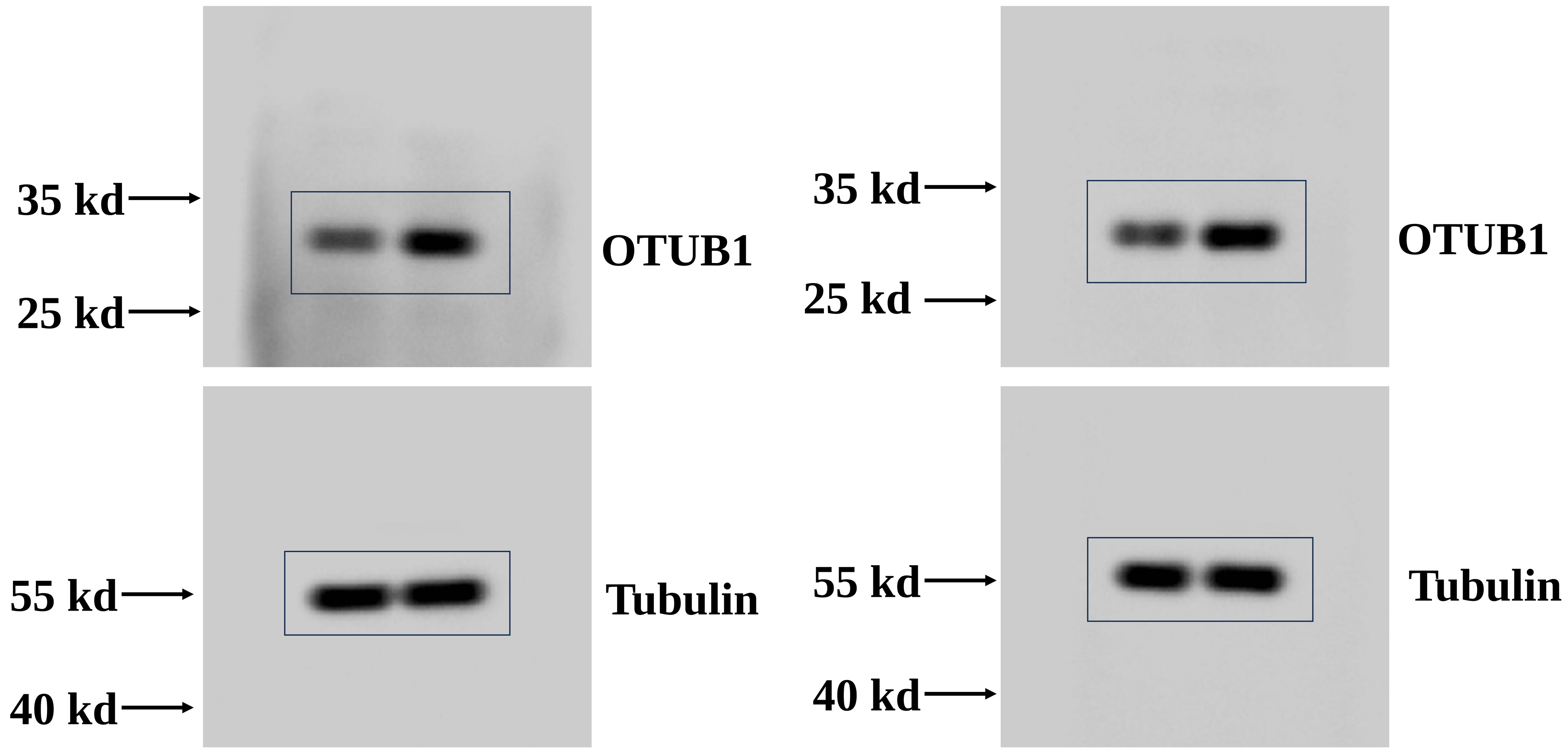

Figure S2E (left)

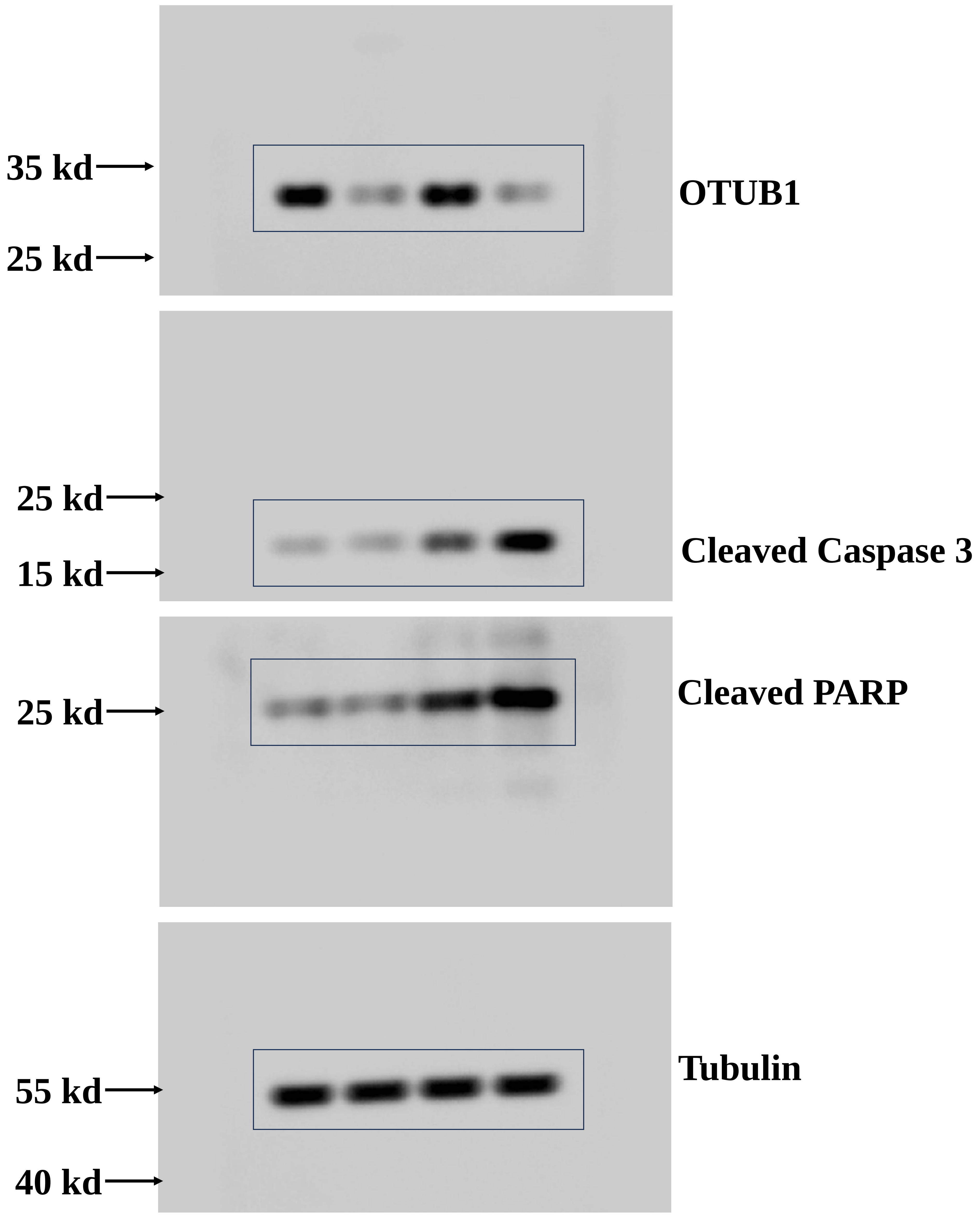

Figure S2E (right)

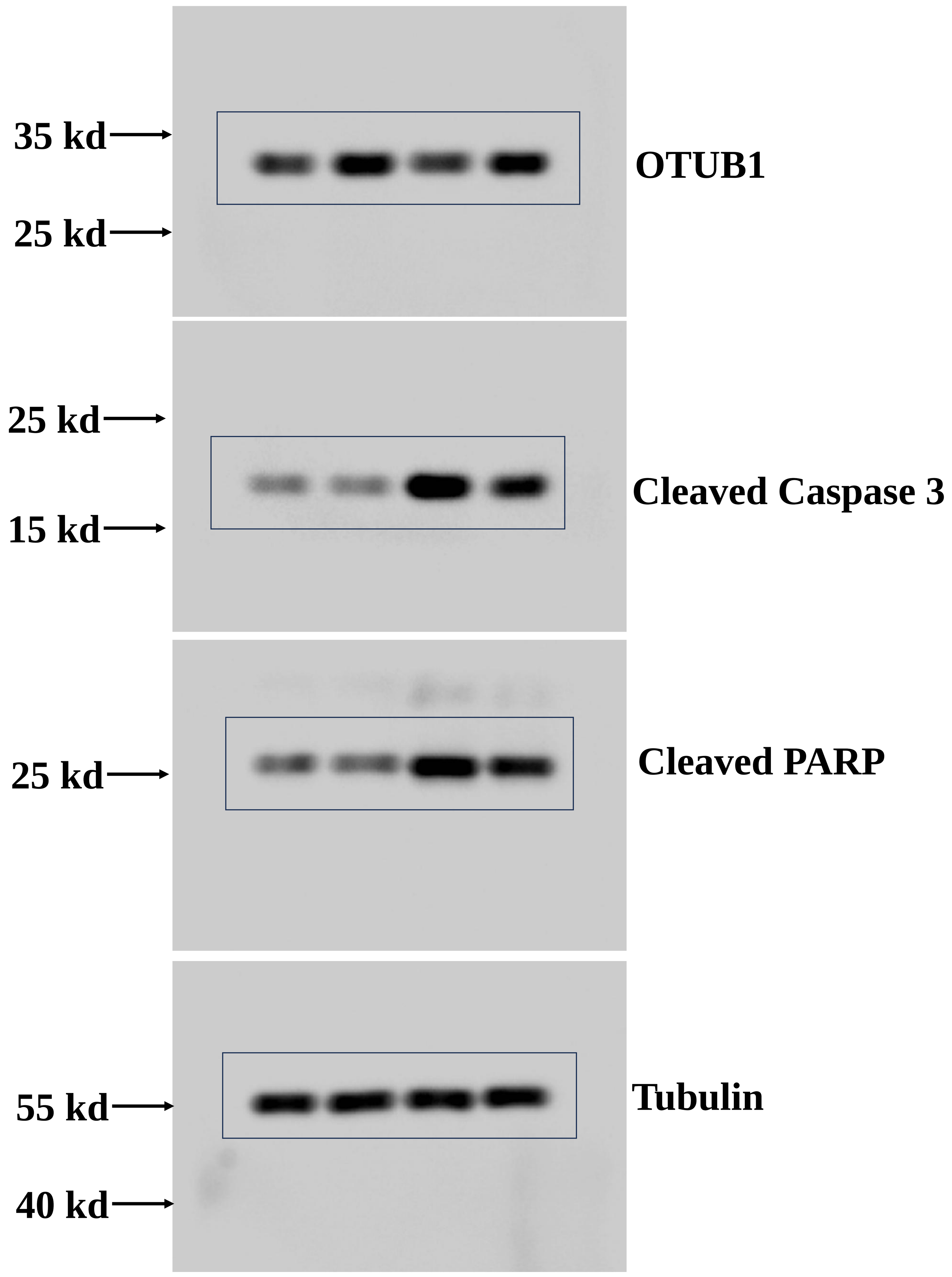

Figure S3A

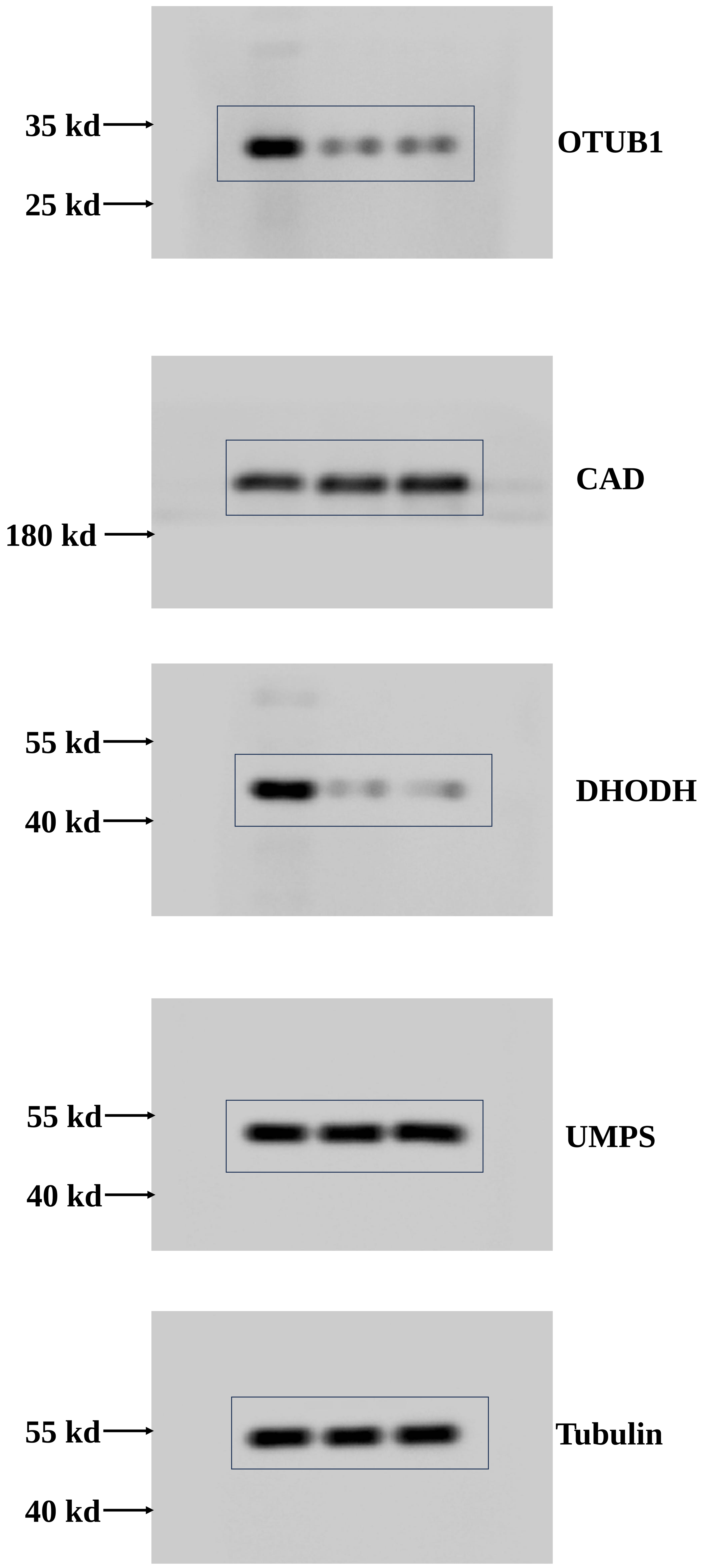

Figure S3C

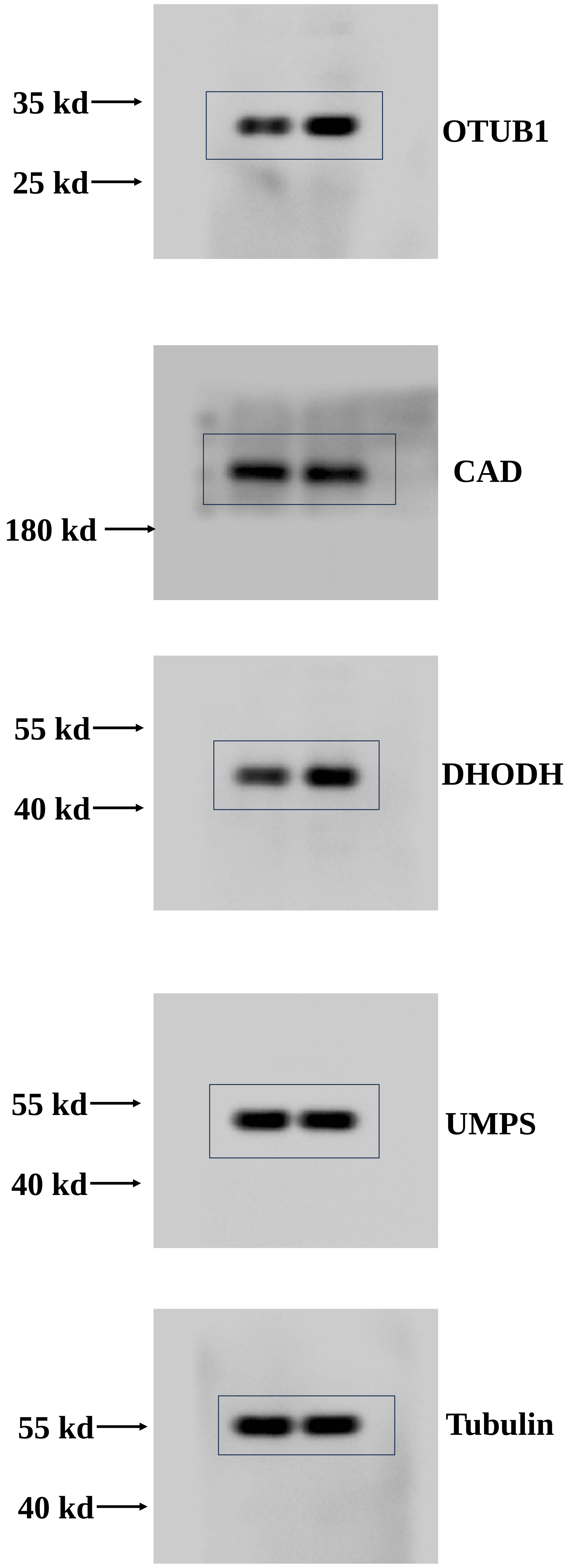

Figure S4A

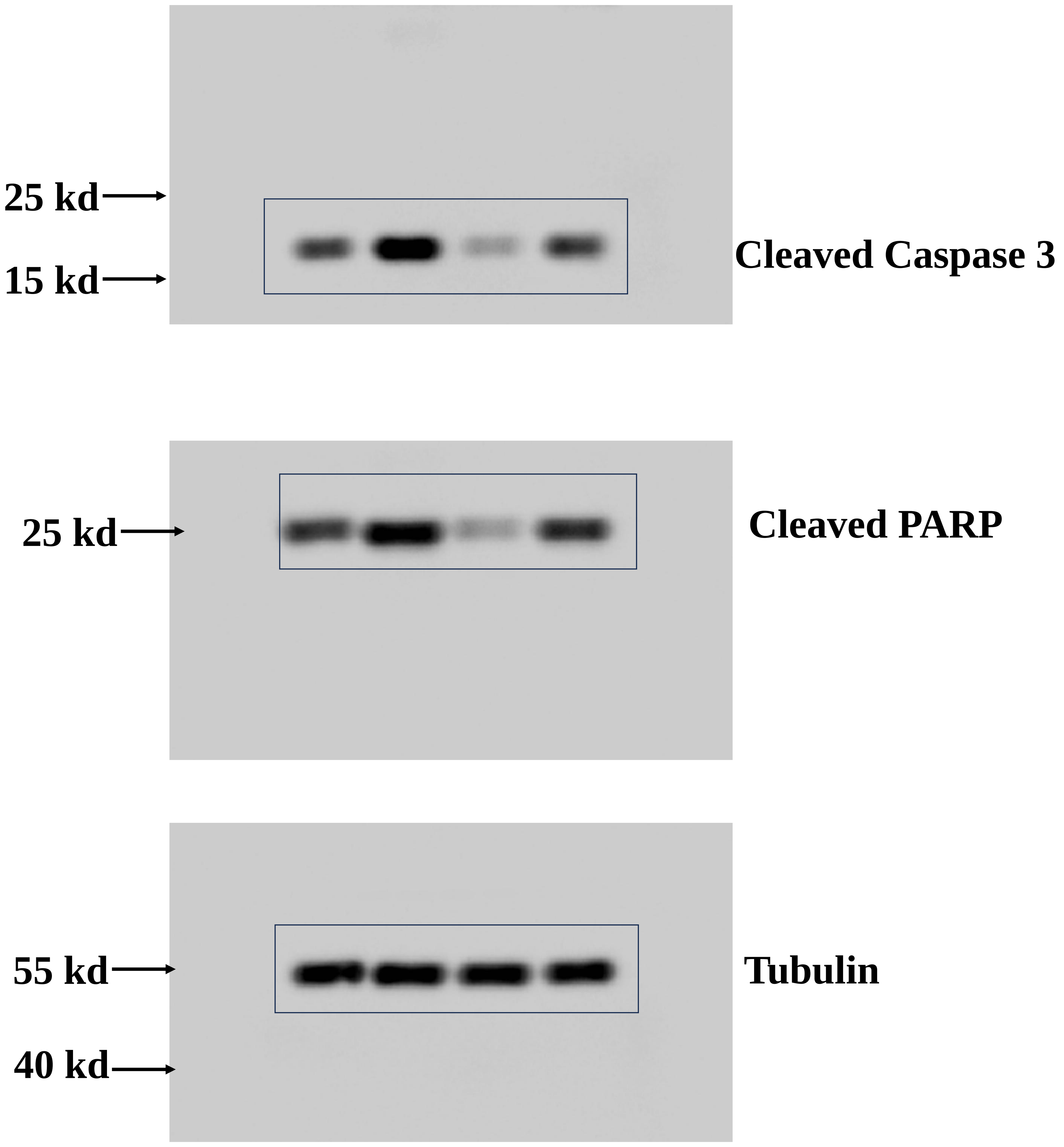

Figure S4F

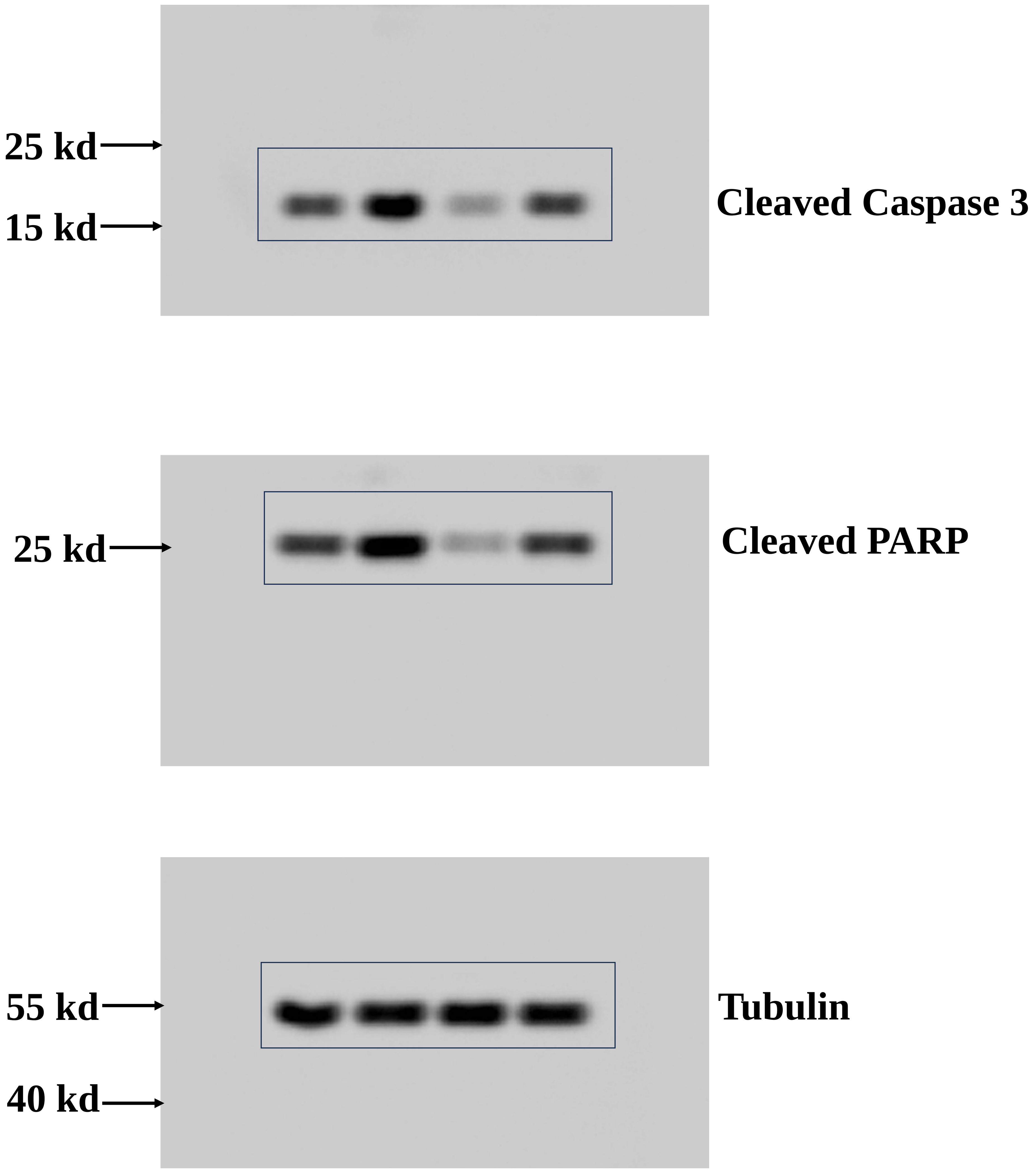

Figure S5B

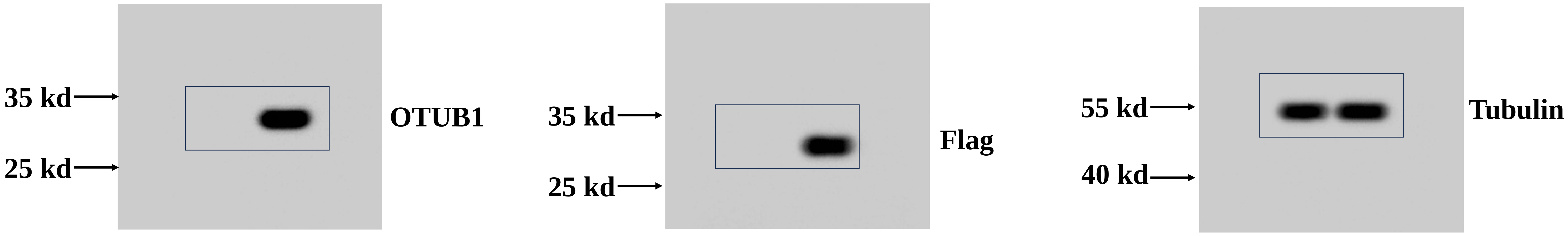

Figure S5E

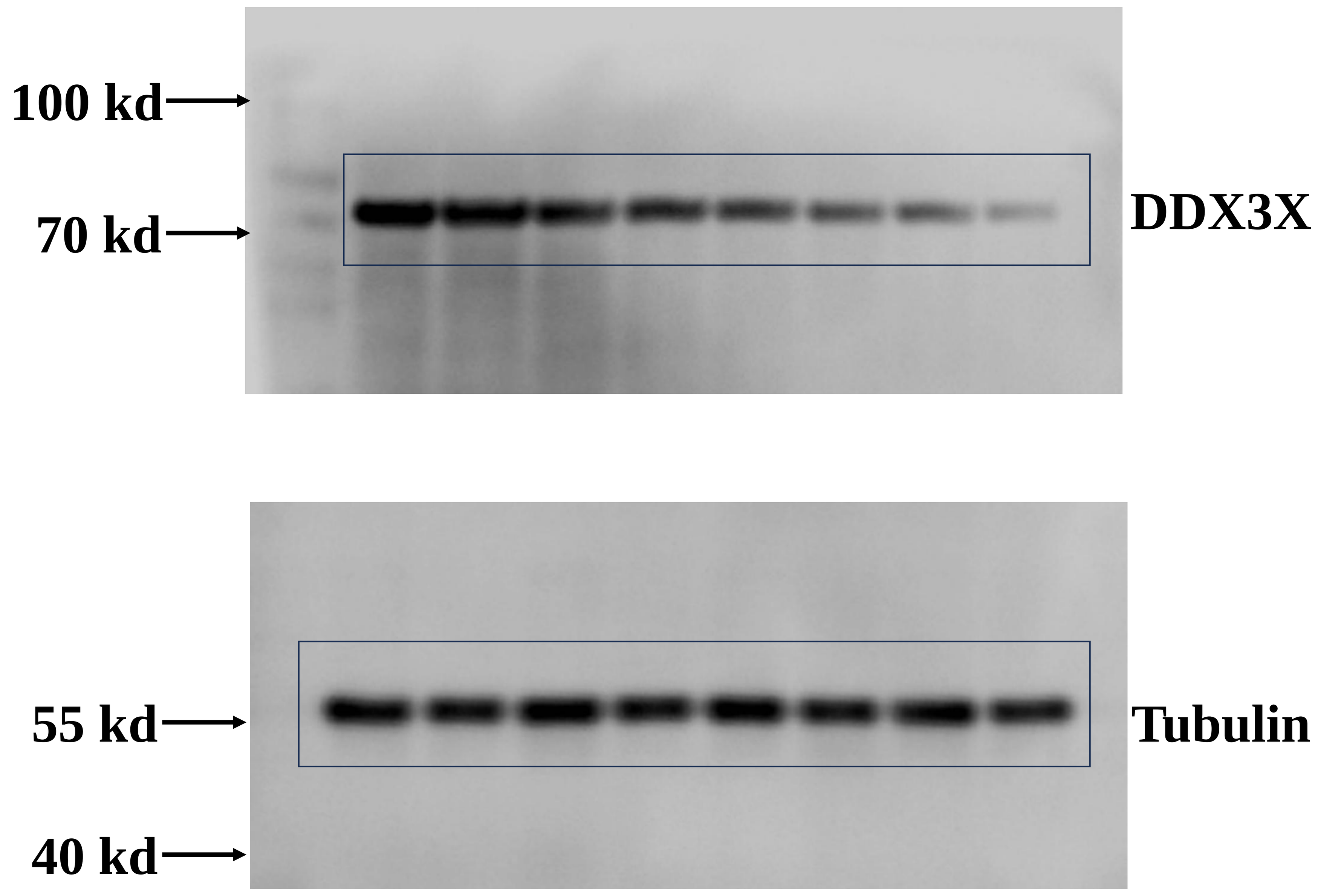

Figure S5F top

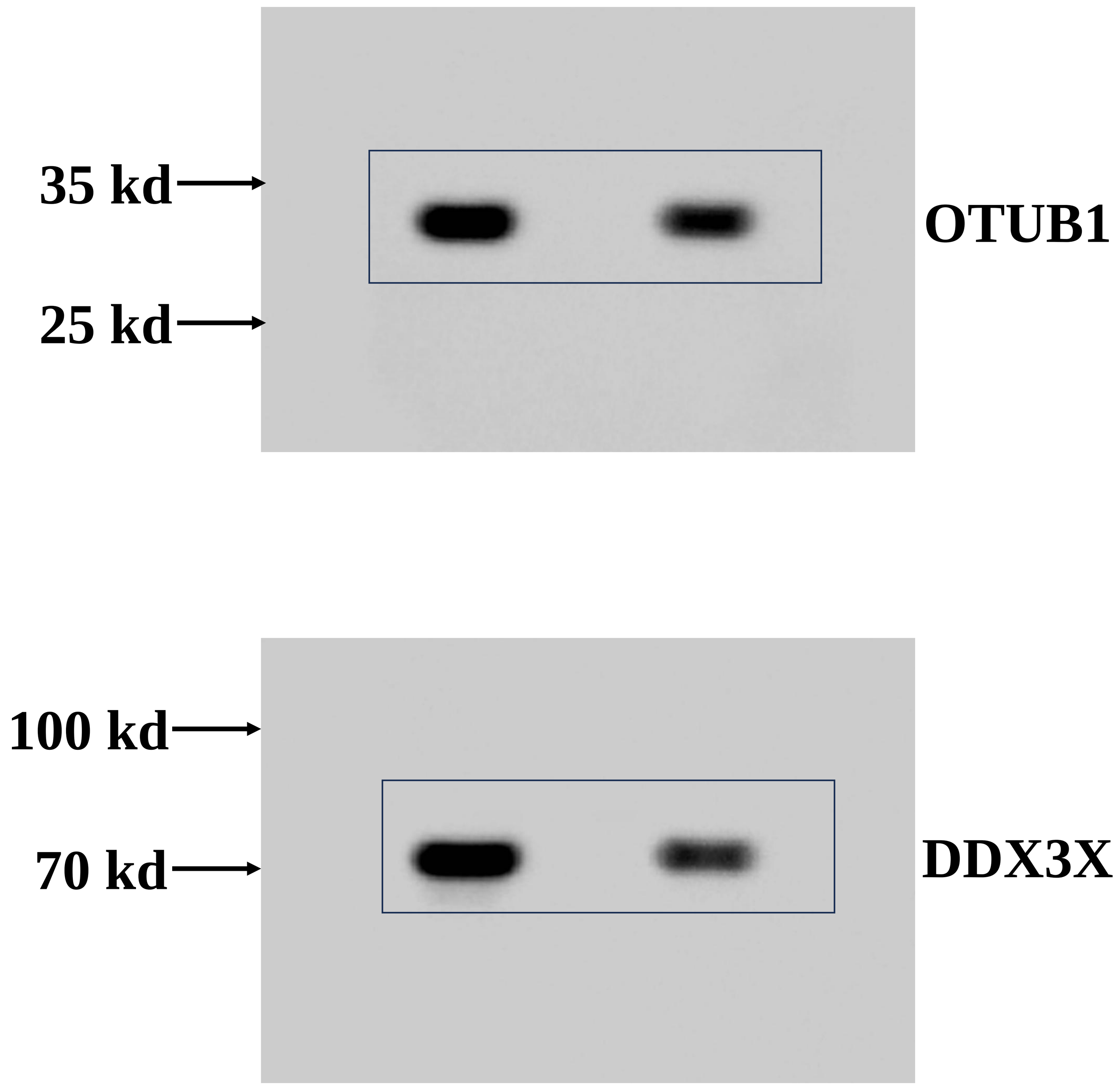

Figure S5F bottom

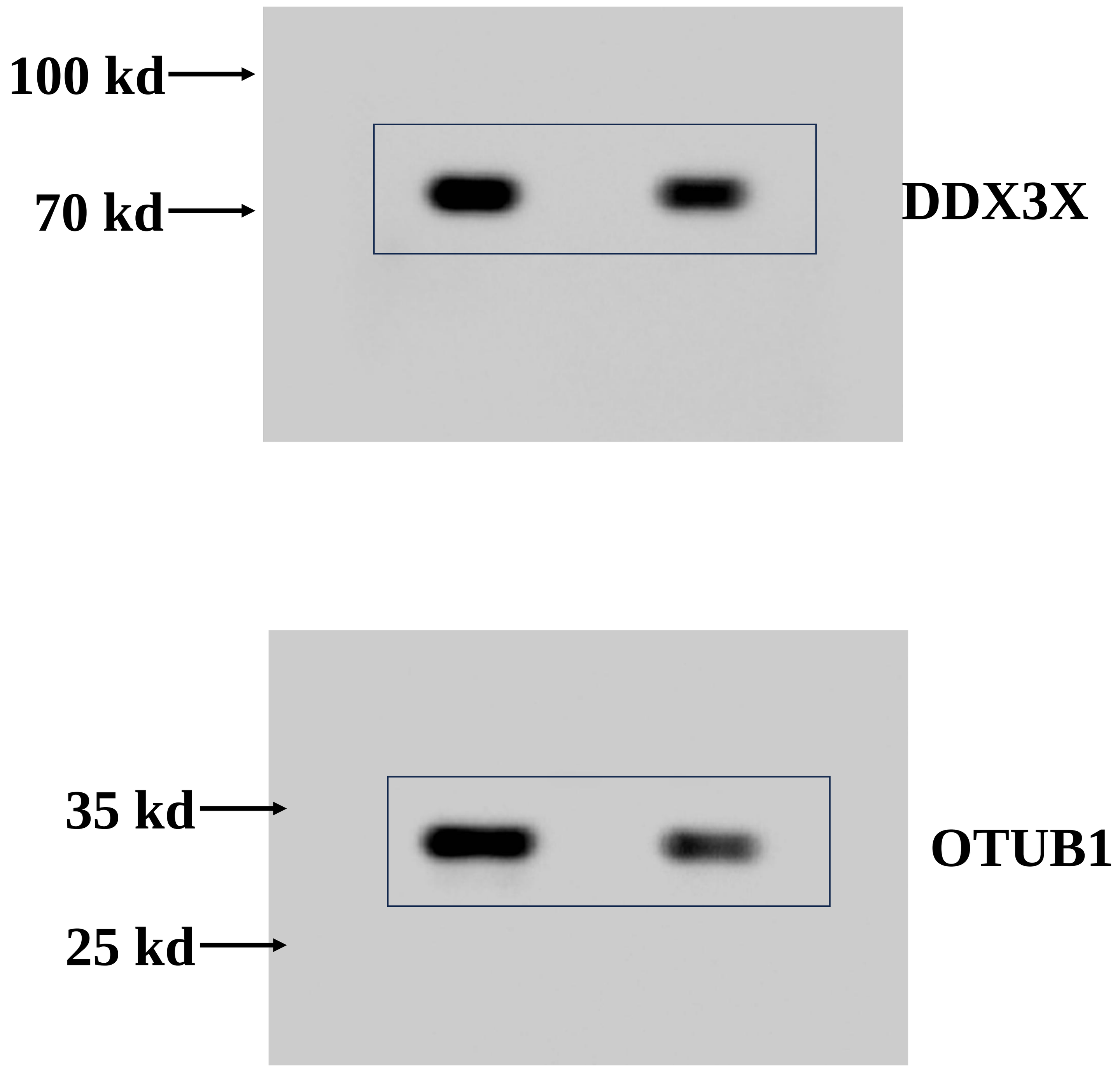

Figure S5G

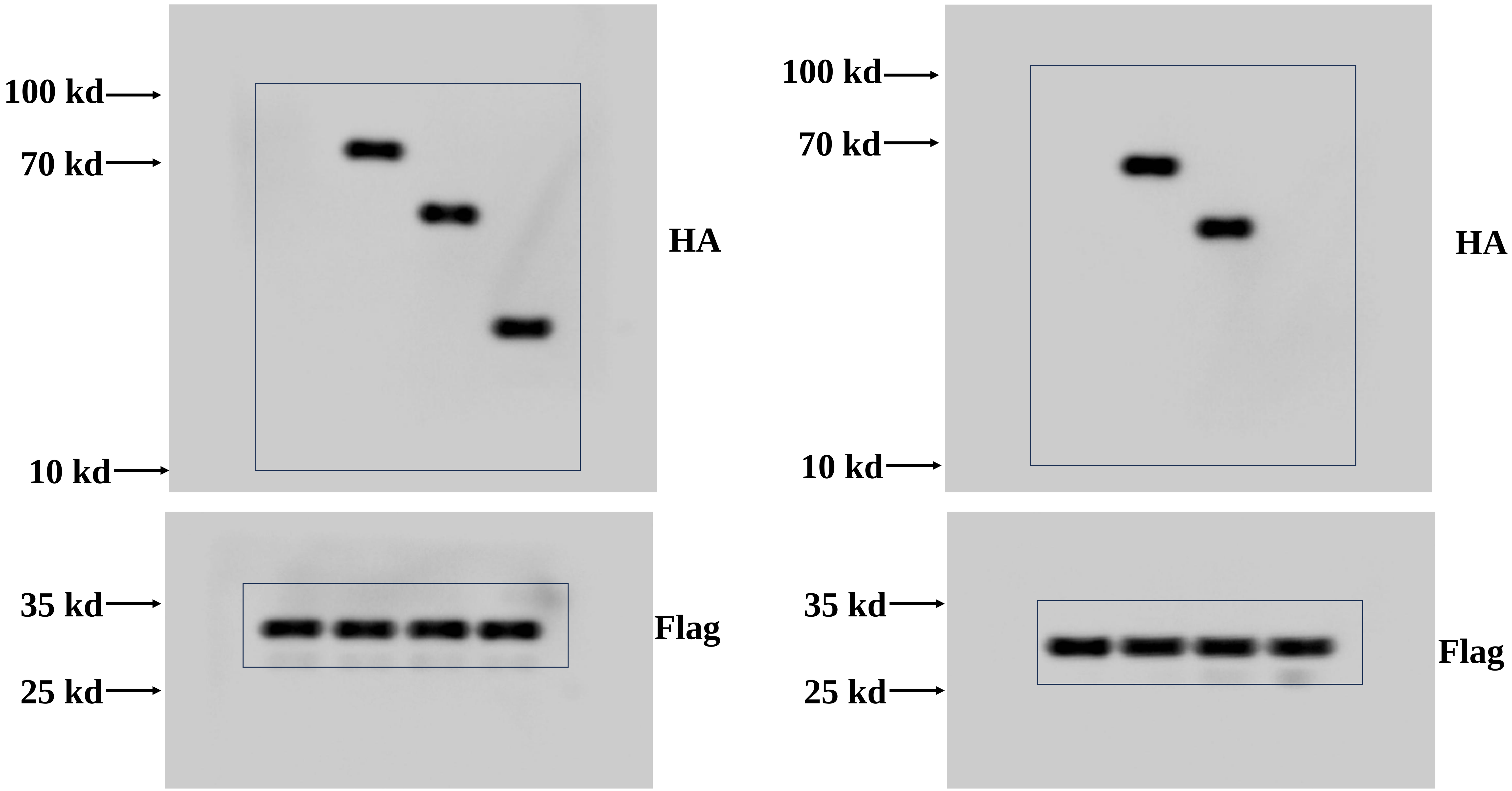

Figure S6A

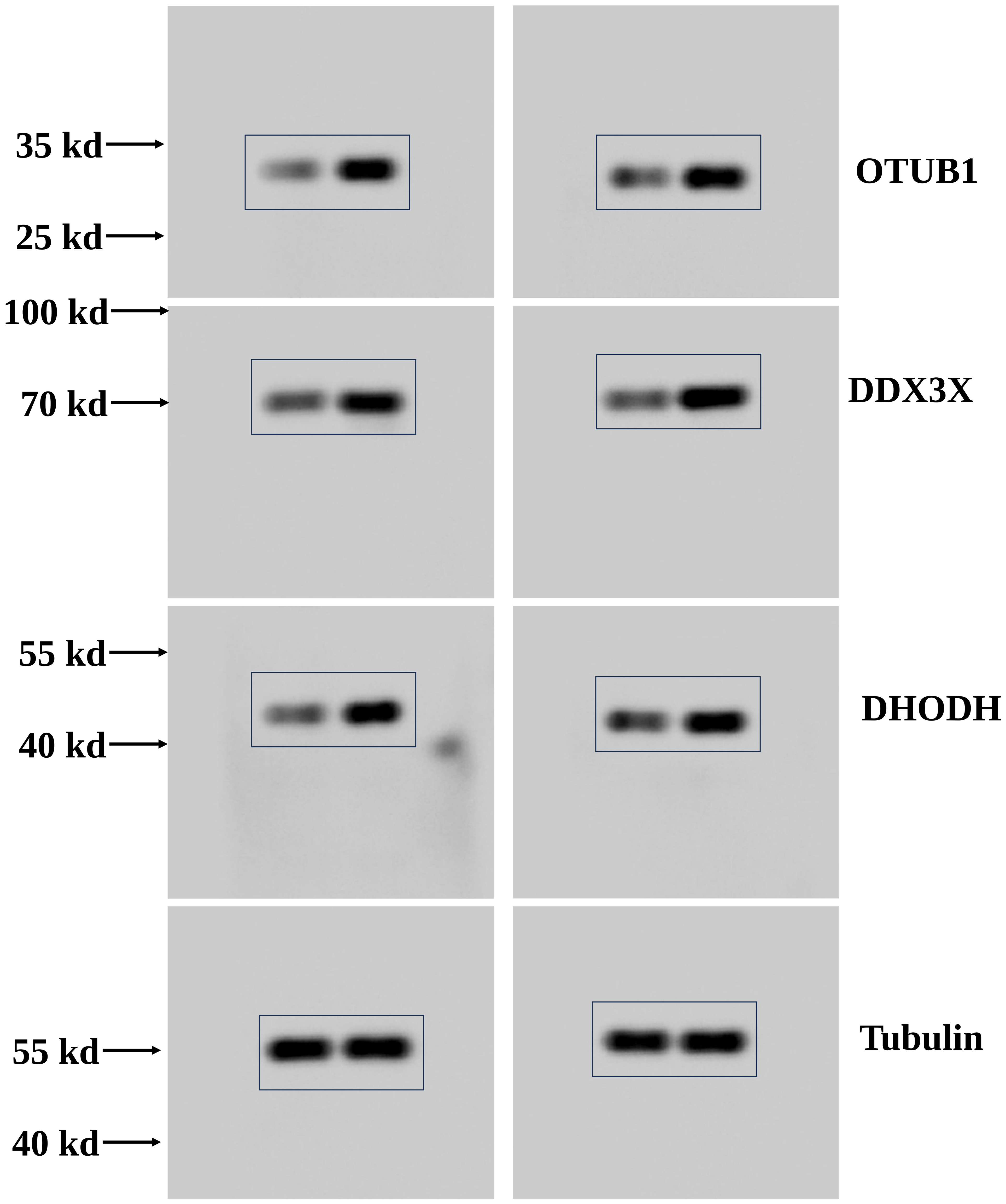

Figure S6C

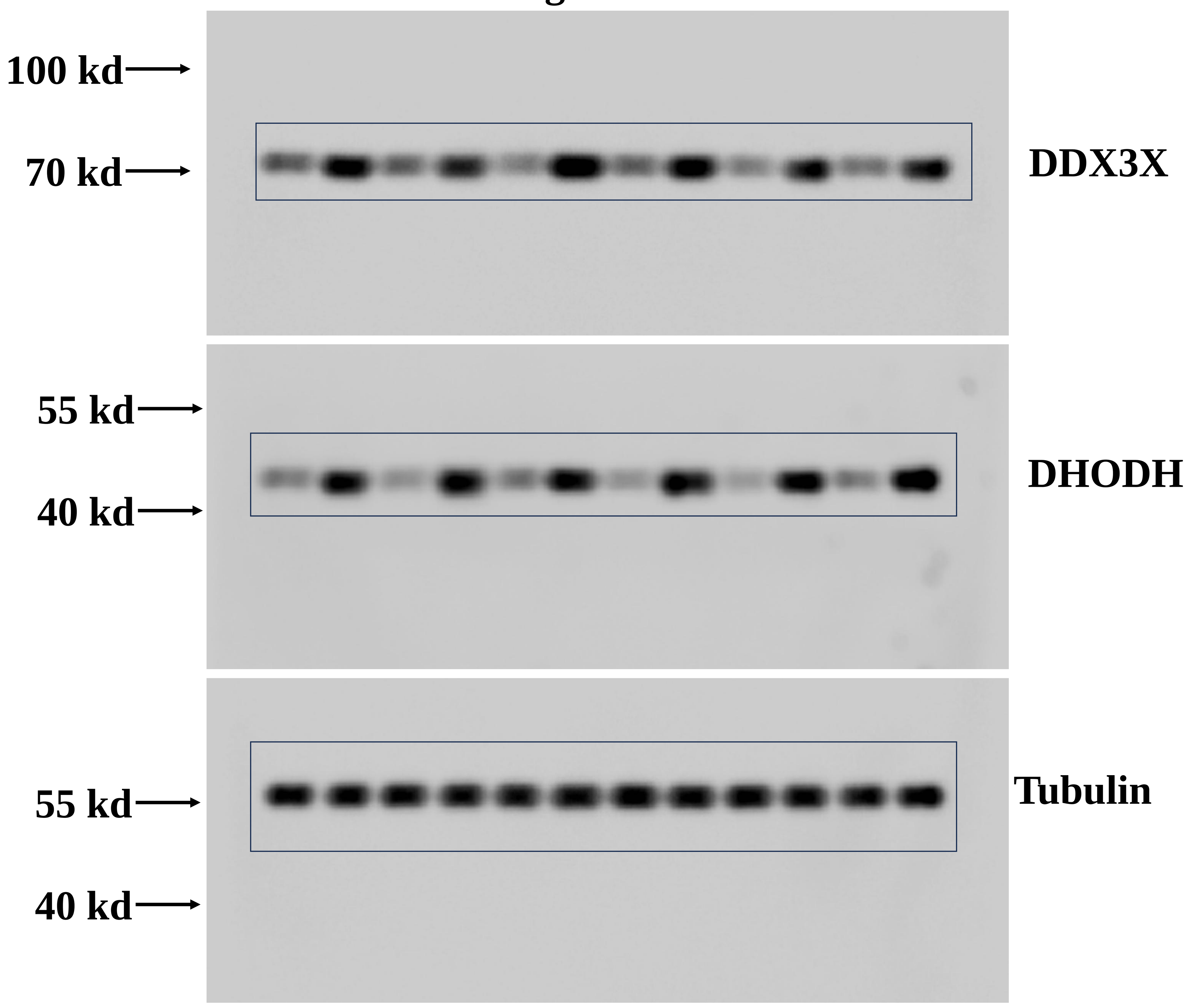

Figure S7A

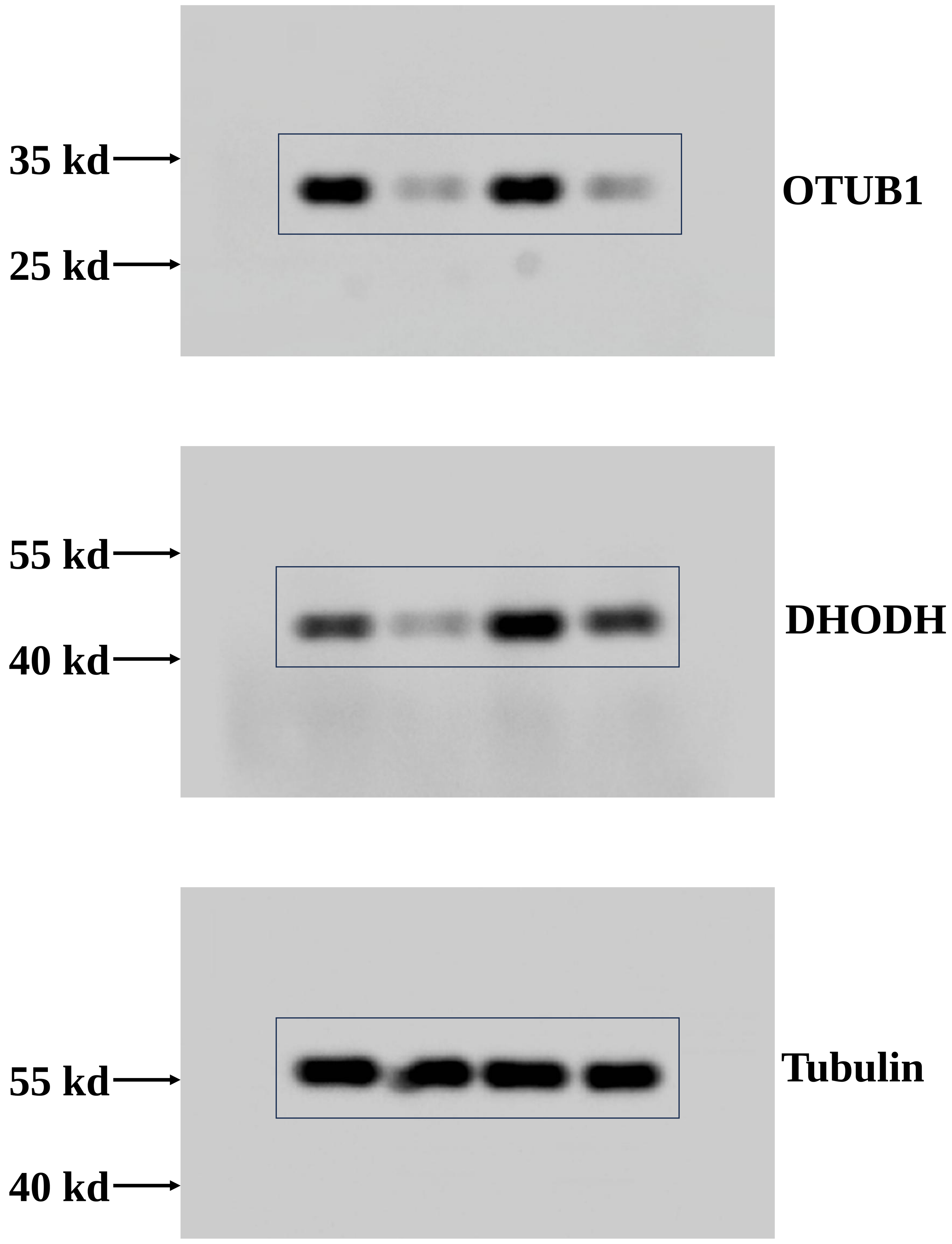

Figure S8B (left)

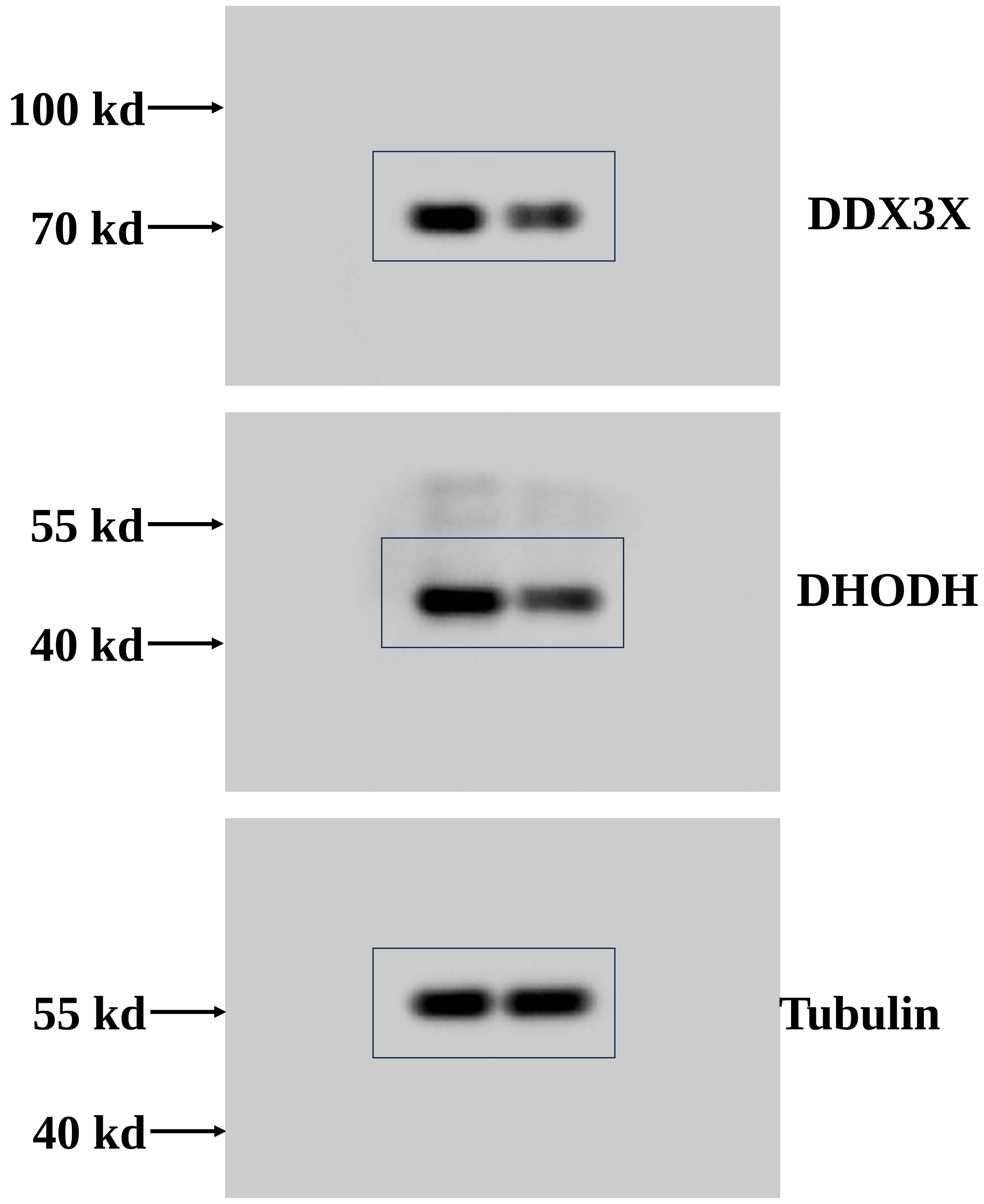

Figure S8B (right)

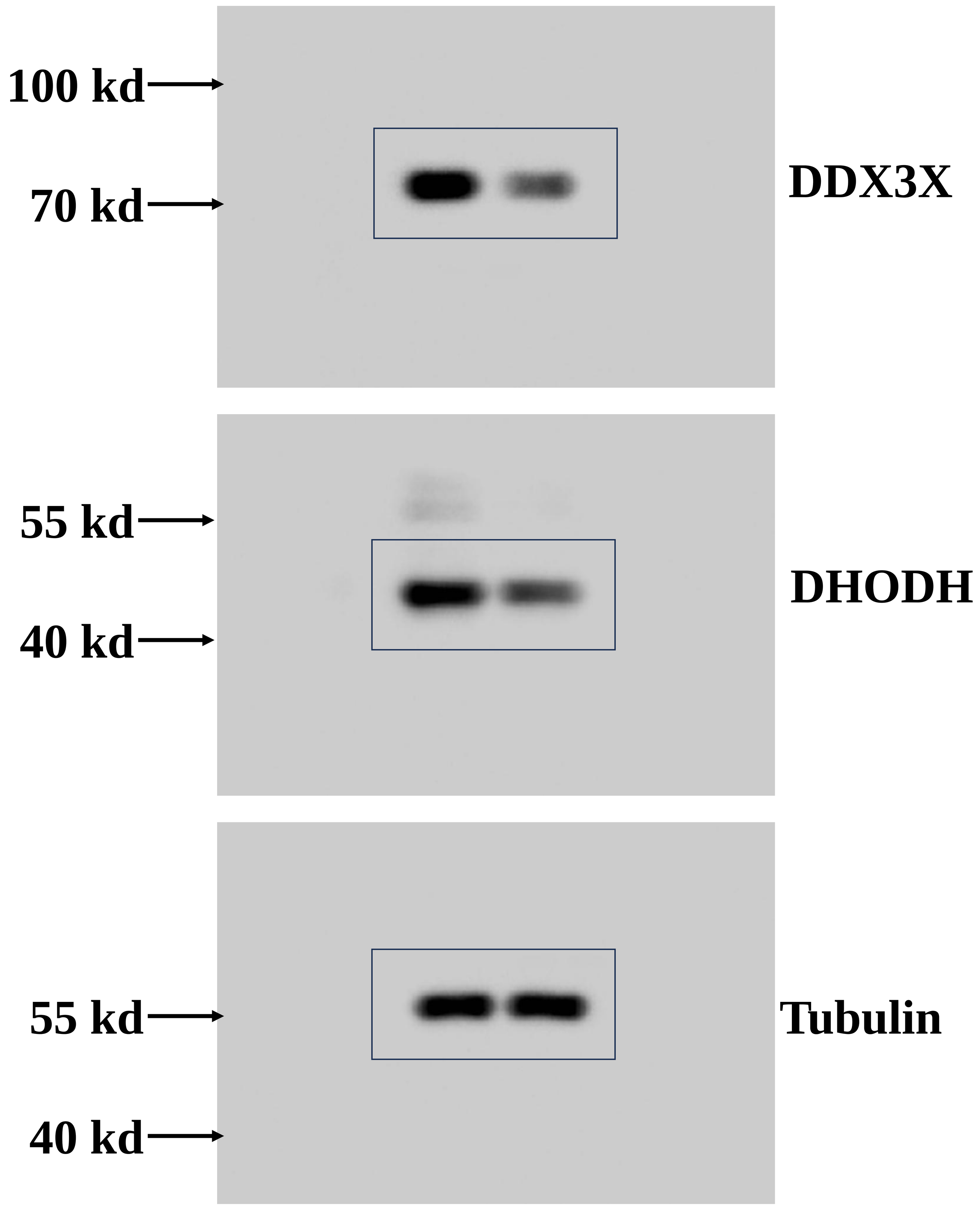

Figure S8C

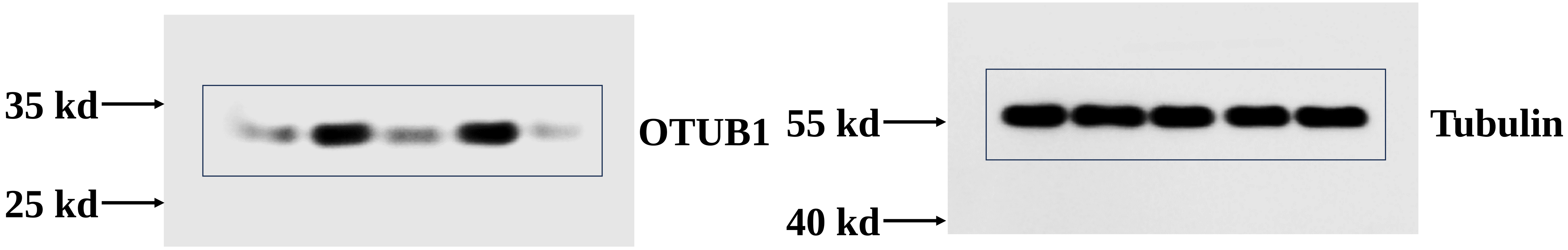

Supplement: Supplementary file 5 — Uncropped version of all Figures [file 41419_2025_8001_MOESM5_ESM.pdf]
